# Factors related to type 2 diabetic retinopathy and their clinical application value

**DOI:** 10.3389/fendo.2024.1484197

**Published:** 2024-11-20

**Authors:** Xue-Nan Lian, Ming-Ming Zhu

**Affiliations:** ^1^ School of Graduate Studies, Hebei North University, Zhangjiakou, China; ^2^ Department of Endocrinology, Handan Central Hospital, Handan, China

**Keywords:** type 2 diabetes, diabetic retinopathy, lactate dehydrogenase, risk factors, protective factors, logistic regression

## Abstract

**Objective:**

To compare the differences in clinical-related factors between patients with type 2 diabetes (T2DM) and those without diabetic retinopathy (DR) and to explore the risk factors or protective factors affecting DR in T2DM patients.

**Methods:**

We performed a retrospective analysis of 380 patients with type 2 diabetes admitted to Handan Central Hospital from June 2023 to May 2024. Clinical data collected included baseline characteristics, hematological tests, metabolic indicators, and information on diabetic complications and comorbidities.

**Results:**

Our findings identified intervention, neck vascular disease, bilateral lower limb venous thrombosis, high creatinine, high glomerular filtration rate, high chloride, high fasting C-peptide, and high lactate dehydrogenase as risk factors for DR. In contrast, High 2-hour postprandial C-peptide is a protective factor for diabetic retinopathy. A logistic regression model was constructed using stepwise regression to predict DR occurrence, achieving an accuracy of 0.80 and an AUC of 0.83.

## 1 Introduction

Diabetes is among the world’s leading chronic conditions. A 2013 survey indicated that 10.9% of adults in China are likely to have diabetes, with approximately 35.7% in the pre-diabetic stage (1). The age-standardized prevalence of blindness in DR increased significantly between 1990 and 2020 compared with undercorrected refractive errors, cataracts, age-related macular degeneration, and glaucoma. By 2040, the global population with diabetes is projected to exceed 600 million (2). As individuals with diabetes live longer, the prevalence of DR will also increase (3). In China, approximately 19.5 million individuals with diabetes have some form of DR, with about one-fifth of these cases progressing to vision-threatening diabetic retinopathy (VTDR) (4). A 2021 study in the United States reported a DR prevalence of 26.43% among diabetic patients, with VTDR affecting 5.06% (5).

Our understanding of DR remains incomplete, and most people with diabetes already have vision decline by the time they are offered vision screening. Therefore, identifying new predictors of DR is crucial for early detection and timely clinical intervention (6). Previous studies have identified the duration of diabetes; traditional risk factors such as glycated hemoglobin and blood pressure are not sufficient to explain the risk of DR (7).

Currently, fundus photography and fundus fluorescein angiography are the primary methods for diagnosing DR. Despite their effectiveness, these methods are costly, the procedures are cumbersome, and patient compliance is often low, limiting their widespread clinical use. At the same time, identifying and actively managing risk and protective factors for DR can significantly enhance patient outcomes in clinical practice.

Although numerous studies have investigated factors related to DR, comprehensive and integrated research on screening and predictive factors is still being determined. Specifically, there needs to be more research on this in the Handan area, highlighting the necessity of exploring its clinical application value. Therefore, this study took this as a starting point and compared the differences between DR patients and patients NDR from the perspective of general information, hematological test indicators and metabolic indicators, and examination indicators related to diabetes complications and comorbidities of type 2 diabetes patients, as well as studied the related factors affecting type 2 diabetic retinopathy, in order to provide application value for clinical work as much as possible, to predict the occurrence and development of DR at an early stage, and thus to take more comprehensive and reasonable intervention measures.

## 2 Materials and methods

### 2.1 Participants

This study collected patients with T2DM hospitalized at Handan Central Hospital from May 2023 to May 2024. Venous blood samples were collected in the morning after a 10-12 hour fast upon admission. Based on fundus photography results, patients without retinopathy (NDR group) were assigned to the control group, while patients with retinopathy (DR group) were assigned to the case group. Fundus examination outcomes were gathered by trained operators utilizing a non-mydriatic fundus camera, and professional ophthalmologists evaluated by the diagnostic criteria outlined in the “Guidelines for Clinical Diagnosis and Treatment of Diabetic Retinopathy in my country (2022).” All patients met the diabetes diagnostic criteria established by the World Health Organization (WHO) in 1999: 1. Presence of typical symptoms of diabetes and any of the following: Random blood glucose ≥ 11.1 mmol/L, Fasting blood glucose ≥ 7.0 mmol/L, Blood glucose ≥ 11.1 mmol/L two hours after oral administration of 75g of glucose, during a fasting state (defined as no caloric intake for at least 8 hours; random blood glucose refers to blood glucose measured at any time of the day) 2—absence of typical symptoms of diabetes but meeting any of the above diagnostic criteria on two separate occasions.

Inclusion criteria: 1) Participants meeting the 1999 WHO diagnostic criteria for type 2 diabetes. 2) Participants with complete medical records, including personal demographic information, clinical parameters, and fundus examination results.

Exclusion criteria: 1) Individuals with diabetes types other than type 2 (including type 1 diabetes, gestational diabetes, and secondary diabetes). 2) Individuals presenting with acute or severe chronic complications of diabetes. 3) Conditions influencing blood glucose levels: malignant tumors, thyroid disorders, hematologic diseases, and individuals experiencing stressful situations (e.g., infections, traumatic injuries, postoperative states). 4) Conditions affecting triglyceride levels: acute and chronic pancreatitis, obstructive jaundice, hypothyroidism, etc. 5) Participants with pre-existing eye disorders or those with unclear fundus imaging.

The laboratory department of Handan Central Hospital conducted measurements for the indicators above. Body mass index (BMI) was defined as weight in kg divided by height in m2. The calculation formula of the TyG index is LN [triglyceride (mg/dl) *plasma glucose (mg/dl)/2]. HOMA-IR index was derived from the formula fasting blood glucose level multiplied by fasting insulin level, divided by 22.5; Hypertension is systolic blood pressure ≥140 mmHg or diastolic blood pressure ≥90 mmHg. Examination indicators related to diabetes com-plications and comorbidities included neck vessel ultrasound and ultrasound of arteries and veins in both lower limbs.

### 2.2 Statistical analysis

The independent sample t-test was utilized for the significance analysis of continuous variables, while the chi-square test was employed to compare the significance of categorical variables. Statistical analyses were conducted using IBM SPSS Statistics 26, and the binary logistic regression model was established and evaluated using R language.

## 3 Results

Three hundred eighty patients diagnosed with type 2 diabetes were collected in this study, comprising 154 individuals in the DR group and 226 individuals in the NDR group. Significant differences were observed be-tween the two groups concerning indica-tors such as disease duration, as delineated in **Table 1**. There were no significant differences observed in other factors, such as age and gender. For detailed information, refer to **Supplementary Material S1**.

**Table 1:** Factors with significant differences between the two groups of patients.

	ToTal(n=380)	DR(n=154)	NDR(n=226)	P
BMI	25.25(23.26,28.09)	24.58(23.14,26.81)	25.67(23.46,28.40)	0.030
SBP	136.00(12500,15100)	142.50(128.00,157.00)	133.50(125.00,144.00)	0.001
COD	96.00(36.00,180.00)	120.00(72.00,216.00)	81.00(24.00,144.00)	<0.001
RBC	4.84(4.45,5.24)	4.74(4.36,5.09)	4.97(4.52,5.35)	0.001
HB	145.00(133.00,157.00)	142.00(131.00,152.00)	150.00(135.00,159.00)	<0.001
FIB	2.89(2.52,3.41)	2.99(2.58,3.52)	2.80(2.46,3.27)	0.028
ALT	20.50(15.00,30.00)	18.00(14.00,25.00)	22.00(16.00,34.00)	<0.001
AST	19.00(15.00,23.00)	18.00(15.00,22.00)	19.00(16.00,27.00)	0.026
AST/ALT	0.90(0.70,1.10)	1.00(0.20,1.20)	0.90(0.70,1.10)	0.001
ALB	43.20(40.63,45.10)	42.45(39.90,44.50)	43.45(41.20,45.60)	0.001
A/G	1.60(1.50,1.80)	1.60(1.40,1.80)	1.70(1.50,1.80)	0.018
TBIL	13.30(10.40,16.58)	12.85(9.70,15.70)	14.15(11.20,17.20)	0.004
UREA	5.50(4.60,6.67)	5.89(4.90,7.21)	5.33(4.45,6.50)	0.001
eGFR	103.47(93.62,112.32	100.66(90.45,110.49)	105.74(94.98,115.28)	0.002
APOAI	1.36(1.21,1.50)	1.39(1.25,1.52)	1.33(1.19,1.47)	0.021
2h-CP	3.74(2.07,5.91)	2.78(1.70,4.66)	4.47(2.54,6.87)	<0.001
2h-ins	28.52(17.20,59.22)	24.13(14.50,52.52)	32.33(18.16,62.40)	0.008
LDH	163.00(145.25,183.00)	168.00(150.00,190.00)	162.00(144.00,180.00)	0.009
HBTH	102.00(91.25,115.00)	107.50(97.00,118.00)	99.00(89.00,112.00)	<0.001
HBH	174(46%)	84(55%)	90(40%)	0.005
Intervention	331(87%)	146(95%)	185(82%)	<0.001
NV	261(69%)	122(79%)	139(62%)	<0.001
BVP	267(70%)	127(82%)	140(62%)	<0.001

BMI, Body mass index; SBP, systolic blood pressure; COD, Course of disease; RBC, Red blood cell count; HB, Hemoglobin; FIB, Fibrinogen; ALT, Alanine aminotransferase; AST, Aspartate aminotransferase; ALB, Albumin; A/G, Albumin/Globulin; TBIL, Indirect bilirubin; UREA, Urea; eGFR, Estimated glomerular filtration rate; APOA1, Apolipoprotein A1;2h-CP,2-hour post-prandial C-peptide; 2h-INS,2-hour postprandial insulin; LDH, Lactate dehydrogenase; HBTH, Hydroxybutyrate dehydrogenase; HBH, History of hypertension; NV, Vascular disease of the neck; BVP, Venous plaques in both lower extremities.

For continuous variables, those following a normal distribution are expressed as the mean (standard deviation), with the t-test used to determine significance. Variables not following a normal distribution are expressed as the median (P25, P75), with a non-parametric test used to determine significance. Binary categorical variables are ex-pressed as the number of cases (percentage), with the chi-square test used to determine significance.

**Figure 1:**
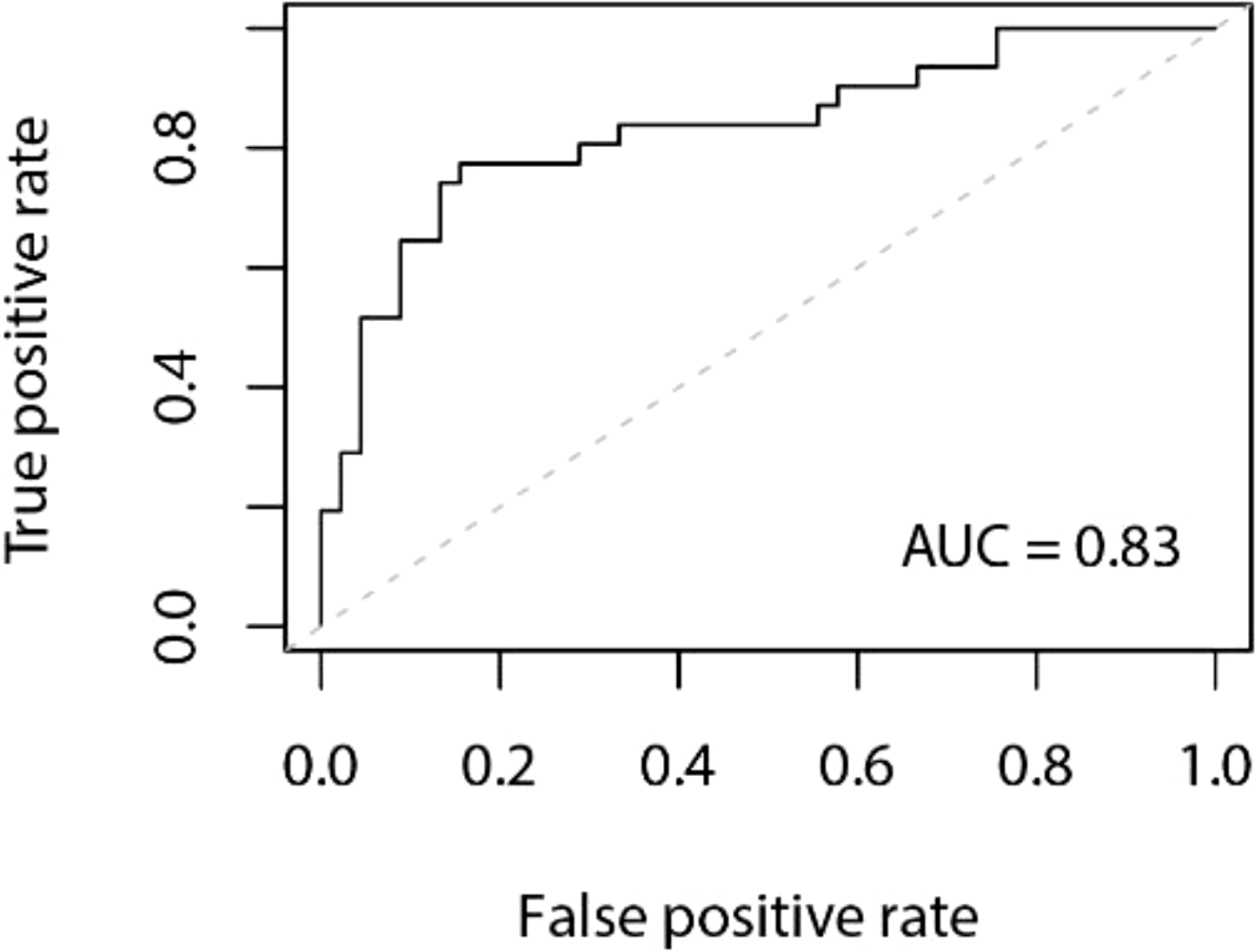
LR Model ROC Curve.

**Figure 2:**
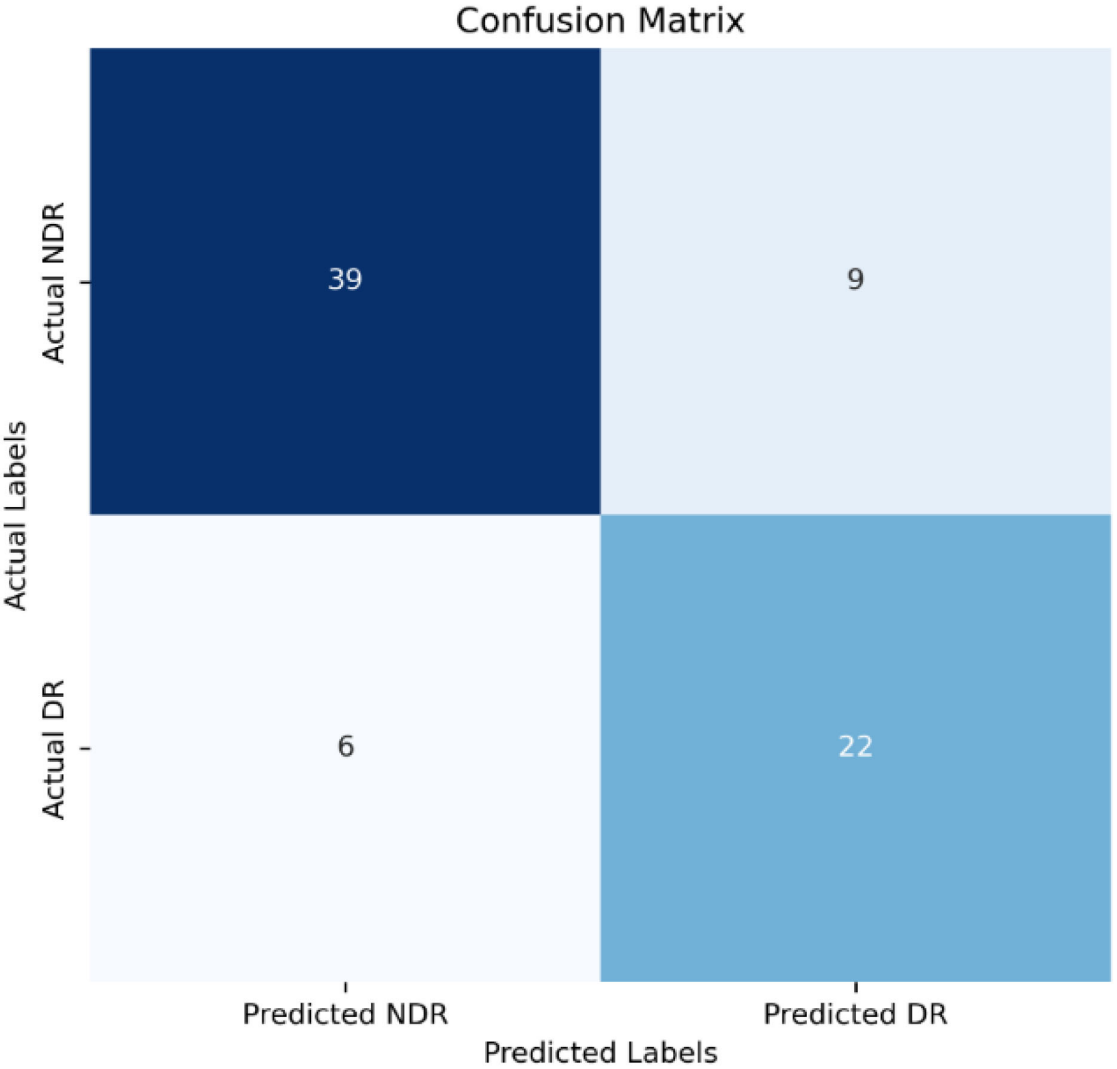
LR Model Confusion Matrix.

Model Establishment: The data in this study were split into a training set, and a validation set at a ratio of 8:2. Stepwise regression was employed to select variables, and a logistic regression model was constructed based on these selected variables. The model achieved an accuracy of 0.80 in the validation set, with a 95% confidence interval (CI) of 0.70 to 0.89. The area under the Receiver Operating Characteristic (ROC) curve (AUC) was 0.83. The ROC curve is depicted in **Figure 1**, and the confusion matrix is shown in **Figure 2**.

In addition, to further assess the clinical practicality and predictive performance of the logistic regression model, we conducted decision curve analysis (DCA) and calibration curve analysis. The DCA curve for the logistic regression model was plotted (**Figure 2**) to determine the net benefit of the model at various decision thresholds. The DCA curve demonstrated that the model performs well across different thresholds, indicating its substantial clinical application value.

**Figure 3:**
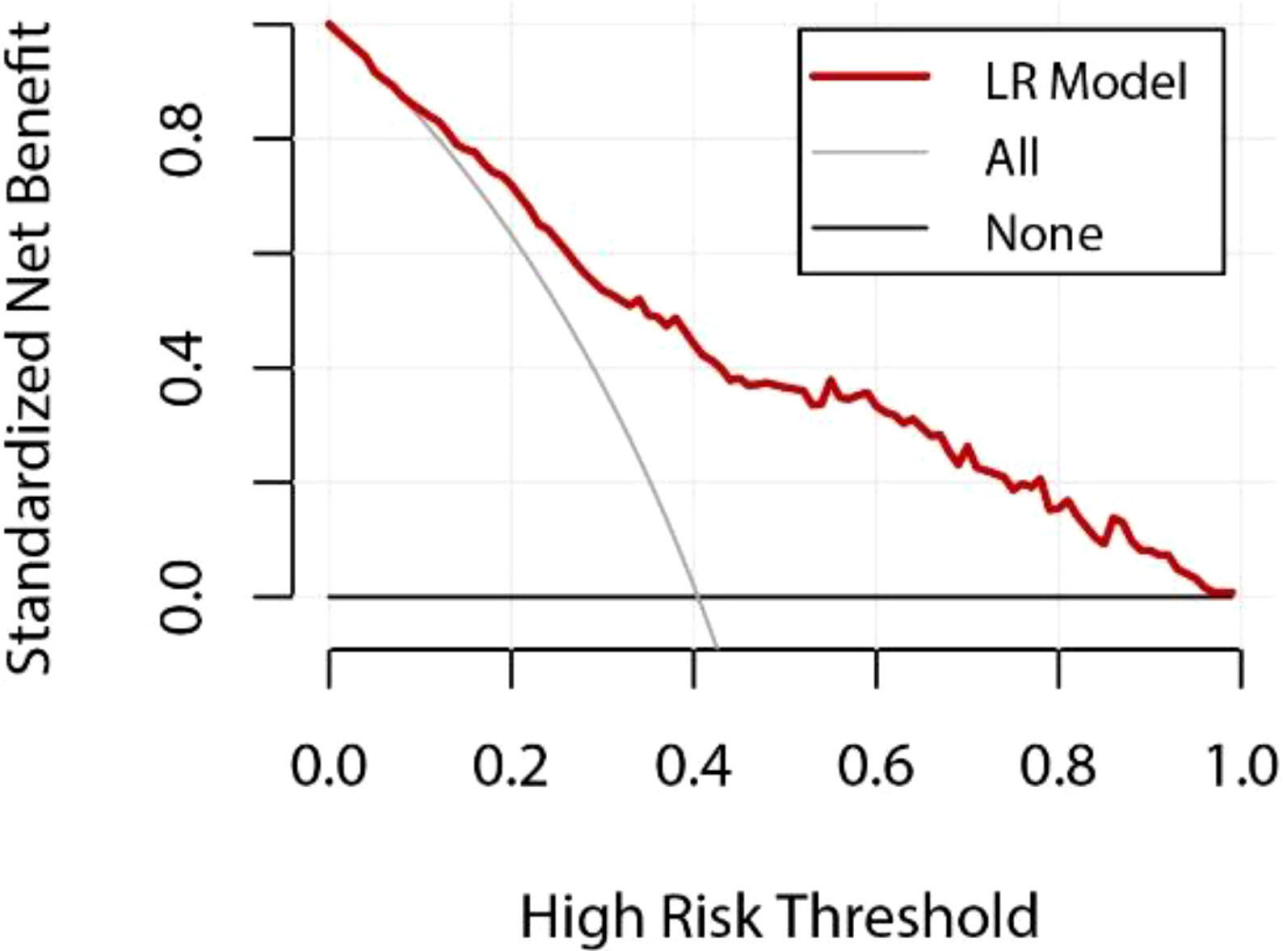
LR Model DCA Curve.

**Figure 3** LR Model DCA Curve Y-axis: net benefit of standardization; X-axis: Relationship between high risk thresholds; The red curve represents the performance of the logistic regression (LR) model; The gray line represents the net benefit if all patients are assumed to be at high risk; The black line represents the net benefit if no patient is assumed to be at high risk; The decision curve helps to evaluate the clinical value of the model at different risk thresholds, and the larger the area below the red curve, the higher the actual benefit of the model.

The calibration curve assesses the consistency between the model’s predicted probabilities and the actual outcomes. By comparing the predicted probabilities with the observed results, we generated a calibration curve to evaluate the degree of calibration of the model (**Figure 3**). The calibration curve in this study indicates that the logistic regression model we constructed demonstrates good predictive ability and accuracy.

**Figure 4:**
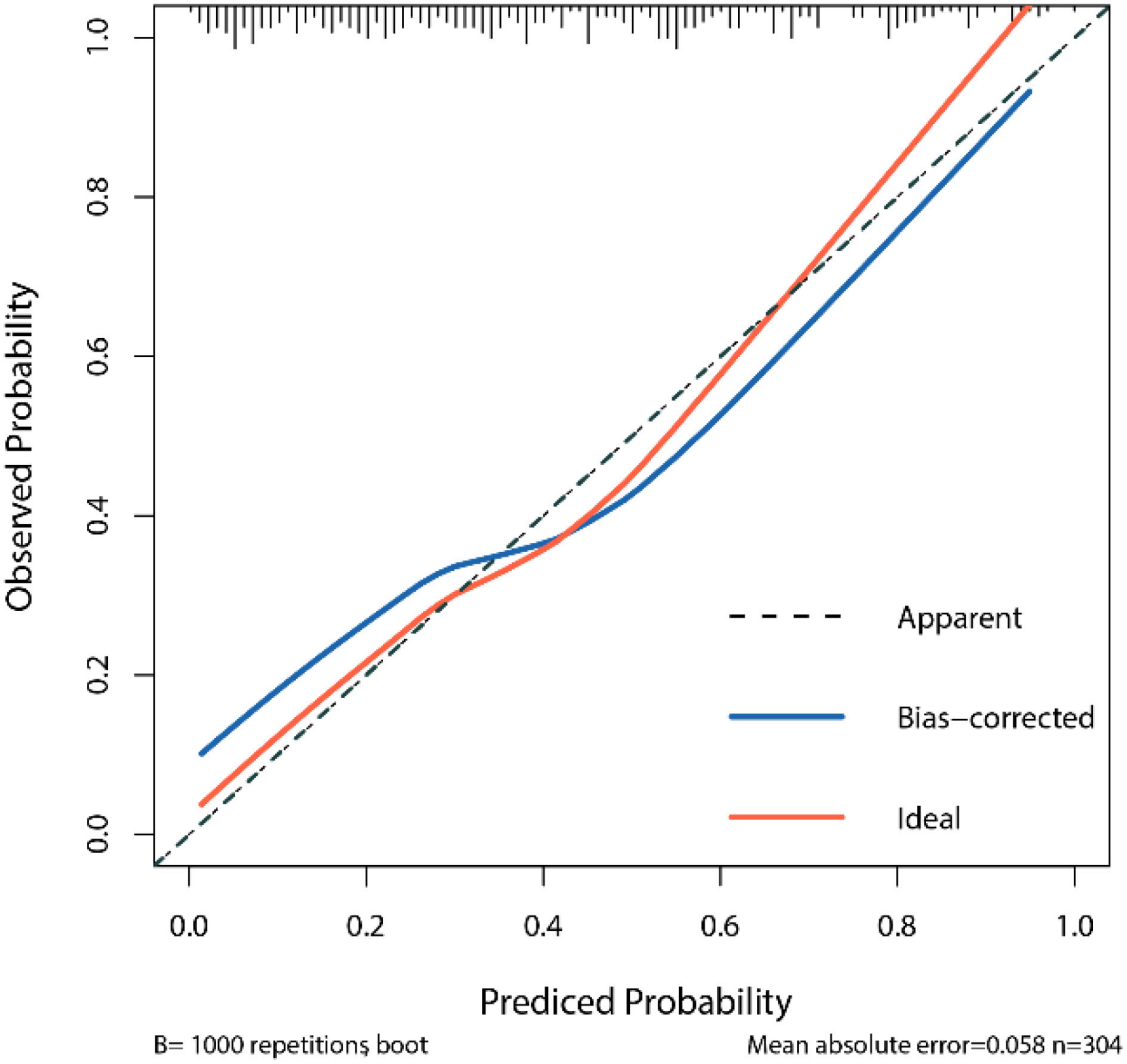
LR Model Calibration Curve.

**Figure 4** LR Model Calibration Curve X-axis: prediction probability of the model; Y-axis: probability of actual observation; Dashed lines (Apparent) show exactly the same line in an ideal state; The red curve (Ideal) is the ideal calibration curve; The blue Bias-corrected curve is the correction curve after 1000 repeated sampling through Bootstrap. The closer the blue correction curve is to the red ideal curve, the better the prediction performance of the model.

The factors associated with diabetic retinopathy (DR), as identified by the logistic regression model, are presented in **Table 2**. The results indicate that several variables are significantly associated with an increased risk of DR:

- Intervention (OR=3.127, 95% CI: 1.148-9.565).
- Neck vascular disease (OR=2.240, 95% CI: 1.092-4.685).
- Bilateral lower limb venous thrombosis (OR=2.151, 95% CI: 1.051-4.052).
- High creatinine (CREA) levels (OR=1.033, 95% CI: 1.007-1.062).
- High estimated glomerular filtration rate (eGFR) (OR=3.127, 95% CI: 1.148-9.565).
- High chloride (CL) levels (OR=3.127, 95% CI: 1.148-9.565).
- High fasting C-peptide (FCP) levels (OR=3.127, 95% CI: 1.148-9.565).
- High lactate dehydrogenase (LDH) levels (OR=3.127, 95% CI: 1.148-9.565).

**Table 2:** Factors related to DR.

	OR	2.50%	97.50%
Intervention	3.127	1.148	9.565
NV	2.240	1.092	4.685
BVP	2.151	1.051	4.502
CREA	1.033	1.007	1.062
eGFR	1.049	1.016	1.086
CL	1.127	1.004	1.270
FCP	1.523	1.076	2.156
2h-CP	0.730	0.614	0.852
LDH	1.010	1.000	1.020

CREA, Creatinine; CL, Chloride; FCP, Fasting C-peptide.

Conversely, high 2-hour postprandial C-peptide (2h-CP) levels were protective against diabetic retinopathy.

## 4 Discussion

After excluding confounding factors, this study confirmed that neck vascular disease (NV), bilateral lower extremity venous thrombosis (BVP), high creatinine (CREA), high eGFR, high chloride (CL), high fasting C-peptide (FCP), and high lactate dehydrogenase (LDH) are risk factors for DR. In contrast, high C-peptide 2 hours after a meal (2h-CP) can prevent diabetic retinopathy, there were significant differences in BMI, FIB, UREA, and 2h-ins between the DR group and the non-DR group. The presence of multiple risk factors suggests that the patient may be at a higher risk of developing DR. For patients showing abnormal indicators, we prioritize screening for DR. Therefore, we recommend incorporating these indicators as part of routine screening in clinical practice.

The common biological pathway through which DM and its complications cause cellular damage in microvessels involves hyperglycemia-induced mitochondrial reactive oxygen species (ROS) production. This leads to nuclear DNA strand breaks, which subsequently activate adenosine diphosphate ribose polymerase (PARP). PARP modification reduces the activity of glyceraldehyde-3-phosphate dehydrogenase (GAPDH), triggering the activation of the polyol pathway, promoting the formation of advanced glycation end-products (AGEs), and activating protein kinase C (PKC), mitogen-activated protein kinase (MAPK), and hexosamine pathways (8–10).

OS is closely linked to DR, contributing to damage in organs such as the heart, kidneys, and retina when the body’s balance is disrupted (11). LDH plays a pivotal role in this process. LDH is typically released in minimal amounts, but its secretion increases with heightened cell membrane permeability, correlating with the severity of cell damage. When intraretinal glucose levels rise, Müller glial cells sustain increased glycolysis by releasing lactate and enhancing mitochondrial oxidative metabolism in photoreceptor neurons, which leads to elevated levels of LDH in the blood and retina (12). In a rat study, treatment with Triphala churna significantly reduced LDH levels, thereby delaying the progression of DR (13). Recent studies have also shown that hyperglycemia upregulates the calcium-binding protein Iba-1, leading retinal microglia to promote the release of LDH in a dose-dependent manner. This triggers pyroptosis in retinal microglia, ultimately causing neurovascular damage associated with DR (14).

In hyperglycemic and hypoxic conditions, LDH levels rise in retinal cells (15). Studies have identified LDH as a biochemical marker for predicting the onset of DR due to oxidative stress (11, 13, 16). Elevated LDH levels correlate closely with glycated albumin (ALB) and insulin antibody levels (13, 14, 17). However, a linear regression analysis found no significant association between LDH and T2DM risk. LDH can also be a biomarker to monitor short-term blood glucose variability (18). Recently, Yang et al. conducted a multivariate regression analysis on risk factors for DR in the United States. They found that higher LDH concentrations (>134 U/L) significantly increased the risk of DR in subjects with diabetes mellitus (19). However, their study only partially aligns with our findings. In addition, the diagnosis of DR is based only on questionnaires and lacks the results of retinal imaging examinations so that the accuracy may be insufficient. This study makes up for this shortcoming. Consistently, there is a correlation between. The series of cellular damage reactions in hyperglycemia are closely tied to the activation of ROS. Studies have shown that when mitochondrial voltage is impaired by uncoupling protein 1 (UCP-1) or when superoxide is degraded by manganese superoxide dismutase (MnSOD), hyperglycemia does not activate these pathways (8). Large-scale cohort studies could further confirm these findings.

Moreover, during NPDR, inflammatory cytokines such as IL-1β, IL-6, IL-8, TNF-α, and monocyte chemoattractant protein-1 (MCP-1), produced by activated endothelial cells, glial cells, and neurons contribute to early neuronal necrosis in the diabetic retina. During PDR, soluble cytokine receptors (sIL-2R) and matrix metalloproteinases (MMPs) further exacerbate inflammation (20). In diabetic macular edema (DME), vascular endothelial growth factor (VEGF), hepatocyte growth factor (HGF), IL-6, and MCP-1 increase vascular permeability and promote angiogenesis in DR. Additionally, intracellular adhesion molecules, along with endothelial and glial cells, induce the upregulation of VEGF, TNF-α, IL-6, and IL-1β, leading to retinal ischemia and hypoxia (21).

Fibrinogen (FIB), regulated by IL-6, TNF-α, and IL-1, has been shown to play a role in DR (22). While Tomić et al. found no association between FIB and DR (23), Zhuang et al. reported a significant relationship (24). Our study also observed significant differences in FIB levels in DR patients. Furthermore, IL-6 stimulates CRP synthesis, and high-sensitivity C-reactive protein (hs-CRP) has been linked to more severe DR (25). However, Gouliopoulos et al. did not find this correlation (26), which aligns with our findings. Understanding how inflammatory factors contribute to DR pathogenesis will likely be an important focus of future research.

Diabetic kidney disease (DKD) is associated with DR (8, 27), both being microangiopathies, though their exact pathological link remains incompletely proven. Among patients with T2DM, an association is observed between urine albumin/creatinine ratio (UACR) and DR. In contrast, no significant association has been established with eGFR (28). Wang Jianyong and colleagues have reported that the severity of DKD, abnormal eGFR, and UACR are associated with an increased risk of DR in T2DM patients (29). The conflicting results may be attributed to differences in genetics, climate, geographical environment, sample size, inclusion criteria, and the renal function indicators used in the studies. To address these discrepancies, future research could benefit from conducting multicenter cohort studies to provide more conclusive evidence. In a large cross-sectional study, Zhang Guihua et al. found that elevated serum creatinine levels are linked with DR (30). Similarly, a nationwide DR screening study in South Korea highlighted the correlation between serum creatinine levels and DR, suggesting a role for renal function in the progression of DR (31). Therefore, further research is essential to validate these findings. In our study, urea and eGFR were found to be associated with DR. A previous cross-sectional study also reported that lower eGFR levels were linked to the presence and severity of DR, although not with DME (32). Zhang Junlin and colleagues identified significant associations between proteinuria, hematuria, baseline eGFR adjustment, severity of glomerulopathy, and a diabetes mellitus history exceeding 10 years with the risk of DR (33).

Early studies suggested a negative correlation between C-peptide levels and the occurrence and progression of DR (34). However, some studies have found no association between the eGFR and DR, necessitating further large-scale studies for confirmation. Our results indicate an association between C-peptide and DR. C-peptide is secreted by pancreatic β cells *in vivo* and is closely related to insulin resistance, serving as a clinical marker for evaluating pancreatic islet function. Nevertheless, its relationship with vascular complications of T2DM remains incompletely understood. Logistic regression analysis by Wang Yan and colleagues involving 4,793 diabetic patients demonstrated a positive correlation between C-peptide and the occurrence and progression of cardiovascular disease (CVD), yet a negative correlation with DR progression. Higher C-peptide levels were associated with a lower prevalence of DR (35). Higher C-peptide levels correspond to a reduced risk of diabetic microvascular complications (36). Recent studies have demonstrated a negative correlation between postprandial C-peptide levels and DR, which aligns with our findings (37).

Peripheral vascular disease (PVD) is primarily characterized by the formation of atheromatous plaques in the arteries of the lower limbs. These plaques result from lipid deposition and carbohydrate accumulation within the arterial intima, promoting fibrous tissue proliferation and calcium deposition (38). The development of plaques in the neck and lower limb blood vessels can cause arterial stenosis and occlusion, leading to tissue ischemia and impaired vascular function. DR, as a microvascular complication, is similarly influenced by vascular dysfunction. In patients with T2DM, carotid artery plaques have been shown to significantly increase the modification of endothelial cell proteins through the hexosamine pathway (8). Some studies also suggest that lipid metabolism disorders heighten the risk of DR (39). Furthermore, a multicenter observational study of 2,068 T2DM patients found that HDL levels greater than 40 mg/dL were associated with an increased risk of DR, a novel finding not previously reported (40).

Previous studies have indicated that patients with DR are independently associated with carotid artery plaques compared to those without DR (41). Consistent with our findings, our study identified bilateral lower extremity arteriovenous plaques (BVP) as a risk factor for DR, highlighting the significance of both carotid and bilateral lower extremity arteriovenous plaques in the development of DR.

A British study suggested that higher BMI and waist circumference (WC) could be potential risk factors for microvascular complications in diabetes (42). Age, BMI, SBP, duration of diabetes, and glycated hemoglobin (HbA1C) are independent risk factors for VTDR (43). Other studies have also identified both SBP and diastolic blood pressure as independent risk factors for DR in patients with T2DM (44). Although previous cross-sectional studies have suggested an association between pulse pressure, SBP, and DR, the causal relationship remains unclear (45). Patients with longer diabetes duration, lower education levels, and lower income are at an increased risk of developing DR (46). Additionally, inadequate control of blood glucose and blood pressure further heightens this risk. Therefore, lifestyle interventions, such as improved diet and increased physical activity, can help reduce the likelihood of DR (47). However, in our study, age, diabetes duration, HbA1c levels, blood lipids, and blood pressure did not show significant differences between the DR group and the control group. Interestingly, BMI exhibited a significant difference, which may be attributed to factors such as sample size, genetics, education and income levels, dietary habits, and physical activity. In future research, we plan to include a larger sample size, and the use of prospective cohort studies may provide stronger evidence.

DR often lacks apparent symptoms in its early stages (48). Many patients seek medical attention only when they develop serious conditions. DR represents a severe chronic complication of diabetes, significantly impacting later recovery and prognosis. Therefore, enhancing early screening and prevention for DR patients in clinical practice is paramount. Despite the positive findings, our study has several limitations: First, it is retrospective, which may introduce biases that limit causal inferences. Secondly, only case data from our department were included with a limited number of patients. These factors may limit the generalizability of our research findings. Specifically, influences such as age, race, genetics, access to healthcare, education and income levels, climate, geographical environment, eating habits, and exercise, among other lifestyle factors, may have introduced some degree of interference in our results. To determine whether these factors affect the occurrence of DR, future studies should employ multicenter, large-scale prospective cohort designs to improve the generalizability and robustness of the findings.

## 5 Conclusions

The regression model we developed achieved an accuracy of 0.80 in the test set and an area under the ROC curve (AUC) value of 0.83, demonstrating high clinical utility. Building on previous research, we identified that the combined presence of neck plaque and lower limb arteriovenous plaque is a robust predictor for DR. Previous studies have underscored the potential of LDH as a DR risk factor, and significant differences in BMI, FIB, UREA, and 2h-ins were observed between the DR and non-DR groups. However, our study is limited by its sample size, necessitating validation through more extensive studies to better inform clinical practice.

## Data Availability

The original contributions presented in the study are included in the article/**Supplementary Material**. Further inquiries can be directed to the corresponding author.
