# Supplementary material for: Factors related to type 2 diabetic retinopathy and their clinical application value
Source: Front Endocrinol (Lausanne). 2024 Nov 20;15:1484197. doi: 10.3389/fendo.2024.1484197 (PMC11614660; doi:10.3389/fendo.2024.1484197)
Supplement: Supplementary file 1 [file DataSheet1.pdf]

DRlabel	Gender	Age	Height	BMI	SBP	DBP	Smoke
1	1	54	1.72	20.28123	112	82	0
1	1	54	1.7	25.0865	173	92	1
1	1	53	1.7	26.6436	126	90	1
0	1	64	1.83	28.36752	122	88	1
0	1	80	1.65	24.60973	143	83	0
0	0	49	1.62	24.57702	125	87	0
1	1	60	1.72	19.94321	176	103	1
1	0	53	1.63	26.34649	135	89	0
0	0	54	1.62	23.62445	128	74	0
1	1	54	1.7	25.95156	150	86	1
0	1	36	1.7	22.49135	154	117	1
0	1	42	1.78	19.25262	100	81	1
0	1	38	1.86	31.21748	128	82	1
0	1	36	1.86	27.74887	124	93	0
1	0	52	1.6	23.4375	160	106	0
1	1	41	1.75	29.38775	144	105	0
1	1	34	1.77	25.53545	132	84	1
1	0	54	1.58	24.03461	108	72	0
1	1	60	1.75	20.57143	140	86	0
1	0	48	1.58	27.23922	130	91	0
0	1	44	1.72	31.43591	154	108	0
0	1	56	1.7	23.87543	142	99	0
0	1	54	1.71	24.62296	167	93	0
0	0	49	1.62	20.00457	116	77	0
1	0	67	1.6	33.00781	128	81	0
0	1	42	1.81	31.43982	180	129	0
0	1	67	1.64	23.05176	140	67	1
1	1	58	1.72	22.9854	194	106	0
1	0	52	1.55	27.05515	131	80	0
0	0	41	1.6	19.14063	132	92	0
0	1	46	1.75	30.04082	118	81	0
1	1	48	1.7	26.29758	166	107	0
0	1	65	1.75	24.4898	112	71	0
0	0	54	1.58	20.02884	117	82	0
0	1	73	1.7	27.68166	144	98	0
0	0	72	1.55	23.72529	138	81	0
1	1	38	1.8	20.37037	128	91	1
1	1	66	1.7	23.87543	160	105	0
1	0	60	1.66	21.04805	129	86	0
1	1	56	1.82	25.35925	189	115	1
0	1	23	1.8	31.48148	135	100	0
0	0	49	1.55	28.30385	138	95	0
0	1	78	1.73	25.05931	144	79	0
0	0	66	1.65	21.67126	106	73	0
0	1	28	1.83	28.36752	149	115	1

0	1	71	1.76	24.21229	154	92	0
0	0	50	1.53	26.05836	133	85	0
0	0	35	1.64	31.23141	109	81	0
0	1	37	1.75	32.65306	119	87	0
1	1	60	1.73	23.38869	141	102	0
0	1	67	1.72	28.39372	154	100	0
1	1	52	1.65	22.40588	167	67	0
0	1	54	1.72	25.68956	129	89	0
0	0	40	1.64	23.05176	150	95	0
0	1	42	1.7	29.06574	130	100	0
0	1	80	1.65	24.24242	119	77	0
1	0	65	1.61	21.98989	148	84	0
1	1	31	1.7	32.87197	164	110	0
1	1	58	1.72	25.01352	142	81	1
1	0	54	1.5	31.11111	164	100	0
1	0	60	1.57	20.69049	124	89	0
1	1	77	1.7	24.22145	149	84	0
0	1	51	1.65	22.40588	131	92	0
0	1	40	1.75	25.14286	132	98	1
0	1	70	1.7	25.77855	129	75	0
0	1	37	1.76	30.99174	133	103	1
1	1	41	1.71	31.46267	163	104	1
0	1	44	1.7	32.52595	126	87	1
1	0	53	1.55	23.72529	119	80	0
0	0	63	1.55	22.47659	158	88	0
0	1	52	1.76	28.08626	159	108	0
0	1	41	1.73	26.72993	112	89	1
0	1	56	1.75	22.20408	119	92	0
0	1	53	1.72	25.35154	157	108	0
0	1	33	1.7	29.23875	113	82	1
0	0	40	1.6	30.46875	143	95	0
1	1	53	1.75	27.7551	133	80	1
0	1	39	1.71	22.22906	128	81	0
1	0	66	1.64	20.07734	121	82	0
1	0	58	1.58	24.43518	143	91	0
0	1	64	1.7	22.49135	140	95	0
0	1	42	1.72	30.08383	137	98	0
0	0	24	1.65	23.87512	132	100	0
0	0	49	1.67	27.25089	145	101	0
0	1	60	1.68	23.03005	133	83	1
0	1	53	1.7	20.0692	127	100	1
0	1	49	1.75	22.20408	166	114	1
1	1	59	1.78	26.5118	109	80	0
0	1	53	1.75	29.71428	137	97	1
0	1	68	1.68	26.57313	158	96	1
0	1	45	1.78	24.6181	110	84	1

0	0	41	1.64	28.25699	142	84	0
1	0	66	1.55	21.22789	130	89	0
0	1	35	1.78	25.24934	114	78	1
0	1	53	1.7	27.16263	149	117	0
0	0	47	1.6	23.4375	129	100	0
0	0	67	1.52	29.43213	143	74	0
1	0	76	1.63	26.34649	171	100	0
0	0	74	1.5	22.22222	136	74	0
0	0	75	1.54	25.29938	172	79	0
0	1	44	1.66	32.29787	125	87	0
1	1	41	1.65	22.77319	136	100	0
0	1	75	1.73	28.73467	119	72	0
0	1	51	1.65	32.69054	154	106	0
1	0	54	1.59	30.65543	140	99	0
0	1	59	1.8	28.39506	128	93	1
1	1	44	1.83	20.90239	143	102	1
1	1	55	1.72	27.71768	148	90	0
1	0	72	1.55	33.29865	157	77	0
1	0	57	1.54	24.24524	155	110	0
1	0	52	1.6	24.21875	148	91	0
0	0	65	1.54	29.09428	134	73	0
0	1	50	1.8	25.92593	121	92	1
1	1	64	1.7	29.75778	149	98	0
1	1	75	1.76	22.59814	132	89	1
0	1	58	1.65	28.68014	127	96	0
0	1	36	1.82	30.18959	170	119	0
1	1	49	1.7	23.87543	132	74	1
1	1	61	1.75	24.16327	142	98	1
0	0	53	1.6	24.41406	156	95	0
0	0	78	1.55	28.72009	192	91	0
1	1	55	1.75	26.12245	160	108	0
0	1	62	1.76	19.36983	152	81	1
1	1	49	1.78	25.56495	127	91	0
1	1	41	1.7	29.41176	118	87	1
0	1	72	1.65	25.34435	134	92	1
0	1	65	1.63	27.09925	131	79	0
0	1	73	1.61	22.76147	171	88	1
0	0	69	1.52	27.26801	204	93	0
0	1	61	1.65	20.20202	150	84	0
1	0	67	1.58	26.43807	157	82	0
1	0	58	1.58	24.03461	162	78	0
0	0	36	1.62	28.57796	116	86	0
0	0	75	1.6	24.41406	171	88	0
1	0	55	1.63	21.0772	157	102	0
1	1	50	1.75	21.87755	104	73	1
0	0	56	1.59	28.08433	139	90	0

0	1	19	1.71	25.64892	137	91	0
1	0	58	1.62	30.48316	165	103	0
0	1	20	1.73	25.72755	130	94	0
1	1	46	1.74	26.42357	120	90	1
1	1	54	1.75	24.4898	146	102	0
0	0	40	1.55	22.06035	135	86	0
0	1	33	1.76	33.89721	139	82	0
0	1	42	1.78	22.7244	120	87	0
0	1	51	1.7	25.25951	135	98	0
0	0	74	1.55	28.72009	138	84	0
1	1	60	1.7	24.22145	135	90	0
0	0	61	1.62	24.76757	119	74	0
1	1	60	1.7	25.95156	181	102	0
0	0	61	1.53	25.63117	173	86	0
0	0	59	1.65	31.2213	137	88	0
1	0	59	1.6	25.39063	167	112	0
0	0	69	1.55	23.72529	168	87	0
1	1	46	1.64	27.14158	129	89	1
1	0	68	1.58	26.03749	153	76	0
1	1	46	1.69	22.05805	183	126	1
1	0	66	1.65	21.30395	151	74	0
0	1	51	1.73	26.06168	136	93	1
0	1	68	1.7	24.22145	126	76	1
1	1	77	1.7	26.6436	176	90	0
1	0	55	1.6	24.60938	153	94	0
0	1	54	1.7	26.98962	115	78	0
1	1	53	1.79	31.21001	157	90	1
1	0	54	1.6	30.85938	129	80	0
1	1	52	1.71	22.22906	127	90	1
0	0	56	1.62	25.14861	160	80	0
0	1	59	1.78	22.09317	128	84	1
0	1	61	1.7	31.14187	143	101	0
0	0	60	1.6	21.875	147	88	0
1	1	66	1.69	24.50894	173	79	0
1	1	60	1.73	22.72044	120	80	0
0	1	40	1.78	26.19619	120	84	1
1	0	59	1.62	22.10029	126	84	0
1	0	49	1.65	23.1405	176	95	0
0	1	58	1.76	25.18079	106	80	1
1	1	54	1.75	24.81633	152	102	0
1	1	39	1.74	26.75386	128	98	0
1	1	54	1.7	29.41176	132	93	1
1	0	54	1.63	29.35752	157	109	0
0	1	57	1.7	25.60554	132	91	1
1	0	70	1.52	23.8054	189	102	0
1	1	47	1.8	26.85185	117	81	0

0	1	84	1.7	31.14187	105	49	1
0	1	71	1.65	25.71166	148	84	1
1	1	59	1.7	24.22145	162	86	1
0	1	50	1.76	26.47211	138	98	0
0	0	54	1.58	19.62826	121	70	0
0	1	73	1.66	30.12048	109	70	1
1	1	65	1.67	25.0995	124	81	1
1	1	43	1.75	26.77551	143	100	0
0	0	66	1.57	21.09619	138	86	0
1	1	41	1.75	23.83673	127	95	0
0	0	59	1.6	22.7	140	88	0
0	0	55	1.59	21.43902	139	76	0
1	1	40	1.65	23.50781	149	105	0
0	1	53	1.78	26.354	141	96	1
0	0	37	1.5	28.88889	132	94	0
0	1	58	1.64	28.62879	142	86	1
1	1	54	1.75	22.85714	130	52	1
0	1	66	1.72	21.29529	106	70	1
0	1	44	1.73	25.05931	143	112	0
1	1	67	1.64	30.116	144	95	1
0	1	55	1.68	31.88776	156	89	0
0	1	38	1.7	20.76124	95	67	1
0	1	66	1.74	28.40534	144	96	0
0	1	51	1.8	24.69136	117	80	0
0	0	62	1.58	21.63115	137	82	0
1	0	58	1.67	25.0995	128	92	0
0	1	39	1.8	23.45679	116	92	0
0	1	61	1.73	23.38869	108	73	0
1	1	41	1.81	28.9979	127	86	1
0	0	33	1.65	17.63085	114	91	0
0	1	75	1.68	26.57313	134	74	1
0	1	49	1.7	30.79585	123	86	0
0	1	51	1.75	25.14286	170	110	1
1	1	69	1.6	24.21875	118	77	1
1	1	57	1.8	24.69136	163	96	1
0	1	56	1.72	29.06977	141	105	1
1	1	58	1.75	27.10204	134	84	1
0	0	52	1.57	28.39872	136	80	0
0	1	46	1.75	22.85714	132	99	1
1	1	57	1.71	21.2031	151	100	0
0	1	35	1.83	26.87449	142	101	1
1	0	58	1.58	26.03749	140	85	0
1	1	54	1.7	25.60554	139	91	0
0	1	37	1.76	29.70041	143	91	1
1	1	58	1.7	22.14533	160	90	1
1	1	70	1.7	19.03114	134	77	0

0	0	53	1.58	22.83288	158	87	0
0	0	60	1.46	25.80221	128	81	0
0	1	70	1.8	24.07408	139	89	0
1	1	54	1.77	27.45061	142	101	1
0	1	47	1.8	25.30864	151	90	0
0	0	54	1.62	20.95717	111	75	0
1	0	45	1.58	24.03461	125	82	0
1	1	62	1.57	22.71897	124	83	0
1	1	64	1.7	20.41522	134	81	0
0	0	69	1.62	24.76757	150	72	0
0	0	62	1.58	21.63115	112	74	0
0	1	43	1.7	25.0865	132	98	0
1	0	69	1.55	31.21748	153	77	0
0	1	53	1.73	22.38631	122	88	0
1	0	66	1.58	30.44384	155	90	0
0	0	67	1.6	23.4375	118	79	0
0	1	66	1.75	21.22449	142	89	1
1	1	49	1.74	21.46915	151	100	0
1	1	61	1.75	23.18367	137	79	1
1	1	69	1.76	23.56663	158	96	1
0	0	68	1.58	23.23346	171	88	0
0	0	18	1.54	20.66116	131	90	0
1	0	50	1.68	23.73866	148	104	0
1	0	71	1.55	24.55775	107	82	0
0	1	60	1.75	22.53061	125	93	1
1	1	33	1.75	20.57143	107	82	0
1	0	54	1.6	30.46875	144	90	0
0	0	54	1.52	24.23823	135	88	0
1	0	55	1.7	23.18339	115	75	0
1	1	51	1.83	23.88844	98	67	0
1	0	63	1.57	30.0215	128	77	0
0	0	59	1.6	24.21875	159	95	0
0	1	33	1.78	29.98359	134	99	0
0	0	60	1.63	22.95909	167	104	0
1	1	70	1.73	26.06168	130	90	1
1	1	45	1.71	29.06877	124	79	0
0	1	58	1.67	22.58955	125	87	1
0	1	64	1.7	26.6436	144	94	1
1	0	56	1.55	24.97399	130	86	0
0	1	66	1.61	27.00513	133	91	1
0	0	25	1.6	27.34375	116	86	0
1	1	40	1.75	22.53061	116	87	1
0	1	51	1.7	31.14187	130	93	0
1	1	61	1.72	23.66144	149	91	0
0	1	78	1.7	24.22145	137	76	0
0	1	41	1.75	32.97959	160	106	0

1	1	64	1.7	24.22145	175	99	1
0	0	69	1.65	33.05785	168	92	0
0	1	53	1.81	24.41928	162	108	1
1	1	66	1.75	26.12245	125	77	1
0	1	44	1.76	22.59814	143	94	1
0	0	61	1.56	22.60027	140	91	0
1	1	54	1.65	23.1405	160	79	1
0	0	60	1.6	25.39063	165	89	0
0	1	55	1.67	30.47797	133	89	1
0	1	60	1.7	26.29758	142	80	0
0	1	22	1.85	29.21841	141	99	1
0	1	42	1.76	29.70041	131	91	1
0	1	39	1.75	26.77551	131	93	0
0	1	66	1.7	23.18339	141	82	0
1	0	49	1.6	36.71875	120	80	0
1	1	66	1.7	23.3564	120	68	0
0	1	62	1.72	26.70362	121	96	0
0	1	51	1.7	25.95156	129	89	0
0	1	37	1.71	32.48863	151	108	1
1	1	59	1.7	24.91349	153	87	1
1	0	69	1.62	28.19692	163	90	0
0	1	43	1.7	25.60554	118	78	1
1	1	50	1.7	24.91349	157	83	0
0	0	42	1.64	21.93635	112	86	0
1	0	65	1.61	23.22441	143	64	0
1	1	60	1.7	21.79931	107	75	1
1	0	47	1.58	20.82999	159	109	0
0	1	66	1.72	25.01352	137	78	0
0	1	64	1.8	23.58025	148	102	1
0	1	74	1.76	27.11777	124	79	1
0	0	60	1.51	24.9989	123	82	0
1	1	66	1.65	24.24242	179	91	0
0	0	58	1.6	28.51563	177	105	0
0	1	70	1.8	24.69136	129	86	0
1	1	66	1.8	30.8642	144	86	0
0	0	55	1.55	18.73049	125	73	0
0	1	23	1.7	27.16263	133	95	0
1	1	44	1.65	29.38476	141	95	0
1	0	52	1.62	23.62445	125	91	0
0	0	61	1.62	34.29355	133	88	0
1	1	54	1.6	23.4375	155	90	0
0	1	52	1.74	24.7721	121	80	0
0	1	73	1.68	26.04167	126	76	0
0	1	60	1.7	25.95156	125	89	0
1	0	73	1.58	22.03172	119	81	0
0	0	67	1.55	24.97399	143	81	0

0	0	50	1.56	22.60027	139	96	0
0	1	42	1.86	34.39704	124	99	0
1	0	61	1.6	25.39063	143	99	0
1	1	40	1.78	21.30413	113	86	1
0	1	48	1.76	31.6374	155	109	0
0	1	59	1.75	25.79592	125	85	0
0	1	26	1.75	40.4898	131	78	1
0	1	73	1.68	26.04167	126	76	0
0	1	43	1.78	27.14304	130	103	0
0	1	42	1.76	25.18079	120	91	0
0	1	49	1.6	26.95313	144	94	0
1	0	65	1.6	29.29688	157	97	0
0	1	60	1.64	26.76978	139	89	0
1	0	60	1.62	23.62445	159	108	0
0	0	67	1.65	29.38476	130	70	0
1	1	73	1.65	26.81359	157	81	1
0	1	62	1.77	28.40818	105	83	0
0	0	60	1.65	26.44628	150	110	0
0	0	54	1.6	29.29688	125	97	0
1	0	68	1.68	25.68736	144	77	0
1	1	58	1.63	24.53988	117	72	0
1	1	78	1.7	21.6263	133	86	0
0	1	58	1.73	22.38631	135	92	0
0	1	56	1.78	27.77427	133	90	0
1	1	43	1.75	22.85714	109	78	0
0	1	68	1.65	23.87512	145	84	1
0	1	36	1.8	25.92593	134	100	1
0	1	67	1.76	28.08626	155	87	1
0	1	82	1.6	25.39063	165	96	0
0	0	53	1.6	26.95313	129	96	0
1	1	52	1.7	26.29758	128	91	0
1	0	62	1.54	34.99747	179	102	0
1	0	46	1.6	23.4375	165	116	0
0	0	71	1.55	27.05515	186	87	0
0	1	50	1.76	29.37758	106	83	1
0	1	67	1.6	29.29688	144	71	0
0	0	45	1.64	24.72487	141	97	0
1	1	46	1.74	38.97477	152	104	0
0	1	36	1.75	20.40816	123	81	1
0	1	51	1.65	25.34435	125	100	1
0	0	52	1.58	24.03461	166	106	0
0	0	55	1.58	20.42942	128	89	0
1	0	59	1.6	31.26	154	94	0
0	0	49	1.6	29.29688	160	90	0
0	0	62	1.53	26.91273	125	71	0
1	1	55	1.75	26.12245	156	96	0

0	1	53	1.77	23.93948	116	78	0
0	1	44	1.74	25.10239	125	90	0
0	0	55	1.72	23.66144	157	79	0
1	1	27	1.78	25.88057	140	96	0
0	1	41	1.8	27.77778	124	81	0
0	0	75	1.58	28.04038	130	78	0
0	1	66	1.72	23.66144	116	86	1
1	0	61	1.48	23.96822	148	99	0
1	0	57	1.6	19.92188	107	81	0
1	0	58	1.56	24.65483	140	93	0
1	0	48	1.55	33.29865	133	98	0
0	1	47	1.85	24.25128	128	89	1
1	1	63	1.72	22.9854	140	95	0
1	0	71	1.6	25.39063	135	85	0
1	1	73	1.7	22.14533	147	80	1
1	1	56	1.7	29.41176	124	87	0
0	0	65	1.58	23.23346	178	98	0
0	1	42	1.7	29.41176	124	87	0
0	1	68	1.74	27.74475	121	80	0
0	0	63	1.62	19.43301	111	70	0
0	1	42	1.78	28.40551	120	94	0

Drink	FH	HBH	COD	intervention	PN	NV	BVP
0	0	1	120	1	1	1	1
0	0	1	336	1	1	1	1
1	1	0	132	1	1	1	1
1	0	1	120	1	1	1	1
0	0	0	108	1	1	1	1
0	0	0	24	1	1	0	0
0	0	1	84	1	1	1	1
0	0	1	60	1	1	1	1
0	1	1	144	1	1	1	1
1	0	0	180	1	1	1	1
1	0	1	0.5	0	1	0	0
0	0	0	12	1	1	1	0
1	0	0	24	0	1	0	0
0	0	0	0.5	0	1	1	0
0	1	1	192	1	1	0	1
0	1	0	24	1	1	0	1
1	0	0	1	1	1	1	1
0	0	0	0.5	1	1	1	1
0	0	1	240	1	1	1	1
0	1	0	60	1	1	1	1
0	1	1	144	1	1	0	0
1	1	0	228	1	1	1	1
0	0	1	168	1	1	0	0
0	0	0	24	1	1	0	0
0	0	0	180	1	1	1	1
1	0	0	48	1	1	0	1
0	1	0	288	1	1	1	1
0	0	1	240	1	1	0	1
0	0	0	120	1	1	1	0
0	0	0	192	1	1	0	0
0	1	0	120	0	1	0	1
0	0	1	36	1	1	0	0
0	1	0	132	1	1	0	1
0	0	0	60	1	1	0	1
1	0	1	336	1	1	1	1
0	0	1	84	1	1	1	1
1	0	1	156	1	1	1	1
1	1	1	312	1	1	1	1
0	1	1	180	1	1	0	1
1	0	1	48	0	1	1	1
1	0	0	6	0	1	0	0
0	0	0	42	1	1	0	1
0	0	0	312	1	1	0	0
0	1	0	192	1	1	1	0
0	0	1	24	1	1	0	0

0	0	1	240	1	1	1	0
0	0	1	2	1	1	1	1
0	0	0	6	0	1	0	0
0	1	0	48	1	1	0	0
0	1	0	120	1	1	1	1
0	0	1	120	1	1	0	0
1	0	0	24	1	1	1	1
0	0	0	240	1	1	0	1
0	1	0	3	0	1	0	0
0	1	0	0.5	0	1	0	0
0	1	1	276	1	1	1	1
0	1	0	216	1	1	1	1
0	0	1	24	1	1	1	1
0	1	1	240	1	1	1	1
0	0	0	72	1	1	1	1
0	0	0	8	0	0	1	1
0	1	1	360	1	1	1	1
0	1	0	96	1	1	0	1
1	0	1	0.5	0	0	0	0
0	0	0	24	1	1	1	1
1	0	0	2	0	1	1	1
1	0	0	120	1	1	1	1
1	0	0	0.5	1	1	0	1
0	1	0	2	0	1	0	0
0	0	0	180	1	1	1	1
0	0	0	6	1	1	1	1
1	1	0	132	1	1	0	0
0	1	0	3	0	1	0	1
0	1	1	132	1	1	0	1
1	1	0	6	0	0	0	0
0	1	0	12	0	1	0	0
1	1	1	168	1	1	1	1
0	0	0	6	0	1	0	0
0	1	0	24	1	1	1	1
0	0	1	216	1	1	1	1
0	0	1	252	1	1	1	1
0	1	0	120	1	1	1	1
0	0	0	24	0	1	1	0
0	1	0	12	0	0	1	1
1	1	1	120	1	1	1	1
0	0	0	6	1	1	1	1
1	0	1	1	0	0	1	0
0	1	1	84	1	1	1	1
1	1	0	0.5	0	1	1	1
0	0	1	216	1	1	1	1
1	1	0	156	1	1	0	0

0	1	0	96	1	1	0	1
0	0	1	120	1	1	1	1
1	0	0	24	0	1	0	0
1	1	1	36	1	0	1	1
0	1	0	120	1	1	0	0
0	0	1	168	1	1	0	1
0	0	1	84	1	1	1	1
0	0	0	216	1	1	1	1
0	0	1	300	1	1	1	1
1	1	1	120	1	1	0	0
0	1	0	24	0	1	0	0
0	0	0	156	1	1	1	1
0	0	1	0.5	1	1	0	1
0	1	1	12	1	1	1	1
1	1	1	240	1	1	1	1
0	0	0	120	0	1	0	0
1	1	1	156	1	1	1	1
0	1	0	276	1	1	1	1
0	0	1	36	1	1	0	1
0	1	0	96	1	1	1	1
0	0	0	300	1	1	1	1
1	0	1	12	1	1	1	1
1	0	1	132	1	1	1	1
0	1	0	240	1	1	1	1
1	1	0	36	0	0	0	1
0	0	0	78	1	1	1	0
1	1	1	36	1	1	1	1
1	0	1	120	1	1	1	1
0	1	0	120	1	1	0	0
0	1	1	492	1	1	1	1
0	1	1	276	1	1	0	1
0	0	0	0.5	0	1	1	1
0	0	0	96	1	1	0	1
1	0	0	108	1	1	0	1
1	0	0	1	1	1	1	1
0	1	1	132	1	1	0	1
0	0	1	96	1	1	1	1
0	0	1	48	1	1	1	1
0	0	1	60	1	1	1	1
0	1	1	348	1	1	1	1
0	0	1	300	1	1	1	1
0	1	0	6	1	0	0	0
0	0	0	240	1	1	1	1
0	1	1	120	1	1	1	1
1	1	0	132	1	1	1	1
0	1	1	60	1	1	0	0

0	0	0	12	1	1	0	0
0	0	1	240	1	1	1	1
1	1	0	2	1	1	0	0
0	0	0	96	1	1	1	1
0	0	1	168	1	1	1	0
0	0	0	0.5	0	1	1	0
1	0	0	24	1	1	0	0
1	0	0	132	1	1	1	0
0	1	1	72	1	1	1	1
0	1	1	228	1	1	1	1
1	1	0	84	1	1	0	0
0	1	0	204	1	1	1	0
0	1	1	228	1	1	1	1
0	0	0	120	1	1	1	1
0	0	1	84	1	1	1	1
0	0	1	96	1	1	1	0
0	0	1	240	1	1	1	1
0	1	0	120	1	1	0	0
0	1	1	180	1	1	1	1
1	1	1	72	1	1	1	1
0	0	0	120	1	1	1	1
1	1	1	36	1	1	0	0
1	1	0	144	1	1	0	0
0	1	0	120	1	1	0	1
0	1	1	168	1	1	1	1
0	1	0	36	1	1	1	1
1	0	1	192	1	1	0	0
0	0	0	228	1	1	1	1
1	0	1	108	1	1	1	1
0	1	0	24	1	0	1	1
1	0	0	276	1	1	1	1
1	0	1	84	1	1	1	1
0	0	0	120	1	1	1	1
0	0	1	120	1	1	0	1
1	1	0	360	1	0	0	1
1	1	0	84	1	1	1	0
0	1	0	84	1	1	1	1
0	1	1	120	1	1	0	1
1	0	0	72	1	1	1	1
0	0	1	120	1	1	1	1
0	0	0	1	1	1	0	0
1	0	1	156	1	1	1	1
0	0	1	96	1	1	0	0
0	1	0	96	1	1	1	1
0	1	1	300	1	1	1	1
1	0	1	48	1	1	1	1

1	0	1	60	1	1	1	1
0	0	0	240	1	1	1	1
1	1	0	36	1	1	1	1
1	0	1	48	1	1	1	1
0	0	0	120	1	1	0	0
1	0	0	216	1	1	1	1
1	0	1	156	1	1	1	1
0	1	1	36	1	1	1	0
0	0	1	120	1	1	1	1
0	1	1	144	1	1	1	1
0	0	1	108	1	1	1	1
0	1	1	72	1	1	1	1
0	0	0	48	1	1	1	1
1	0	0	24	1	1	0	1
0	0	1	144	1	1	0	0
1	1	0	72	1	1	1	1
1	0	1	240	1	1	1	1
0	0	1	360	1	1	1	1
1	1	0	0.5	0	1	1	1
1	0	0	60	1	1	1	0
0	0	1	60	1	1	0	1
1	0	0	24	0	1	0	0
1	1	1	12	0	1	1	1
0	1	0	12	1	1	1	1
0	0	1	84	1	1	0	1
0	0	1	72	1	1	0	0
0	0	0	60	1	1	0	0
0	0	0	84	1	1	1	0
1	1	0	120	1	1	1	1
0	0	0	36	1	1	0	0
1	1	1	96	1	1	1	1
0	0	0	108	1	1	0	0
1	1	1	108	1	1	1	1
1	1	0	240	1	1	1	1
1	0	1	72	1	1	1	1
1	0	0	120	1	1	0	0
0	0	1	84	1	1	1	1
0	1	1	24	1	1	1	0
1	1	0	18	0	1	0	1
0	1	1	96	1	1	1	1
1	0	0	1	0	1	1	1
0	1	1	2	0	1	1	1
0	0	0	252	1	1	1	1
0	0	1	48	1	1	0	0
0	1	1	252	1	1	1	1
0	1	1	240	1	1	1	1

0	1	0	36	1	1	1	0
0	1	0	0.5	0	0	1	1
0	0	0	120	1	1	1	1
1	0	0	156	1	1	1	1
1	1	0	84	1	1	0	1
0	0	0	48	1	1	1	1
0	0	1	228	1	1	0	0
1	1	1	24	1	1	1	1
0	1	1	240	1	1	1	1
0	0	1	96	1	1	1	1
0	1	0	312	1	1	1	0
1	1	0	60	1	1	0	0
0	0	1	96	1	1	1	1
1	1	1	252	1	1	1	1
0	1	0	120	1	1	1	1
0	1	1	84	1	1	1	1
0	0	0	240	1	1	1	1
1	0	0	96	1	1	1	1
0	1	0	240	1	1	1	0
0	1	1	360	1	1	1	1
0	1	1	192	1	0	1	1
0	1	0	12	0	0	0	0
0	1	0	120	1	1	1	1
0	0	0	120	1	0	1	1
1	0	1	120	1	0	1	1
1	1	0	72	1	1	1	1
0	0	1	36	0	1	0	1
0	0	0	120	1	1	1	0
0	1	0	180	1	1	1	1
1	1	0	120	1	0	0	1
0	0	1	312	1	1	1	1
0	1	1	120	1	1	1	1
1	1	1	0.5	1	1	0	0
0	1	1	84	1	1	1	1
0	1	0	300	1	1	1	1
1	0	0	84	1	1	0	0
1	0	0	120	1	1	1	1
0	0	0	4	0	1	1	1
0	1	1	156	1	1	1	0
0	1	0	72	1	1	1	1
0	1	0	6	0	1	0	0
1	0	0	24	0	1	0	0
0	0	1	36	1	1	1	1
0	0	0	72	1	1	1	1
0	0	1	36	1	1	1	1
1	1	0	24	1	1	1	0

0	0	0	120	1	1	1	1
0	0	1	120	1	1	0	1
1	0	0	192	1	1	0	1
1	0	1	252	1	1	1	1
1	0	0	144	1	0	1	0
0	1	0	72	1	1	0	1
1	0	0	60	1	1	1	0
0	1	1	132	1	1	1	0
1	1	1	288	1	1	0	1
1	0	1	8	0	1	0	0
1	1	0	12	1	1	0	0
1	0	0	5	1	0	0	0
0	1	0	96	1	1	0	0
0	1	0	288	1	1	0	1
0	0	0	120	1	1	1	0
0	1	0	252	1	1	1	1
1	1	0	60	1	1	1	1
1	1	0	204	1	1	1	0
1	0	0	60	0	1	1	0
1	1	1	204	1	1	0	0
0	1	1	180	1	1	1	1
1	0	0	24	1	1	0	1
1	0	0	300	1	1	1	1
1	0	0	12	1	1	0	0
0	0	0	132	1	1	1	1
1	1	0	72	1	1	1	1
0	0	1	120	1	1	0	0
0	0	1	252	1	1	1	1
1	0	1	240	1	1	1	1
0	0	0	120	1	1	1	1
0	0	0	204	1	1	1	1
0	1	0	180	1	1	1	1
0	0	1	60	1	1	1	1
1	0	0	72	1	1	1	1
1	1	1	312	1	1	0	1
0	0	0	132	1	1	0	0
0	0	0	6	1	1	0	0
0	0	0	72	1	1	1	1
0	0	1	120	1	1	1	0
0	0	0	228	1	1	1	0
0	1	0	108	1	1	1	1
0	0	0	216	1	1	1	1
0	0	1	72	1	1	1	1
0	0	0	84	1	1	1	0
0	0	0	48	1	1	0	1
0	0	0	168	1	1	1	1

0	1	0	84	1	1	1	1
0	0	1	0.5	0	1	0	0
0	1	0	144	1	1	1	1
1	1	0	0.5	0	1	1	1
1	0	1	144	1	1	1	0
0	0	0	240	1	1	1	1
0	1	0	12	1	0	0	0
0	0	1	72	1	1	1	1
0	1	1	1	0	1	1	0
0	1	0	1	0	1	0	1
0	0	0	60	1	1	1	1
0	0	1	144	1	1	0	1
1	1	1	168	1	1	1	1
0	1	1	240	1	1	1	1
0	0	1	48	1	1	1	1
0	1	1	96	1	1	1	1
1	0	1	180	1	1	0	1
0	1	1	120	1	1	1	1
0	0	0	24	1	1	1	1
0	0	1	228	1	1	1	1
0	1	0	240	1	1	0	1
0	0	1	132	1	1	1	1
0	0	1	60	1	1	0	0
1	0	1	84	1	1	1	1
0	0	0	96	1	0	1	1
1	0	1	240	1	1	1	1
0	1	0	48	1	1	1	1
0	1	1	120	1	1	1	1
1	0	0	336	1	1	1	1
0	1	0	12	1	1	0	0
0	0	0	228	1	1	1	0
0	0	1	132	1	1	1	1
0	0	1	2	1	1	0	0
0	0	1	276	1	1	1	1
1	0	1	3	0	1	1	0
0	0	0	240	1	1	1	1
0	0	0	36	0	0	1	0
0	1	0	6	1	1	1	1
1	1	0	24	1	1	0	0
0	0	1	96	1	1	1	1
0	1	1	0.5	0	1	1	1
0	1	0	36	1	1	1	1
0	1	1	60	1	1	1	1
0	0	1	72	1	1	1	1
0	0	0	120	1	1	1	1
1	0	1	216	1	1	1	1

0	1	1	192	1	1	1	1
0	1	0	84	1	1	0	1
0	1	0	180	1	1	0	1
0	0	1	24	1	1	0	0
0	0	0	48	1	1	1	0
0	0	1	72	1	1	1	1
0	1	0	180	1	1	1	1
0	0	1	240	1	1	1	1
0	1	0	120	1	1	1	0
0	1	0	120	1	1	1	1
0	1	1	72	1	1	1	1
1	0	0	72	1	1	1	1
0	0	0	144	1	1	0	1
0	1	1	240	1	1	1	1
0	1	1	120	1	1	1	1
0	0	0	60	1	1	1	1
0	0	1	192	1	1	1	1
0	0	0	96	1	1	0	0
1	1	1	0.09	0	1	1	1
0	1	0	1	0	1	0	1
0	1	1	36	1	1	1	0

LEVD	HOMA-IR	TyG	WBC	RBC	HB	PLT	FIB
1	0.58213333	2.89238362	5.13	5.44	162	190	2.84
1	2.30445333	2.71823813	5.83	4.58	129	254	3.01
1	4.1678	2.65570372	6.23	4.8	154	214	3.04
1	2.43754222	2.31629074	9.25	5.19	155	340	3.18
1	3.93604444	1.44206014	6.78	4.3	138	226	2.32
1	4.15813333	1.22898244	5.42	4.29	94	334	3.04
1	0.27655556	1.82299971	5.83	4.79	133	138	3.4
1	2.23141333	1.28018356	5.66	4.89	131	174	4.03
1	3.89993333	2.25461259	6.08	3.91	116	191	2.89
1	2.78952889	2.39781345	8.02	5.12	150	328	2.7
1	1.81534667	3.65138784	8.41	5.47	161	206	2.35
1	0.18292889	0.9253694	5.63	5.35	162	221	3.21
1	5.72324	2.55691719	7.13	5.71	169	155	2.89
1	3.57964444	1.98499349	6.82	5.5	178	203	2.8
1	1.93555556	2.79277424	8.26	4.59	136	251	3.93
1	2.57724444	2.62880495	6.42	5.58	164	200	2.75
1	0.77272	2.44850197	5.37	4.72	142	328	2.51
1	15.3336089	1.5832582	5.15	5.05	148	205	3.46
1	0.6288	1.49656032	6.28	4.63	138	203	3.04
1	2.46202667	2.59936565	5.73	6.06	155	308	2.05
1	11.60464	2.00767697	5.4	5.18	163	261	2.46
1	2.46164	4.3518058	5.52	4.86	145	183	3.26
1	3.91619556	3.0566839	7.45	4.99	155	192	2.89
0	2.112	1.48225206	4.93	5.13	150	185	2.09
1	7.02764444	1.83940612	6.2	4.92	147	174	3.04
1	2.39835556	1.04721373	5.65	5.15	170	301	2.99
1	0.18149333	2.0612903	6.24	5.6	110	335	2.89
1	8.62378667	0.90194837	8.62	3.64	108	235	3.52
1	4.44901778	2.13198655	7.99	4.45	126	193	4.79
1	1.85205333	1.47814402	5.11	4.57	139	266	3.04
1	2.96760444	2.23235038	5.14	4.78	143	183	2.46
1	2.29856	1.07329448	5.26	5.04	154	156	1.97
1	2.48658667	1.8434027	4.63	4.76	143	209	2.55
1	1.12782667	2.38329835	4.22	4.45	137	143	2.55
1	6.04139556	2.0606027	4.45	4.64	153	194	2.15
1	0.21618222	2.1861861	8.21	4.59	137	274	3.2
1	2.56652	1.15322673	5.72	4.78	150	128	2.19
1	19.67504	4.78813112	6.86	4.45	152	213	3.09
1	11.1971422	1.04503569	6.08	3.75	118	249	3.2
1	1.66730667	2.88242639	3.84	5.06	163	203	2.59
0	1.38271111	1.13327133	8.46	6.18	186	210	2.29
1	1.98439111	2.61828595	4.91	4.58	133	304	2.15
1	3.77955111	2.80313308	5.01	5.47	167	142	2.22
1	3.13384	2.8109527	5.45	4.73	133	171	2.29
1	3.91586667	3.55769103	9.06	5.85	175	233	2.22

1	5.06032889	1.28259912	6.58	4.67	147	167	1.71
1	0.60946222	4.11120823	5.24	4.58	131	201	5.79
0	3.52026667	1.5271861	4.75	5.28	113	223	3.09
1	5.57488444	2.92153122	6.04	5.52	153	242	2.21
1	0.74277333	1.54698788	6.53	4.98	168	185	2.19
1	4.16543556	0.25359516	6.26	4.84	151	189	2.79
1	3.16342222	2.03441149	4.76	4.24	129	273	2.94
1	4.47792	4.23942567	7.17	5.1	162	265	2.55
1	12.6208	3.49526133	8.12	4.92	143	272	4.79
1	2.44719556	3.54223387	7.66	5.78	181	239	2.46
1	4.73645333	2.04735085	7.43	4.46	138	151	2.35
1	0.24131556	1.84712322	4.38	4.44	131	192	2.75
1	3.78858667	1.36863943	4.61	4.82	133	172	1.95
1	2.17006667	0.96416619	9.84	4.22	135	277	3.61
1	2.86124	2.66460672	5.42	4.18	131	280	2.71
0	0.94653333	2.41686422	6.81	4.93	143	346	3.21
1	5.35936	2.89281876	6.08	4.12	126	193	2.89
1	2.07150667	1.98341235	6.82	5.24	154	203	1.95
0	6.91811111	2.528026	7.53	5.23	164	141	2.01
1	3.49738222	0.6672138	5.17	4.05	137	206	2.95
1	1.21322667	3.93263413	5.82	5.48	174	188	2.54
1	1.71872	4.57833687	5.36	4.92	146	250	3.61
1	1.97831111	4.68475741	7.9	5.16	155	264	4.12
1	0.5	1.30367333	4.51	4.89	146	212	2.47
1	0.4498	2.00826772	5.18	4.31	132	267	2.84
0	0.2618	2.90952058	4.83	4.43	148	150	3.46
1	5.41591111	3.13824044	6.14	5.86	175	234	3.45
1	0.74130222	3.70959466	5.1	5.29	171	234	3.15
1	9.53808	4.00729682	4.88	5.68	166	177	3.01
0	2.24898667	2.38349665	8.33	4.98	161	263	2.75
1	3.71944444	2.10478029	4.9	4.8	135	246	2.79
1	20.7454667	1.28460764	6.92	4.73	149	202	3.53
1	0.21865333	2.00968945	5.24	4.1	124	225	2.71
1	1.89926667	1.76591161	5.56	4.07	119	297	3.18
1	0.3078	2.72057148	5.49	4.56	138	214	2.7
1	3.68631111	0.56860385	5.61	4.24	128	159	2.79
1	4.34096889	2.84017435	6.84	5.38	173	150	2.99
1	1.49414222	2.52972067	7.01	5.16	150	304	4.33
0	1.03387556	2.5477719	5.8	4.81	146	366	3.27
1	1.17574222	0.98998719	6.49	4.35	135	240	2.99
1	1.84212	2.67287888	7.72	4.87	150	431	5.08
0	1.18249333	3.06364323	7.04	5.18	158	222	2.12
1	4.30106667	3.00090637	8.22	5.34	155	211	2.33
1	3.67797333	3.26141411	10.55	5.41	162	294	3.15
1	1.41884444	2.40287377	10.58	4.85	142	264	3.92
1	7.61066222	3.24569301	4.35	5.24	154	177	2.12

1	5.55082222	2.21393965	6.71	5.1	143	200	3.09
1	2.11352	2.81145781	7.23	3.98	112	229	2.58
0	2.00777778	3.06835754	5.55	5.52	162	234	2.35
1	3.92448	2.07518249	6.02	4.76	143	251	2
1	0.50094222	2.17375122	7.6	5.02	151	252	2.7
1	4.88558222	2.78678744	5.25	4.44	136	207	3.21
1	0.56848	2.32571551	6.43	4.03	116	177	4.14
1	4.39392	1.29978286	5.75	3.87	103	247	4.38
1	2.45559111	2.49618036	5.96	4.18	116	228	2.62
1	1.61532444	3.34660567	5.74	5.11	156	183	2.89
1	11.9588933	1.36825767	4.73	4.65	152	185	2.12
1	3.74498667	3.19938715	7.8	4.81	133	256	6.29
1	2.01157778	2.80572123	7.18	4.98	155	299	2.66
1	5.34296	2.794552	8.02	5.04	158	285	4.03
1	6.01352889	1.26624313	6.92	5.08	168	324	3.43
1	0.41979556	2.7541956	9.86	5.01	151	264	3.09
1	2.54349333	2.79452449	7.28	4.67	146	186	2.3
1	1.91173333	3.11266161	8.98	4.36	132	191	2.89
1	5.26464	1.66559138	7.18	4.82	145	262	2.66
1	1.76906667	2.02864812	6.66	3.82	115	205	1.87
1	2.7258	1.21697294	6.4	4.49	133	198	2.79
1	1.21345778	2.12241123	4.42	5.18	160	220	3.38
1	2.61433333	2.60863494	7.44	4.83	150	291	4.03
1	1.26933333	1.79997562	9.41	5.09	142	242	3.15
1	7.27484444	1.88056368	5.6	4.25	138	246	2.94
1	3.47619556	1.52127314	5.5	6.43	177	251	2.42
1	4.10526667	2.06254967	5.92	4.54	141	129	2.54
1	0.19253333	2.6996954	5.69	4.87	148	184	2.94
1	0.38487556	1.657828	3.27	4.58	141	338	2.1
1	3.20346667	1.38077918	4.67	4.05	136	176	3.18
1	1.54695111	2.75346324	4.77	4.84	156	224	2.62
1	0.74796	1.31185364	5.78	5.25	140	226	1.91
1	8.53733333	1.9058914	7.84	5.46	169	210	2
1	1.76845778	3.17074384	6.29	5.43	172	236	2.75
1	2.92082667	3.00716665	6.52	4.59	144	253	5.4
1	14.6857244	1.4307896	4	4.81	141	222	2.5
1	0.53037778	1.44055762	5.84	4.25	122	258	2.25
1	0.47226667	2.09204923	7.35	5.01	135	177	3.76
1	3.78922667	2.64098048	7.8	5.05	156	227	3.23
1	2.92876	1.95706202	5.84	4.05	126	182	3.02
1	2.97372444	1.75943426	6.69	3.44	102	224	3.45
0	5.68784	1.45167237	4.95	4.84	141	231	2.17
1	1.41488	2.52160013	7.44	4.23	129	349	2.66
1	1.52692	2.91490176	3.42	4.44	127	127	4.95
1	1.09956	2.38232006	5.13	4.51	142	229	2.79
1	7.25386667	1.78231501	7.28	4.49	136	190	4.03

1	4.41041778	3.53229143	6.13	6.34	163	255	4.5
1	0.80469333	3.53457814	7.1	4.29	131	204	4.14
1	1.38805333	3.11563306	9.59	5.21	155	265	2.46
1	6.54736	2.91346418	10.68	5.33	171	286	2.97
1	2.31048889	1.8955788	6.2	5.43	163	228	3.12
1	2.13853333	1.52187366	7.15	4.33	101	379	3.21
1	3.65432889	2.08181373	7.37	5.21	147	206	2.75
1	4.95106667	1.97487932	5.3	5.22	158	195	3.01
1	2.05994667	2.60501661	6.3	6.2	179	188	2.32
1	3.97079111	2.33361854	6.96	4.57	132	291	3.53
1	1.91281778	2.31861591	6.43	4.47	143	220	2.75
1	3.94142222	1.67134172	5.49	4.24	130	263	2.21
1	17.6170622	4.08547871	5.43	4.2	126	307	3.33
1	2.22288	1.75716802	7.15	4.46	134	313	2.32
1	3.82293333	2.01701412	6.51	4.04	130	242	3.26
0	2.34476	2.25453915	4.59	4.13	122	190	2.89
1	0.6688	1.03864995	5.46	3.55	116	210	2.51
1	2.72384	1.53579137	12.99	5.14	158	240	2.62
1	1.40144	0.81591776	6.39	3.77	123	210	2.46
1	1.52793333	2.75894608	8.09	5.31	165	240	2.94
1	1.50222222	1.86698289	5.41	4.04	135	139	2.79
1	5.65486222	1.72318615	8.13	5.03	152	282	2.62
1	0.24768	2.18626474	5.08	5.28	169	211	2.99
1	1.23804	1.17825492	6.86	3.13	99	149	3.04
1	1.38869333	3.61344849	5.72	4.62	140	96	2.39
1	9.01493778	4.01682887	5.14	4.91	150	150	2.7
1	6.76251111	1.17037466	6.61	5.49	175	264	2.84
1	7.74428889	1.55483388	5.82	4.57	126	214	5.08
1	1.95315556	1.78455861	6.57	4.79	145	243	2.58
1	2.29632	1.84422541	4.71	4.65	135	248	2.95
1	4.2112	3.41200543	4.78	4.98	143	240	2.24
1	3.78552889	2.63852147	7.03	4.91	156	174	2.69
1	5.84624444	2.71777278	5.62	4.38	127	184	2.29
1	2.38957333	1.81881514	4.55	4.51	145	320	2.8
1	1.19057778	2.66235175	3.75	5.15	145	157	2.43
1	2.86092	1.88071618	12.34	5.36	159	207	5.4
1	3.60997778	2.53347854	4.92	4.45	132	98	4.03
1	8.56014222	4.14505352	6.93	4.44	134	374	2.47
1	1.67872	3.2766616	5.94	6.46	164	106	2.95
1	2.81234667	2.85246218	5.64	5.67	174	218	2.66
0	7.33027556	2.39421122	5.51	5.63	158	236	2.46
1	14.70528	2.5121894	6.22	5.46	171	179	2.54
1	2.64677778	2.35678915	7.09	4.82	149	217	3.09
1	2.13442222	3.87351708	5.18	4.96	154	164	2.13
1	2.24398222	1.59568376	5.72	3.89	113	292	2.89
1	1.88336	2.88322405	6.69	4.55	139	265	2.62

1	5.0692	4.40010105	7.27	4.23	135	163	3.61
1	0.82562222	2.91418594	7.11	4.84	153	207	2.62
1	2.16414222	1.82554879	6.03	4.22	135	165	3.46
1	1.50798667	2.23471302	8.13	5.57	169	198	4.77
1	0.06983111	0.06952606	4.63	3.87	121	161	2.54
1	20.16112	1.60186934	6.74	4.82	145	194	3.53
1	7.39918222	0.86970214	7.35	4.93	150	321	4.26
1	1.56416	3.96327775	5.85	5.69	162	290	3.25
1	0.43605333	2.04987111	6.62	4.11	131	201	3.04
1	1.2768	2.35351582	6.51	5.09	145	225	3.4
1	15.029	3.74033274	6.81	4.37	126	164	3.38
1	2.13211111	1.99228243	3.25	4.49	128	210	2.75
1	2.46966667	1.75074648	7.68	5.4	174	152	3.12
1	4.26448889	2.64618544	4.19	5.04	160	195	2.66
1	1.02723556	1.43274847	6.29	4.47	102	485	2.85
1	10.7672267	1.55350228	7.96	5.04	165	187	26.1
1	4.32258667	1.51025826	8.98	5.35	155	332	3.07
1	2.72107111	-0.0716034	6.85	4.02	130	260	2.03
1	6.78053333	2.07763992	4.71	5.43	173	183	2.46
1	1.7324	2.49669522	4.65	4.62	145	179	4.38
1	3.68616889	1.94233233	5.92	4.7	153	173	1.96
1	3.89422222	2.01875559	5.29	4.71	145	245	3.01
1	6.14298667	1.54489939	7.22	5.16	152	146	2.75
1	1.25473333	2.63745605	4.51	5.21	157	227	2.58
1	1.82485333	1.79794033	6.1	4.86	145	205	3.33
1	1.51324444	2.40346609	5.48	5.19	137	211	3.15
0	2.36168444	3.14623631	5.96	5.09	157	239	4.92
1	1.91471111	1.71201255	7.59	5.24	153	304	2.24
1	2.74835556	2.31031514	4.9	5.09	153	216	2.66
1	3.44631111	1.56149689	3.71	5.41	130	228	2.99
1	22.7616267	2.37201787	6.34	4.47	138	169	3.25
1	5.13654222	1.72533473	5.89	5.49	154	358	2.35
1	1.01451111	2.53280753	7.16	5.14	156	228	3.15
1	2.60496	0.91456925	4.74	5.62	165	263	2.35
1	1.57093333	2.23569715	6.91	4.11	123	244	3.76
1	1.55878667	3.40507486	5.02	5.41	164	181	2.2
1	3.02478222	2.28089148	6.96	5.46	167	270	2.8
1	1.18528	1.72281124	10.03	5.04	148	336	3.38
1	4.00130667	3.74650783	4.8	5.32	164	287	2.5
1	0.77752889	3.3288023	9.5	5.09	142	331	3.12
1	2.13042667	2.88752883	4.88	5.51	172	291	25.4
1	0.91256	2.11137011	6.29	5.11	145	321	2.43
1	3.52527111	2.57056433	6.6	5.11	150	278	2.73
1	11.5051467	2.46205162	6.33	4.94	150	224	2.36
1	0.91188	0.93173092	10.61	4.45	135	232	3.74
1	1.84576	2.81586371	6.46	4.47	126	181	3.45

1	14.5274133	2.75946549	6.97	4.12	128	209	2.75
1	5.5896	2.85513598	4.94	4.65	131	230	2.21
1	1.22041333	2.79101172	5.05	4.88	142	204	2.67
1	2.10256	2.19573458	4.63	4.8	145	154	2.27
1	1.24712	1.16658241	5.02	4.89	150	280	2.76
1	1.17189333	1.18387213	4.28	4	122	203	3.74
1	13.2423733	2.66017212	15.04	3.85	113	571	5.85
1	2.66206222	1.29680719	4.64	4.33	142	66	4.79
1	1.26607556	0.37049402	5.68	5.1	144	235	2.21
1	4.77306222	2.29423029	7.87	4.16	126	264	3.3
1	6.63197333	1.24432749	4.19	4.1	121	248	3.45
0	2.16533333	1.55935441	3.6	4.92	151	238	2.63
1	0.62919111	1.57069708	6.55	4.11	76	326	2.86
1	2.00063111	1.28193335	5.72	5.17	154	202	2.67
1	23.8018667	2.00556623	6.38	4.08	119	59	2.04
1	2.00494222	1.1409052	7.45	4.19	123	157	3.27
1	4.97098667	0.90999093	4.86	4.65	143	221	2.95
1	1.28135111	2.43828226	7.38	5.43	160	246	3.47
1	1.67091556	1.90195826	6.19	4.98	150	201	2.8
1	1.86985333	2.2799304	6.39	4.69	145	243	4.33
1	3.05956444	1.86276143	4.96	4.44	139	266	2.95
1	1.79274667	2.13382319	7.68	4.42	133	309	2.54
1	0.06946667	3.6121064	7.3	4.19	112	382	3.54
1	2.53048889	1.60265496	6.51	4.37	135	145	6.59
1	5.33082667	2.44772386	7.46	4.49	129	202	4.22
1	0.39780889	2.15654146	6.4	4.71	147	315	2.23
1	44.1237333	1.57716262	6.35	4.8	142	190	5.24
1	0.32013333	2.20091221	5.96	4.41	128	278	3.76
1	0.76267556	1.91105248	4.31	4.87	151	171	2.75
1	6.37185333	1.56720868	4.33	5.33	155	183	2.75
1	2.19786667	2.8425199	6.32	4.67	137	270	3.38
1	5.41984889	2.27211558	6.58	4.1	128	193	2.89
1	1.47753333	1.25600058	3.82	5.4	161	220	2.8
1	3.24130667	2.18571414	6.19	3.99	120	265	3.47
1	2.10455556	1.16826272	4.52	3.36	97	244	5.57
1	17.056	2.41470322	4.25	5.09	162	155	3.93
1	1.82024444	1.57683207	6.9	4.2	145	156	2.95
1	13.4918489	3.26283146	9.99	5.63	162	273	3.67
1	1.79774222	2.40515973	5.54	4.84	149	355	2.23
1	2.15278222	1.6265015	6.45	4.97	153	261	3.12
0	1.47663111	1.63092539	8.41	5.01	139	281	3.6
1	1.18377778	2.45691861	6.04	4.75	155	249	3.6
1	1.56384	2.2071309	5.62	5	156	169	2.66
1	2.448	1.65327296	5.56	4.91	152	213	2.05
1	6.7032	3.13346825	7.76	5.45	155	265	3.45
1	4.2112	2.83553418	7.68	5.49	172	305	2.58

1	8.09472	1.9124937	4.38	4.21	133	112	2.2
1	4.48096	1.87629208	6.57	3.93	114	210	3.93
1	2.44972	2.34059353	3.32	4.88	158	157	2.95
1	6.04592889	1.90031488	9.95	5.68	154	224	4.44
0	9.63707111	2.03225166	6.22	4.59	145	146	2.9
1	3.34444444	2.06158302	5.54	4.74	143	225	3.93
1	5.87248	1.2039328	4.36	4.34	134	218	4.33
1	28.4944978	2.44122902	5.76	4.84	138	286	3.52
1	1.33472	0.66173391	6.96	4.52	127	422	3.93
1	3.85508444	1.60623278	5.05	5.27	162	217	2.36
1	7.25457778	3.46548587	9.23	5.41	162	280	3.12
1	3.30584	2.89225332	9.83	5.42	158	247	3.6
1	0.42252	2.58396736	8.56	5.63	163	299	3.6
1	5.0456	1.81353727	5.17	4.51	133	192	2.39
1	2.41108	1.42587559	7.19	3.18	93	152	4.68
1	0.83004444	1.32218242	5.67	5.41	150	166	3.01
1	2.06106667	2.88410494	3.99	5.31	161	150	2.05
1	2.25624889	2.47019479	3.9	5.45	164	205	2.43
1	2.47658667	2.2503071	10.71	6.22	174	240	4.02
1	4.80312444	2.51879265	6.79	4.86	148	151	4.39
1	5.30837333	2.35742833	4.5	4.11	114	271	3.84
1	3.58058667	2.39629399	6.67	6.17	188	184	2.94
1	3.68106667	2.90788418	6.22	5.1	150	228	2.17
0	0.63085333	1.92171991	4.3	4.54	121	297	3.12
1	2.1132	1.7947799	5.07	4.62	129	277	15.7
1	0.16728	2.29051251	5.43	4.46	138	252	3.26
1	0.88619111	1.62871099	4.65	4.78	137	216	2.32
1	2.30981333	2.02258014	6.87	5.19	151	222	2.2
1	2.32677778	2.83629096	5.52	5.4	166	217	3.45
1	4.68906667	2.69860574	7.28	5.02	158	209	3.12
1	0.48015556	3.14425788	5.65	4.65	135	340	3
1	1.96654222	2.94448108	5.03	4.76	145	201	1.91
1	2.22762667	2.09753928	4.06	5.1	152	186	2.2
1	4.17718222	1.61490295	6.38	5.38	159	175	2.67
1	4.57744	1.88719085	6.53	4.53	143	152	3.31
1	0.06983111	0.06952606	4.63	3.87	121	161	2.54
1	1.77656889	4.02860977	7.81	6.04	180	305	2.58
1	2.60393333	1.8236618	6.33	4.88	146	228	3.25
1	3.21624889	1.51564358	6.9	4.59	131	313	3.33
1	20.3468222	1.80920638	12.43	4.86	148	403	4.29
1	3.74122667	1.93547013	6.05	5.87	170	213	3.57
1	1.44362667	2.52624051	3.72	4.88	150	151	2.09
1	10.4110222	2.33626162	5.5	4.52	134	197	2.66
1	1.30114222	3.03533753	7.81	5.63	164	304	2.39
1	2.37617778	2.28523546	7.56	4.69	133	157	3.76
1	3.41814222	0.92902925	6.04	5.57	151	285	2.8

1	1.77557333	1.95907314	7.86	4.64	135	290	2.23
1	4.43456	2.93967502	8.18	5.57	161	278	2.71
1	0.45537333	2.38915262	5.05	4.16	116	295	3.52
1	0.86042667	2.98512623	7.88	4.81	136	243	3.84
1	1.7666	3.38203495	4.75	4.94	160	216	2.23
1	2.9726	3.13762238	4.81	5.02	147	164	2.54
1	4.98394667	3.35562454	7.07	5.62	176	272	4.1
1	10.4110222	2.33626162	5.5	4.52	134	197	2.66
1	2.44615111	2.58009561	5.22	5.57	165	299	2.58
1	1.66936	2.55781628	6.31	5.36	158	208	2.85
1	2.47632	1.87506608	5.84	4.73	135	229	2.14
1	3.9292	1.93908692	4.91	4.8	126	199	3.93
1	2.16044889	0.62443998	7.22	5.06	155	248	2.66
1	0.31269333	1.74011524	3.84	3.82	110	215	3.25
1	4.27765333	1.8745369	16.11	4.15	122	295	6.43
1	2.99322222	2.56251949	8.11	5.63	159	212	2.83
1	1.61814667	3.7743872	7.98	5.37	159	217	2.77
1	0.70627556	2.57331046	7.28	4.13	129	248	3.57
1	4.10906667	2.00171646	7.47	5.83	152	274	3.19
1	0.51386222	2.11703834	6.41	3.9	107	306	2.77
1	2.48668	1.72554844	15.66	7.6	192	343	2.23
1	1.17176889	1.97358785	7.76	4.26	135	191	3.31
1	1.48055556	1.75877129	4.42	5.76	171	149	2.88
1	1.98680889	2.12226154	7.15	5.3	154	402	3.26
0	36.2302	2.48211108	7.85	5.45	160	244	2.66
1	1.09028444	1.96856585	6.68	4.55	135	316	4.39
1	2.35072889	3.13739458	10.12	5.61	164	231	5.36
1	1.94245333	1.92340158	5.75	4.1	131	93	2.43
1	6.7896	1.46884162	6.42	5.4	159	232	2.58
1	1.2788	2.55843975	8.82	5.03	140	357	1.99
1	3.50068444	1.86626379	6.36	5.1	143	224	2.26
1	1.74245333	4.02716611	7.79	4.88	138	236	3.57
1	5.51156	2.94130249	6.79	3.4	73	367	3.57
1	15.6198578	1.0018811	8.64	4.25	134	201	3.74
1	4.46272	2.02602758	7.46	4.98	155	165	2.77
1	5.20566667	1.67642344	4.88	4.73	140	164	1.92
1	2.812	2.19922258	5.11	4.62	150	210	3.41
1	11.6270311	3.3752811	6.13	5.66	153	185	2.72
1	0.59132889	3.9080451	4.88	5.93	181	208	2.12
1	3.87131556	2.74650139	7.36	5.62	178	222	3.06
1	3.68291556	1.5663947	6.39	4.68	141	247	2.9
1	1.53817778	2.46171479	5.15	5.12	145	221	2.9
1	3.1304	2.28279047	6.05	4.43	122	338	3.65
1	3.07845333	2.1124894	6.36	4.18	129	246	3.66
1	10.6337067	2.11916093	4.81	4.19	120	191	3.59
1	1.49660444	2.24931616	5.77	4.36	131	199	2.54

1	6.24096	3.07265165	6.95	4.95	146	205	2.17
1	2.99946667	1.24530667	5.82	5.38	158	311	2.67
1	0.64752	1.49256696	4.5	5.37	159	153	2.62
1	2.05628444	4.02974382	5.52	5.83	166	272	2.67
0	4.22577333	1.51204557	6.7	5.11	151	199	2.23
1	1.44449778	2.50002512	8.03	4.25	123	256	5.85
1	0.27756	2.5145059	6.9	4.84	151	270	3.52
1	10.9130933	2.59481804	5.3	4.34	130	268	3.06
1	4.53973333	4.50402854	6.68	4.61	142	359	3.01
1	2.5914	1.97720115	6.81	5.28	143	261	2.55
1	4.02088889	1.76991426	6.28	4.73	138	362	3.12
1	2.18672	3.01426939	8.68	5.39	158	239	1.92
1	0.3248	1.72719428	3.57	4.37	133	241	2.96
1	3.57664444	2.03959563	7.09	4.63	129	207	2.47
1	1.6632	0.44980092	7.32	4.18	142	329	2.89
1	0.67137778	2.45771529	5.59	5.49	159	254	2.55
1	3.83032	1.84631078	3.8	4.59	140	185	2.67
1	2.17750222	1.31935296	4.22	5.24	155	215	2.79
1	3.77916889	2.54251496	7.92	5.38	161	309	2.81
1	2.48408889	1.88115827	6.8	4.7	143	254	2.11
1	3.2368	2.48578959	7.45	5.88	167	185	6.55

DvT	ALT	AST	AST/ALT	TP	ALB	GLD	A/D	
0.11	20	19	1	78.3	47.8	30.5	1.6	
0.15	23	22	1	65.8	44.6	21.2	2.1	
0.23	29	26	0.9	71.2	45.4	25.8	1.8	
0.15	23	19	0.8	69.7	43.1	26.6	1.6	
0.24	19	25	1.3	56.6	38.6	18	2.1	
0.18	12	19	1.6	70	42.3	27.7	1.5	
0.56	13	12	0.9	63	39.1	23.9	1.6	
0.31	30	23	0.8	70.9	44.2	26.7	1.7	
0.12	32	26	0.8	64.9	40.5	24.4	1.7	
0.21	16	14	0.9	58.3	40.8	17.5	2.3	
0.15	57	33	0.6	69.1	46.5	22.6	2.1	
0.1	50	31	0.6	66.5	45.7	20.8	2.2	
0.1	76	42	0.6	77.8	54	23.8	2.3	
0.24	31	19	0.6	71.4	49.6	21.8	2.3	
0.22	13	14	1.1	81.2	41.8	39.4	1.1	
0.12	23	29	1.3	68.9	47.4	21.5	2.2	
0.1	24	21	0.9	72.6	46.2	26.4	1.8	
0.1	33	53	1.6	74.4	46.9	27.5	1.7	
0.1	14	17	1.2	71.4	44.4	27	1.6	
0.14	15	19	1.3	75.2	44.7	30.5	1.5	
0.1	61	38	0.6	74.6	44.7	29.9	1.5	
0.22	28	29	1	73.1	44.4	28.7	1.5	
0.23	19	16	0.8	76.9	45.5	31.4	1.4	
0.14	39	29	0.7	68.5	44.9	23.6	1.9	
0.18	32	32	1	67.5	42.7	24.8	1.7	
0.13	30	48	1.6	65.4	39.6	25.8	1.5	
0.45	11	12	1.1	67.8	37	30.8	1.2	
0.19	12	15	1.3	60.2	37.9	22.3	1.7	
0.22	23	29	1.3	66.9	40.4	26.5	1.5	
0.1	9	13	1.4	72.2	45.6	26.6	1.7	
1.66	29	20	0.7	63.7	45.1	18.6	2.4	
0.16	13	13	1	65	43.3	21.7	2	
0.12	21	16	0.8	69.1	36.7	32.4	1.1	
0.22	16	15	0.9	73.3	47.4	25.9	1.8	
0.15	13	13	1	63.7	40.9	22.8	1.8	
0.18	14	17	1.2	67.1	39.9	27.2	1.5	
0.63	36	24	0.7	66.9	39.9	27	1.5	
0.24	20	22	1.1	72.9	43.7	29.2	1.5	
0.18	11	17	1.5	74.2	42.8	31.4	1.4	
0.15	39	33	0.8	70	45.6	24.4	1.9	
0.1	116	45	0.4	65.8	45.8	20	2.3	
0.17	68	61	0.9	65.8	41.2	24.6	1.7	
0.35	17	19	1.1	71.4	43.4	28	1.6	
0.1	26	29	1.1	70.4	42.9	27.5	1.6	
0.13	91	56	0.6	77.7	47.6	30.1	1.6	

0.1	10	13	1.3	59.1	35.7	23.4	1.5
1.14	28	37	1.3	69	39.2	29.8	1.3
0.8	170	109	0.6	71.6	44	27.6	1.6
0.1	24	15	0.6	62.4	41.2	21.2	1.9
0.13	33	29	0.9	71.4	43.9	27.5	1.6
0.17	42	33	0.8	75.3	45.2	30.1	1.5
0.19	18	13	0.7	58.8	37.8	21	1.8
0.21	21	17	0.8	65.9	41.9	24	1.7
0.35	14	15	1.1	70.2	40.8	29.4	1.4
0.1	74	84	1.1	79.9	46.6	33.3	1.4
0.14	18	21	1.2	69.5	38.8	30.7	1.3
0.16	24	19	0.8	63.8	42.4	21.4	2
0.1	29	16	0.6	60.4	36.3	24.1	1.5
0.27	9	15	1.7	66.4	40.2	26.2	1.5
0.18	18	18	1	69.4	43.8	25.6	1.7
0.37	24	21	0.9	73.5	47.5	26	1.8
0.72	17	19	1.1	58.7	36.1	22.6	1.6
0.1	30	25	0.8	70	47.6	22.4	2.1
0.1	50	26	0.5	72.4	45.8	26.6	1.7
0.27	16	28	1.8	69.9	39.7	30.2	1.3
0.15	57	36	0.6	65.5	45.9	19.6	2.3
0.1	8	10	1.3	70.2	40.8	29.4	1.4
0.33	49	41	0.8	71.8	43.1	28.7	1.5
0.13	19	20	1.1	69	38.8	30.2	1.3
0.33	34	36	1.1	71.7	44.8	26.9	1.7
0.54	48	39	0.8	65.2	39.4	25.8	1.5
0.1	25	15	0.6	69.9	41.2	28.7	1.4
0.2	24	17	0.7	71.3	45.7	25.6	1.8
0.33	22	17	0.8	71.1	43.4	27.7	1.6
0.17	15	17	1.1	69	43.2	25.8	1.7
0.1	64	36	0.6	60.1	38.5	21.6	1.8
0.23	21	19	0.9	63.9	35.9	28	1.3
0.21	9	13	1.4	61.1	36.9	24.2	1.5
0.62	12	17	1.4	73	41.9	31.1	1.3
0.42	26	20	0.8	70.4	43.8	26.6	1.6
0.14	10	13	1.3	59	40.1	18.9	2.1
0.23	39	19	0.5	67.3	43.9	23.4	1.9
0.88	13	19	1.5	74.6	44.4	30.2	1.5
0.24	26	20	0.8	72.1	43.6	28.5	1.5
0.21	20	17	0.9	67.4	42.3	25.1	1.7
0.1	15	11	0.7	73.3	44.6	28.7	1.6
0.1	30	20	0.7	63	41.7	21.3	2
0.1	36	23	0.6	73.1	43.7	29.4	1.5
0.15	16	13	0.8	64.6	42.8	21.8	2
0.89	17	15	0.9	65	43.5	21.5	2
0.35	16	16	1	66.2	43.9	22.3	2

0.11	83	50	0.6	68.7	42.2	26.5	1.6
0.23	7	15	2.1	70.1	39.6	30.5	1.3
0.11	30	17	0.6	74.8	47.2	27.6	1.7
0.1	130	76	0.6	71.3	45.4	25.9	1.8
0.21	11	20	1.8	79.7	49.9	29.8	1.7
0.18	12	16	1.3	72.8	44.5	28.3	1.6
0.51	16	21	1.3	67.8	37.4	30.4	1.2
0.52	18	17	0.9	68.6	43.2	25.4	1.7
1.03	21	21	1	73.7	42.9	30.8	1.4
0.15	115	74	0.6	71.1	43.6	27.5	1.6
0.14	15	14	0.9	64.4	39.6	24.8	1.6
0.27	18	14	0.8	72.3	39.8	32.5	1.2
0.3	51	36	0.7	54.9	36.5	18.4	2
0.15	17	19	1.1	71.5	41.5	30	1.4
0.22	36	23	0.6	78	45.1	32.9	1.4
0.05	32	24	0.8	73.2	49.1	24.1	2
0.24	41	30	0.7	70.8	44.4	26.4	1.7
0.6	17	17	1	65.8	40.5	25.3	1.6
0.23	12	16	1.3	71.1	42.4	28.7	1.5
0.17	27	27	1	78.5	49.9	28.6	1.7
0.12	20	15	0.8	63.1	41.3	21.8	1.9
0.26	30	15	0.5	65.6	40.3	25.3	1.6
0.21	22	24	1.1	79.4	43.8	35.6	1.2
0.38	13	13	1	62.2	39.9	22.3	1.8
0.32	27	22	0.8	66.5	40	26.5	1.5
0.1	31	18	0.6	73.1	47	26.1	1.8
0.14	24	17	0.7	68.3	39.9	28.4	1.4
0.21	21	20	1	62.5	41	21.5	1.9
0.12	23	14	0.6	73.5	45.9	27.6	1.7
0.4	8	13	1.6	71.2	42.3	28.9	1.5
0.33	37	26	0.7	65.2	41.7	23.5	1.8
0.33	34	29	0.9	64.8	41.1	23.7	1.7
0.28	30	20	0.7	76.9	44.3	32.6	1.4
0.2	22	19	0.9	66	44.4	21.6	2.1
0.68	21	19	0.9	75.6	40.6	35	1.2
0.2	19	22	1.2	59.4	37.1	22.3	1.7
0.17	10	16	1.6	72	45	27	1.7
0.4	12	17	1.4	72.5	44.9	27.6	1.6
0.14	62	30	0.5	76.1	42.6	33.5	1.3
0.5	21	19	0.9	69.2	41.1	28.1	1.5
0.38	20	23	1.2	59.4	38.7	20.7	1.9
0.17	72	41	0.6	72.1	46.9	25.2	1.9
0.49	14	14	1	63.8	37.5	26.3	1.4
0.23	11	11	1	66.6	41	25.6	1.6
0.19	12	13	1.1	63.1	39.5	23.6	1.7
0.23	72	82	1.1	78.6	44.4	34.2	1.3

0.15	43	22	0.5	77.4	48	29.4	1.6
0.54	17	19	1.1	71.1	43.4	27.7	1.6
0.1	10	10	1	58.7	39.9	18.8	2.1
0.38	24	16	0.7	74.6	43.8	30.8	1.4
0.1	11	14	1.3	70.8	46.4	24.4	1.9
0.29	20	18	0.9	69.7	46.7	23	2
0.25	92	45	0.5	67.7	47	20.7	2.3
0.1	14	13	0.9	75.5	46.5	29	1.6
0.19	15	16	1.1	67.7	46.1	21.6	2.1
0.29	23	20	0.9	72	41.4	30.6	1.4
0.1	11	14	1.3	72.3	45.9	26.4	1.7
0.06	13	14	1.1	64.8	42.1	22.7	1.9
0.22	21	22	1	72	41.8	30.2	1.4
0.22	40	33	0.8	71.8	44.3	27.5	1.6
0.13	56	59	1.1	76.1	44.3	31.8	1.4
0.25	32	27	0.8	70.6	47.9	22.7	2.1
0.19	14	16	1.1	75.1	41.8	33.3	1.3
0.11	30	16	0.5	71.8	43.5	28.3	1.5
0.13	14	18	1.3	68.7	39.7	29	1.4
0.13	15	16	1.1	72	44.5	27.5	1.6
0.2	19	22	1.2	54.3	35.1	19.2	1.8
0.1	37	23	0.6	71.8	45.1	26.7	1.7
0.14	6	12	2	69.8	44.1	25.7	1.7
0.44	37	23	0.6	66.6	37.6	29	1.3
0.1	17	17	1	64	37.8	26.2	1.4
0.21	18	15	0.8	66.3	41.6	24.7	1.7
0.1	25	22	0.9	68.1	45	23.1	1.9
0.31	17	23	1.4	68	40	28	1.4
0.52	36	18	0.5	65.6	45.6	20	2.3
0.15	146	94	0.6	68.6	40.3	28.3	1.4
0.1	21	19	0.9	74	45.5	28.5	1.6
0.1	20	21	1.1	74.3	46.3	28	1.7
0.1	24	23	1	73.6	46.1	27.5	1.7
0.12	11	17	1.5	69.3	46.5	22.8	2
0.1	10	12	1.2	71.1	44.2	26.9	1.6
0.13	11	14	1.3	72.7	47.3	25.4	1.9
0.8	16	26	1.6	73	43.3	29.7	1.5
0.46	14	18	1.3	72.4	46.8	25.6	1.8
0.15	16	14	0.9	71	46.4	24.6	1.9
0.1	18	16	0.9	75	45.4	29.6	1.5
0.19	14	12	0.9	68.6	45.1	23.5	1.9
0.36	13	17	1.3	72.7	43.5	29.2	1.5
0.22	16	14	0.9	63.8	40.9	22.9	1.8
4.2	50	28	0.6	71.3	45.6	25.7	1.8
0.53	9	12	1.3	72.3	44.7	27.6	1.6
0.37	21	15	0.7	72.6	42.8	29.8	1.4

1.13	16	14	0.9	59.4	34.7	24.7	1.4
0.11	29	21	0.7	64.2	43.8	20.4	2.1
0.14	18	14	0.8	64.3	42.6	21.7	2
0.32	6	14	2.3	78.2	48.4	29.8	1.6
0.12	12	21	1.8	66.9	42	24.9	1.7
0.92	47	28	0.6	72.3	45.7	26.6	1.7
1.01	17	20	1.2	63.9	41	22.9	1.8
0.18	47	23	0.5	61.6	36.9	24.7	1.5
0.44	17	16	0.9	63.2	39.2	24	1.6
0.1	40	27	0.7	75.8	46.4	29.4	1.6
0.43	63	91	1.4	75.8	43.2	32.6	1.3
0.1	15	14	0.9	66.4	41.1	25.3	1.6
0.15	24	17	0.7	68.4	45.1	23.3	1.9
0.1	20	17	0.9	69.5	44.4	25.4	1.7
0.16	12	13	1.1	68.2	47	21.2	2.2
18.1	22	22	1	75.3	46.5	28.8	1.6
0.11	39	40	1	68.4	44.9	23.5	1.9
0.38	11	17	1.5	66.3	43	23.3	1.8
0.1	29	19	0.7	76.8	47.7	29.1	1.6
0.3	26	18	0.7	62.2	36.4	25.8	1.4
0.1	25	22	0.9	71.8	45.1	26.7	1.7
0.15	38	20	0.5	67.6	43.3	24.3	1.8
0.34	104	67	0.6	73	46.4	26.6	1.7
0.19	28	20	0.7	68.2	44.8	23.4	1.9
0.27	19	17	0.9	70.4	38.4	32	1.2
0.12	11	16	1.5	68.1	43.2	24.9	1.7
0.16	10	12	1.2	71.1	46.3	24.8	1.9
0.3	22	20	0.9	73.9	45.7	28.2	1.6
0.13	19	14	0.7	67	43.6	23.4	1.9
0.1	57	35	0.6	67.1	42.7	24.4	1.8
0.39	14	20	1.4	66.5	38.3	28.2	1.4
0.15	35	21	0.6	69.8	44	25.8	1.7
0.16	18	18	1	77.5	49	28.5	1.7
0.1	15	20	1.3	67.3	45.1	22.2	2
0.24	16	14	0.9	76.2	45.8	30.4	1.5
0.1	16	15	0.9	62.4	41	21.4	1.9
0.1	20	18	0.9	74	47.9	26.1	1.8
0.33	15	14	0.9	69	45.1	23.9	1.9
0.1	45	48	1.1	71.3	48.9	22.4	2.2
0.1	11	12	1.1	70.5	44.9	25.6	1.8
16.1	21	16	0.8	73.5	48.5	25	1.9
0.17	28	23	0.8	72.9	42.4	30.5	1.4
0.17	30	25	0.8	68.4	44	24.4	1.8
0.13	21	13	0.6	69.9	45.1	24.8	1.8
0.34	11	16	1.5	61.9	38.3	23.6	1.6
0.29	5	11	2.2	60.4	37.4	23	1.6

0.5	18	17	0.9	71.3	41.2	30.1	1.4
0.28	20	20	1	72.8	42.4	30.4	1.4
0.28	27	21	0.8	63.4	42.3	21.1	2
0.18	31	22	0.7	67.2	43.8	23.4	1.9
0.12	21	18	0.9	72.8	46.1	26.7	1.7
0.24	18	23	1.3	73.1	44.7	28.4	1.6
0.61	11	11	1	55.7	32.2	23.5	1.4
1.14	13	62	4.8	67.5	37.4	30.1	1.2
0.1	14	18	1.3	64.9	42.2	22.7	1.9
0.3	25	19	0.8	73	42.2	30.8	1.4
0.15	22	19	0.9	73.6	45	28.6	1.6
0.12	16	15	0.9	68.1	48.1	20	2.4
1.09	18	31	1.7	75	41.6	33.4	1.2
0.1	18	20	1.1	68.2	45.6	22.6	2
0.78	28	19	0.7	59.5	40.3	19.2	2.1
0.53	15	18	1.2	67.7	41.3	26.4	1.6
0.11	24	27	1.1	56.2	36.8	19.4	1.9
0.1	32	22	0.7	66.7	39.4	27.3	1.4
0.13	28	22	0.8	74	44.3	29.7	1.5
0.59	14	15	1.1	65.1	41.4	23.7	1.7
0.17	34	69	2	67.1	39.7	27.4	1.4
0.16	11	11	1	76.7	47.9	28.8	1.7
0.35	15	11	0.7	69.9	37.7	32.2	1.2
1.45	14	17	1.2	74.1	44.2	29.9	1.5
0.1	22	16	0.7	63	39.4	23.6	1.7
0.1	8	11	1.4	68.9	45.5	23.4	1.9
0.61	15	16	1.1	72.7	44.6	28.1	1.6
0.18	11	12	1.1	63.5	38.1	25.4	1.5
0.29	17	22	1.3	68.1	41.6	26.5	1.6
0.12	15	16	1.1	68.1	44.9	23.2	1.9
0.22	19	18	0.9	69.7	43.8	25.9	1.7
0.28	16	14	0.9	63.2	41.5	21.7	1.9
0.14	87	51	0.6	74.3	49.2	25.1	2
0.45	18	21	1.2	76.6	42.9	33.7	1.3
1	17	11	0.6	72.4	38.5	33.9	1.1
0.43	47	32	0.7	58.7	37.4	21.3	1.8
0.24	16	14	0.9	63.3	40.2	23.1	1.7
0.27	31	18	0.6	74.1	43.3	30.8	1.4
0.32	9	12	1.3	64.7	42.5	22.2	1.9
0.14	12	12	1	71	44.3	26.7	1.7
0.11	23	13	0.6	71.5	41.4	30.1	1.4
0.25	12	22	1.8	63.3	42.4	20.9	2
0.24	58	33	0.6	61	34.5	26.5	1.3
0.11	24	22	0.9	75.6	47.6	28	1.7
0.7	21	13	0.6	61.2	38.3	22.9	1.7
0.1	22	34	1.5	74	45.5	28.5	1.6

0.37	98	75	0.8	71.7	37.9	33.8	1.1
0.53	35	32	0.9	66.6	38.4	28.2	1.4
0.28	21	17	0.8	68.5	42.2	26.3	1.6
0.68	27	22	0.8	66.3	37.6	28.7	1.3
0.1	13	15	1.2	56.4	37.5	18.9	2
0.18	11	19	1.7	70.6	42	28.6	1.5
0.36	14	19	1.4	68.4	40.7	27.7	1.5
0.71	23	22	1	68	40.6	27.4	1.5
0.13	16	14	0.9	68.6	44.8	23.8	1.9
0.11	36	24	0.7	67.5	43.4	24.1	1.8
0.1	17	13	0.8	60.2	37.3	22.9	1.6
0.11	33	22	0.7	63.6	40.8	22.8	1.8
0.16	15	13	0.9	72.5	48.1	24.4	2
0.28	17	19	1.1	66	39.9	26.1	1.5
0.53	36	27	0.8	65.9	40.1	25.8	1.6
0.18	25	23	0.9	68.7	44	24.7	1.8
0.19	50	33	0.7	65.6	44.4	21.2	2.1
0.18	22	19	0.9	73.2	46.6	26.6	1.8
0.17	30	16	0.5	73.3	46.8	26.5	1.8
0.26	25	22	0.9	58.5	35.1	23.4	1.5
0.7	15	16	1.1	62.7	36.9	25.8	1.4
0.12	27	22	0.8	71	46.3	24.7	1.9
0.32	18	18	1	69.1	42.6	26.5	1.6
0.15	24	15	0.6	70.8	45.2	25.6	1.8
0.08	30	18	0.6	67.2	43.4	23.8	1.8
0.19	13	12	0.9	53.5	31.3	22.2	1.4
0.13	16	22	1.4	57	34.4	22.6	1.5
0.14	19	17	0.9	67.3	43.6	23.7	1.8
0.16	24	20	0.8	79.3	43.2	36.1	1.2
0.38	29	22	0.8	73.9	41.8	32.1	1.3
0.29	15	16	1.1	79.3	45.3	34	1.3
0.13	27	24	0.9	74.1	46.2	27.9	1.7
0.42	27	19	0.7	72.4	44.4	28	1.6
0.24	20	23	1.2	65.7	42.2	23.5	1.8
0.49	26	27	1	72.6	43.1	29.5	1.5
0.12	12	21	1.8	66.9	42	24.9	1.7
0.21	39	31	0.8	68	41.9	26.1	1.6
0.11	14	13	0.9	66.2	44.5	21.7	2.1
0.19	20	19	1	79	47.7	31.3	1.5
0.28	22	18	0.8	60.6	38.2	22.4	1.7
0.1	12	13	1.1	76.9	47	29.9	1.6
0.1	24	15	0.6	68.9	41.7	27.2	1.5
0.37	37	30	0.8	61.1	39.2	21.9	1.8
0.11	31	22	0.7	70.1	45.7	24.4	1.9
0.39	41	33	0.8	58.3	31.9	26.4	1.2
0.63	17	19	1.1	64.6	40.7	23.9	1.7

0.22	12	10	0.8	66.6	41.1	25.5	1.6
0.46	59	26	0.4	72.6	43.5	29.1	1.5
0.28	19	14	0.7	69.4	41.3	28.1	1.5
0.18	10	11	1.1	66.8	41.5	25.3	1.6
0.24	24	31	1.3	61.7	42.2	19.5	2.2
0.2	22	18	0.8	72.1	45.9	26.2	1.8
0.37	42	19	0.5	69.3	46.4	22.9	2
0.37	37	30	0.8	61.1	39.2	21.9	1.8
0.1	38	21	0.6	67.7	44.6	23.1	1.9
0.1	15	14	0.9	70	44.4	25.6	1.7
0.11	25	17	0.7	58.2	38.7	19.5	2
0.25	16	21	1.3	75.4	40.3	35.1	1.1
0.1	21	19	0.9	69.3	45.1	24.2	1.9
0.17	21	17	0.8	68.7	43.1	25.6	1.7
0.74	32	29	0.9	66.8	37.2	29.6	1.3
0.12	19	15	0.8	73.9	46.3	27.6	1.7
0.29	37	29	0.8	76.7	43.7	33	1.3
0.11	13	12	0.9	73.8	46.6	27.2	1.7
0.18	47	53	1.1	82.3	45.7	36.6	1.2
0.52	7	15	2.1	67.9	38	29.9	1.3
0.33	11	12	1.1	57.4	34.3	23.1	1.5
0.37	8	18	2.3	66.6	39.6	27	1.5
0.14	22	18	0.8	68.6	44.9	23.7	1.9
0.19	51	29	0.6	75.2	45	30.2	1.5
0.1	57	30	0.5	72	48.4	23.6	2.1
0.41	19	19	1	75.6	40.5	35.1	1.2
0.25	17	20	1.2	61.4	41.6	19.8	2.1
0.2	14	15	1.1	63.7	39.5	24.2	1.6
0.46	11	18	1.6	73.5	42.1	31.4	1.3
0.14	21	18	0.9	71.5	45.3	26.2	1.7
0.42	24	19	0.8	68.8	39.1	29.7	1.3
0.22	15	12	0.8	74.4	44.4	30	1.5
0.43	17	17	1	75.5	41.4	34.1	1.2
0.19	12	14	1.2	65.9	42.1	23.8	1.8
0.3	41	31	0.8	73.8	46.6	27.2	1.7
0.24	26	23	0.9	66.5	42.4	24.1	1.8
0.39	23	12	0.5	71.1	43	28.1	1.5
0.76	59	37	0.6	69.5	44.6	24.9	1.8
0.1	27	21	0.8	80.1	49.8	30.3	1.6
0.16	35	31	0.9	74.5	45.5	29	1.6
0.34	15	16	1.1	72.6	44.3	28.3	1.6
0.24	20	13	0.7	71.7	43.3	28.4	1.5
0.13	18	16	0.9	66.8	39.3	27.5	1.4
0.33	56	35	0.6	74.9	43.1	31.8	1.4
0.3	24	20	0.8	68.4	43.1	25.3	1.7
0.2	12	14	1.2	68	42	26	1.6

0.19	32	23	0.7	69.2	43.9	25.3	1.7
0.14	16	14	0.9	68.3	44.2	24.1	1.8
0.36	16	18	1.1	69.7	43.3	26.4	1.6
0.1	30	20	0.7	78.3	51.5	26.8	1.9
0.1	18	16	0.9	67.8	41.4	26.4	1.6
0.39	26	27	1	74.6	43.3	31.3	1.4
0.3	12	15	1.3	54.7	33	21.7	1.5
0.25	15	17	1.1	70.7	41	29.7	1.4
0.1	20	19	1	69.1	43.1	26	1.7
0.18	17	16	0.9	64.5	38.1	26.4	1.4
0.14	24	15	0.6	73.5	42.5	31	1.4
0.13	20	19	1	68.2	45.7	22.5	2
0.22	15	30	2	67.9	41.6	26.3	1.6
0.25	24	19	0.8	67	40.2	26.8	1.5
0.12	65	37	0.6	59.4	42.4	17	2.5
0.17	11	13	1.2	69.1	43.7	25.4	1.7
0.32	14	17	1.2	78.7	43.7	35	1.2
0.1	17	20	1.2	65.4	43.9	21.5	2
0.32	26	20	0.8	76.8	44.8	32	1.4
0.42	17	22	1.3	68.9	42.9	26	1.7
0.41	16	17	1.1	78.2	50.1	28.1	1.8

TBIL	CHE	ALP	GGT	PA	UREA	CREA	eGFR
11.2	9543	114	69	0.364	6.17	52.2	114.74
12.7	11999	66	17	0.324	5.43	103.3	70.38
9.5	7822	71	22	0.286	5.65	84.9	89.84
12.7	9651	84	63	0.319	5.9	80.9	88.16
15.6	7294	59	18	0.267	3.1	77.1	81.43
9	7873	86	11	0.241	4.29	54.5	106.43
22.1	8005	108	20	0.23	6.04	57.2	105.95
15.6	9827	97	13	0.261	7.2	54.7	103.35
12.6	8348	45	41	0.259	4.2	45.2	109.28
9.2	8607	47	29	0.293	6.47	54.4	112.81
23.6	12353	153	546	0.38	5.36	52.6	129.8
15.1	6084	87	14	0.251	6.65	57.7	119.8
14.9	15199	97	72	0.31	1.6	56.9	123.92
16.1	10337	93	254	0.423	4.8	89.5	94.98
9	12925	99	28	0.365	9.24	82.3	70.81
19.9	14162	47	54	0.453	3.57	61	117.92
16.6	10917	69	102	0.3	5.23	64.7	120.9
20.3	11218	113	51	0.24	5.05	39	114.71
13.2	9273	76	14	0.342	6.4	92.7	76.91
29.5	11380	102	37	0.369	4.15	46.3	113.08
17.3	10944	93	53	0.329	5.76	59.7	116.48
7.7	11135	50	36	0.097	5.2	61.9	105.49
8.1	9523	105	29	0.354	6.9	84.6	89.6
11	8236	70	17	0.319	3.76	59.5	103.4
10.3	8327	81	19	0.299	5.4	41.8	102.34
14	7148	104	193	0.206	4.5	98.2	81.4
14.3	5232	133	19	0.234	5.28	54	103.28
9.7	7552	78	16	0.361	18.2	332.6	16.64
7.7	10801	123	66	0.22	5.3	86.8	66.39
27.8	10520	58	13	0.211	4.12	46.3	118.78
9.4	10509	88	29	0.251	7.1	63.8	111.77
20.9	8855	107	24	0.345	4.19	76.6	102.23
9.3	8799	52	20	0.281	7.34	61.6	99.92
25.4	7997	99	20	0.233	4	50	105.71
31.5	6832	69	17	0.243	5.06	72	87.97
10.1	9641	81	50	0.269	5.83	56.7	89.38
7.7	6336	71	92	0.181	5.9	43.1	138.91
15.7	9035	84	18	0.252	6.27	65.3	96.19
10.2	9444	121	27	0.223	5.8	92	58.5
17.9	8613	106	508	0.424	4.44	69.4	100.64
12.4	13146	57	80	0.348	3.3	57.3	137.3
17.3	9972	84	30	0.235	2.71	43.2	117.33
16.5	10181	55	16	0.217	5.46	71.2	85.33
9.9	10506	109	65	0.264	5.44	42.2	102.74
25.2	11838	148	51	0.3	5.46	76.7	119.25

15.6	6008	69	8	0.223	7.8	72.3	89.07
10.3	12255	73	26	0.187	9.96	57.8	103.66
9.4	7609	103	48	0.205	3.88	53.5	118.14
6.9	8966	58	20	0.244	4.94	59.2	122.78
23.5	9519	81	25	0.355	5.05	50.7	111.33
31.4	8063	50	28	0.359	6.5	93.3	72.65
16.5	8698	53	31	0.392	7.38	60.4	109.59
13.1	7813	71	46	0.265	6.55	34.6	104.79
13.3	9260	101	27	0.282	3.99	39.9	125.62
26.4	14067	99	75	0.319	3.86	58.6	119.04
19.2	5307	65	17	0.227	6.62	71.9	85.59
14.4	10376	118	21	0.3	7.17	29.1	116.92
15.7	10088	32	45	0.24	3.1	72.1	118.1
9.4	4649	135	17	0.192	6.92	67.4	100.44
6.8	12608	84	30	0.287	4.62	44.9	109.52
15.4	11949	113	18	0.294	4.3	48.9	102.09
14.2	6568	137	19	0.3	14.51	177	31.23
9.7	10558	76	41	0.384	5.97	72.8	102.21
12	10898	83	42	0.392	5.4	48.6	130.37
14.6	6656	106	41	0.223	4.14	57.4	98.62
14.7	12667	116	174	0.42	3.9	55.3	126.27
9.2	11239	71	41	0.363	5.65	59.5	119.13
17.2	10630	106	106	0.322	3.92	59.4	116.73
31.1	9246	71	15	0.275	6.59	51.8	105.22
16.2	9457	105	36	0.323	4.77	43.6	103.8
22	7783	88	154	0.245	5.05	68.5	104.07
15.7	10605	113	46	0.369	4.59	55.4	122.68
27.7	10917	104	23	0.348	3.83	59.1	107.51
11.6	10492	94	63	0.051	6.47	63.6	106.54
12.5	8396	72	52	0.332	4.6	58.8	126.63
11.8	7401	68	80	0.238	3.57	41.2	124.3
15.2	9212	69	23	0.21	2.89	57.2	113.66
9.3	5191	82	14	0.118	4.9	56.8	123.14
10.1	7977	125	22	0.254	4.9	57.3	96.22
15.5	10187	135	52	0.32	5.56	57.7	98.05
17.1	6610	53	20	0.252	7.7	57.8	102.57
12.1	12503	111	39	0.299	5.11	62.3	116.08
15.6	9743	93	43	0.235	2.9	41.6	138.65
11.6	10752	73	30	0.312	4.68	41.9	116.04
14.1	6634	114	12	0.204	8.23	55.2	107.51
12.4	6845	142	21	0.086	7.42	46.6	120.75
22.3	12769	63	25	0.348	4.9	63	110
20.8	8577	100	29	0.308	7.32	80.5	91.86
21.8	11019	118	29	0.267	4.44	72.8	100.79
16.7	9956	65	22	0.342	7	107.9	60.51
18.8	9735	51	43	0.417	7.52	70.2	108.22

15.1	10299	115	20	0.26	5.61	41.3	123.33
13	7888	82	18	0.219	7.41	78.9	67.53
20	13085	91	17	0.291	4	66.1	119
16.5	8414	55	71	0.318	6.44	58.3	110.42
17.6	8661	69	46	0.268	6.96	49	111.78
12.2	10714	111	19	0.34	6.27	49.4	96.87
10.7	7840	94	33	0.228	9.04	79.4	62.47
6.3	9153	67	9	0.252	5.5	43	96.52
10.4	9775	80	33	0.261	6.25	62.8	83.54
15.6	9139	112	95	0.315	5.11	67.5	110.75
13.7	9511	50	11	0.302	4.9	56.7	121.51
14.1	7763	98	33	0.087	5.11	75.2	87.03
17.3	9815	86	31	0.225	4.1	64	107.77
12.1	11418	54	26	0.291	4	41.5	112.39
17.8	13680	92	25	0.398	6.7	105.3	67.33
14.2	12151	98	39	0.471	7.1	74	106.64
12	9808	65	42	0.293	7.77	73.2	99.16
9.4	9332	78	21	0.256	6.12	63.8	83.7
12.1	12060	92	16	0.189	4.26	64	93.31
10.2	10688	48	47	0.348	6.7	52.3	105.63
11.1	9921	58	14	0.245	4.78	56.1	94.21
10.7	7750	141	109	0.286	3.98	62.9	109.31
9.5	11292	95	28	0.305	4.39	68.6	95.6
12.4	8756	125	16	0.223	5.3	61.9	92.31
12.8	9932	50	59	0.404	5.4	49.9	113.65
15.1	9294	64	17	0.29	5.22	56.7	125.86
11.9	6673	94	16	0.295	4.26	70.4	105.1
16.3	9325	80	22	0.275	7.01	90.8	78.31
13.9	9885	68	22	0.332	5.25	42.1	112.65
12.8	7869	58	11	0.288	6.22	70.1	71.61
12.2	9523	59	21	0.321	7.13	78.5	96.35
12.5	7395	137	22	0.245	5.74	56.5	105
11.8	9343	57	17	0.28	5.7	79.5	100.68
17.4	12754	94	50	0.497	5.9	68.2	112.63
24	7613	122	31	0.173	8.72	132	46.11
12.3	8258	66	20	0.23	4.4	68	95.27
14.4	7741	47	18	0.28	5.74	73.3	87.33
13.2	9600	133	31	0.322	8.59	66.5	81.31
10	11524	113	61	0.328	5.6	71.4	97.4
8.6	9998	89	27	0.26	5.43	50.7	98.09
6.5	9124	35	17	0.265	12.3	203.8	22.68
11.9	10034	54	22	0.263	4.45	49.3	120.51
6.1	12347	86	19	0.252	6.59	43.7	95.34
13.7	8547	85	11	0.241	4.36	55.6	101.36
12.4	10629	38	17	0.193	3.98	59.8	111.6
17.2	9441	135	103	0.269	4.05	58.6	98.93

24.2	17379	110	25	0.411	5.4	39.8	164.03
9.2	12791	85	64	0.276	6.42	58.1	97.82
17.1	11376	78	11	0.268	5.39	54.2	143.46
12.9	10092	208	53	0.297	4.54	55.2	121.15
14	10909	94	19	0.339	8.4	75.5	98.59
6.7	10921	55	34	0.211	2.5	50	116.63
15.1	12189	71	48	0.269	5.1	80.7	109.94
15.6	10043	87	21	0.332	3.95	53.4	123.67
14.8	15270	61	47	0.408	8.59	67	105.76
10.4	9696	96	17	0.23	10.16	68.9	75.21
18.3	9343	58	35	0.392	5.96	75.4	94.58
9.9	10803	70	22	0.243	5.3	61.3	97.47
15.7	11287	76	22	0.366	9.55	129	51.58
9.4	8893	102	26	0.226	6.88	53.9	98.18
15.4	9866	63	73	0.262	5.73	56.8	97.87
10	9897	108	40	0.394	5.62	61.7	95.24
10.8	8060	80	13	0.357	10.95	123.2	38.58
20.3	6853	90	35	0.322	7.58	71.8	106.47
9.3	6710	80	13	0.219	7.38	64	87.59
16.7	13916	124	21	0.349	6.19	80.3	100.95
12.3	7213	73	13	0.116	6.3	46.6	99.44
14.9	9982	90	61	0.35	7.12	101.5	73.42
22.7	7515	67	20	0.302	4.89	55.6	101.33
8.5	7888	77	24	0.275	10.29	164.4	34.14
15.4	6970	86	17	0.207	6.2	63.1	95.59
24.4	12517	92	30	0.346	5.55	60.4	110.37
19.5	11034	105	21	0.337	5.68	103.6	71.63
12.9	9169	95	53	0.224	4	51.3	104.82
12.9	9407	63	33	0.381	4.5	67.9	104.45
12.7	11963	75	60	0.307	4.4	55	101.01
14.4	10382	62	114	0.415	5.3	58.1	108.27
12.5	14196	63	34	0.392	6.4	76.3	94.78
13.2	8126	63	35	0.256	5.14	43.8	105.86
15	11944	72	19	0.395	5.76	61.4	98.66
14.8	8395	98	27	0.351	7.98	74.4	95.1
20	8126	54	25	0.272	3.88	64.3	116.2
11.9	10565	94	16	0.303	201	56.7	97.92
11.7	14064	62	29	0.408	6.6	51.3	108.57
24.8	8907	57	28	0.274	6.23	83.3	88.76
20.4	11315	79	33	0.332	6.34	69.9	101.77
14.1	12630	83	34	0.332	5.97	53.6	126.11
14.9	7413	88	34	0.383	13.98	170	38.54
16.5	8121	77	19	0.257	2.88	43	111.08
16.6	10091	72	83	0.328	4.52	64.3	103.12
7.7	9402	88	14	0.188	6.73	63.2	85.86
11.1	8079	168	23	0.333	4.28	64.5	110.49

9.9	8991	137	60	0.271	18.63	172.3	30.71
24.5	9829	75	33	0.284	5.08	73.6	88.42
8.3	8646	78	34	0.369	6.4	71.1	97.57
17.2	9521	81	21	0.364	4.7	67.4	106.25
11.2	6695	66	10	0.214	3.6	48.7	106.63
14.6	8478	99	39	0.376	7.01	81.3	82.27
8.2	7492	90	41	0.287	5.6	125.9	51.29
16.3	10791	93	72	0.226	5.87	62.1	115.42
11.9	7021	33	13	0.297	10.55	76.4	70.21
14.4	7822	115	22	0.321	7.96	83.3	100.02
9.1	10017	124	176	0.264	3.16	33.3	116.66
13.3	8222	66	39	0.258	5.39	52.6	103.23
14.5	10238	85	27	0.304	4.42	58	121.24
20.6	8816	70	28	0.25	4.61	65.2	106.95
19.4	9232	48	11	0.236	4.1	44.7	123.59
18.4	10331	78	21	0.307	6.5	70.2	100.88
15.9	7830	66	34	0.285	4.65	60	108.36
15.9	7714	66	7	0.241	6.6	52.9	104.89
23	10495	119	15	0.262	7.9	63.7	113.42
14.8	7484	51	14	0.198	7.68	55.7	101.97
20.2	13035	74	22	0.295	4.24	47.8	118.14
19.5	9269	75	31	0.264	4.93	56.2	124.55
16	9581	88	45	0.204	5.4	60.9	98.99
15.6	8341	118	22	0.267	5.04	57.1	112.94
9.1	6891	72	28	0.17	6.67	63.4	90.48
13	5578	108	17	0.201	6.52	51.3	101.91
13.1	7577	103	21	0.195	3.27	53.9	125.82
14.1	7273	63	20	0.287	5.53	58.5	104.24
16.7	8283	111	25	0.294	4.99	42.9	136.27
15.3	9818	80	27	0.243	3.9	48.2	124
6.4	9944	76	21	0.226	10.25	65.2	90.36
25.7	9717	65	31	0.38	6	64.6	109.64
11.7	12643	77	22	0.333	7.2	125.9	56.59
18.6	8771	62	22	0.292	7.47	90	74.82
8.6	10865	75	16	0.386	11.1	119.7	57.67
13.8	8377	89	21	0.324	7.33	57	109.12
14.7	13670	81	19	0.323	6.6	55.1	109.11
11.9	12245	56	22	0.305	6.5	51	106.51
26.1	11723	85	567	0.385	4.94	62.8	112.49
10.8	7397	59	23	0.29	10.02	73.9	97.39
19.2	10271	110	55	0.355	5.36	74.7	113.17
15.9	9368	85	35	0.255	5.07	44	107.19
14.6	7027	100	24	0.318	4.99	57.8	110.81
21.2	10414	105	39	0.244	5.18	91.1	94.28
10.4	4329	119	11	0.169	4.93	64.5	102.27
6.6	7127	60	17	0.238	5.19	69.7	91.06

26.1	12490	65	17	0.306	5.4	48.7	107.38
12.6	11658	104	17	0.23	3.8	54	98.81
21.3	11948	85	18	0.293	3.82	42.9	111.16
30.1	10822	65	40	0.274	5.92	55.9	111.56
16.6	7485	71	16	0.281	6.18	55.1	117.88
15.4	7894	77	21	0.317	5.71	44.8	109.6
7.8	6347	97	26	0.195	7.36	124.9	44.92
17.4	4351	97	325	0.197	4.6	56.2	105.23
18.3	7130	66	16	0.242	6.44	55.1	104.61
7.5	11103	80	15	0.232	3.9	42.3	100.52
9.8	10705	58	25	0.272	3.43	48.5	100.94
12.5	9522	41	18	0.354	4.72	66.6	112.15
9.4	7979	102	14	0.203	4.95	58.3	90.45
11.9	10290	84	23	0.295	6.73	68.5	103.34
12	8799	97	28	0.235	6.5	54.7	94.33
6.9	8112	73	11	0.251	4.6	53.9	94.13
10.3	9354	64	18	0.235	4.6	74	91.37
11.1	11298	106	12	0.274	6.48	77.3	101.13
13.2	8366	66	30	0.239	7.36	65.4	99.57
17	10810	84	51	0.324	5.8	75.5	88.73
17.4	10348	61	93	0.268	4.44	42.3	101.23
14.8	13419	68	15	0.276	5.1	28.9	161.88
9.5	11511	83	22	0.247	6.5	89.8	64.62
19.2	7927	73	31	0.166	6.46	76.5	67.68
21.1	7693	69	21	0.251	7.93	51.2	110.88
12.1	9198	96	19	0.415	5.02	40.1	148.2
9.7	9233	126	14	0.256	5.42	43.5	110.66
9.5	9717	75	12	0.201	3.6	35	118.87
15.7	10707	72	26	0.287	7.27	61.9	97.83
9.5	11384	72	14	0.303	4.36	68.3	104.93
10.7	10720	92	22	0.279	4.51	62.2	91.95
15.7	11969	79	15	0.239	6.49	63.1	92.94
15.2	13184	87	89	0.341	4.55	72.3	116.32
7.5	10521	74	18	0.213	4.39	61.8	94.52
8.7	8370	144	87	0.286	10.9	119.9	52.53
5.1	5451	88	213	0.144	4.92	63.8	112.55
7.4	7084	92	33	0.252	5.56	55.4	108.87
16.9	11007	232	42	0.264	3.1	68.1	95.89
12.1	11826	65	10	0.267	4.32	52	102.9
12.2	7028	89	24	0.276	4.87	55.1	103.15
16.7	9820	77	15	0.23	4.34	47.2	132.07
16.8	15643	63	44	0.298	6.76	50.6	128.23
12	6948	56	51	0.218	4.16	60.7	110.14
15.7	9130	51	39	0.349	8.04	84.5	85.42
13.4	10220	97	41	0.218	9.21	95	65.8
26.6	9746	89	39	0.259	4.87	71.8	110.28

14.4	7361	151	51	0.182	7.69	102.6	66.15
7.8	7429	101	125	0.22	7.9	91.4	55.35
16.3	8677	59	25	0.324	6.42	51.2	116.47
8.2	8857	80	29	0.252	12.24	178.2	33.46
10.6	9005	36	15	0.189	5.32	51.6	123.68
20.9	9084	170	22	0.264	4.44	44.5	104.57
10	10094	63	16	0.357	7.38	105.8	68.37
14.3	9879	92	39	0.256	4.12	51.7	100.24
10.7	8815	75	17	0.219	5.5	44.8	121.33
28.6	9494	73	28	0.327	3	77.5	93.51
4.1	11570	79	33	0.189	4.97	63.2	132.81
8.4	11290	96	51	0.353	4.82	59.6	118.21
12.9	11981	123	38	0.388	4.9	66.5	115.42
11.5	7948	66	15	0.203	6.22	78.5	89.18
8.8	8940	80	38	0.241	3.5	63.2	99.51
13.3	9992	65	26	0.253	7.8	62.6	97.88
13.5	9722	62	29	0.226	5.9	65.9	98.56
17.7	11785	110	48	0.343	8.31	57.8	112.38
10.5	13384	98	27	0.297	5.37	69	115.29
10.3	15609	56	44	0.346	7.4	83.8	87.5
13.2	9776	102	9	0.165	5.22	48.1	96.36
11.6	9334	83	24	0.325	7.66	82.7	99.49
20.4	10126	66	20	0.336	7.79	58.3	112.77
8.4	9554	62	16	0.23	5.22	43.7	120.21
14.4	10988	83	20	0.372	3.9	36.5	108.52
6.8	8385	68	12	0.22	5.84	66.1	99.83
8.1	8507	64	20	0.234	5.06	42.2	117.41
19.7	7065	75	23	0.335	6.36	54.3	103.77
12.7	12460	99	27	0.268	13.08	131.1	49.18
12	8676	112	23	0.211	5.5	83.5	79.1
19.6	8918	110	43	0.273	5.24	50.6	100.95
20.2	9287	56	30	0.286	5.2	53.4	104.49
11.6	7938	119	23	0.266	4.28	43	108.01
11.4	7671	86	32	0.283	3.75	62.2	95.42
14.4	9409	75	49	0.288	4.4	70.9	93
11.2	6695	66	10	0.214	3.6	48.7	106.63
15.6	14420	71	36	0.323	6	56.3	138.29
9.9	8963	59	23	0.348	7.21	59.1	116.97
10.8	12632	52	18	0.294	3.89	45.2	110.82
7.2	11910	89	17	0.298	6.14	70.8	79.73
12.2	8262	110	30	0.346	5.54	64.3	105.32
14.8	7959	114	28	0.358	5.53	66.1	105.61
9.6	6121	64	42	0.272	7.95	87.8	74.96
8.9	9274	80	36	0.359	5.64	60.5	103.53
9.1	8105	55	19	0.067	9.31	71.7	72.18
15.3	7713	58	17	0.298	4.5	47.4	98.19

16.2	8337	60	21	0.346	3	49.9	108.79
12.7	12203	111	51	0.301	4.56	52.6	124.44
8.2	10931	129	10	0.227	4.8	36.7	111.41
7.1	9112	119	25	0.43	7.1	70.1	112.15
13	13664	89	48	0.387	5.16	62.6	111.07
14.3	10174	60	28	0.364	5.87	70.2	98.08
13.2	14368	96	36	0.296	5.48	49	143.36
9.6	6121	64	42	0.272	7.95	87.8	74.96
21.9	13499	91	67	0.427	4.7	60.8	116.43
8.1	12996	96	45	0.372	4.44	51.2	125.83
12.4	9483	77	56	0.301	5.7	68.8	106.09
8.9	9609	157	47	0.21	5.96	62.4	86.59
26.3	9062	51	18	0.305	6.3	48.4	113.48
10.8	11561	38	14	0.287	5.7	41	108.18
10.6	9707	89	43	0.137	4.09	55.6	93.17
10.5	14539	112	32	0.283	5.7	71.8	88.08
32.7	7205	114	28	0.243	12.47	171.6	36.02
12.6	9243	71	19	0.281	8.12	82.5	66.74
10.1	11252	88	39	0.267	4.52	42.3	111.69
8.4	6629	55	17	0.277	6.06	83.6	62.09
21.2	5198	112	23	0.222	5.3	57.5	107.22
13.2	7123	65	31	0.216	9.7	71.1	85.38
15.9	9026	89	52	0.24	5.28	58.6	106.38
12.7	10363	91	54	0.296	4.85	67.1	102.05
15.7	10765	71	26	0.355	4.3	58.3	118.46
13	9949	106	78	0.196	8.75	100.9	67.02
19.5	11412	79	39	0.244	4.72	70.2	115.28
15.7	8816	27	8	0.233	5.33	59.8	99.04
9.4	8416	96	23	0.203	4.3	64.5	86.4
13.8	12448	99	17	0.266	4.8	38.4	116.11
14.6	9546	60	16	0.296	6.2	78.8	98.25
11.4	14777	167	24	0.284	8.86	53	98.03
6.3	7567	104	31	0.306	8.98	172.6	30.17
8.6	8817	70	14	0.327	10.52	130.6	35.45
16.2	14775	149	23	0.32	4.2	68	105.86
16.9	8368	92	27	0.257	6.4	87.8	78.19
14.2	10519	96	21	0.258	7.41	59.1	106.58
15.4	17941	70	38	0.353	7.74	55.5	118.36
25.3	12218	93	83	0.401	5.2	75.5	111.88
26.4	13628	92	54	0.407	7.96	81.3	96.01
20.9	10433	116	12	0.276	8	72	83.23
8.7	9759	131	16	0.248	6.76	41.4	111.69
13.5	8942	71	25	0.289	5.59	55.3	98.73
16.9	11953	78	34	0.253	5.1	49.9	109.56
9.1	10262	96	9	0.312	4.8	60.3	93.96
17.4	10712	57	29	0.345	4.7	64.7	104.32

10.5	11424	68	51	0.425	7.3	91	82.61
11.8	10944	67	46	0.366	6.05	68.5	110.08
18.1	6178	98	27	0.312	7.65	51.3	104.08
16.3	16076	84	35	0.515	4.8	73.8	120.31
9.2	9183	51	22	0.306	3.81	76.4	107.5
16.3	12113	96	28	0.179	5.53	68.6	75.08
12.7	11458	96	11	0.194	7.75	65	96.38
7.4	9610	137	17	0.171	4.83	47.6	102.28
16.3	17453	102	38	0.373	4.44	37.8	113.48
12.8	9082	81	15	0.278	3.6	37.1	113.38
9.8	10084	114	33	0.281	5.05	41.8	116.95
14.3	11741	78	69	0.444	6.4	59.8	113.98
21.9	10038	62	43	0.292	6.68	69.3	95.87
9.7	8201	73	16	0.294	10.2	90.9	54.94
10.4	7977	91	39	0.409	4.79	45	106.72
16.1	11831	110	18	0.274	4.62	55.8	110.08
16.3	10052	131	21	0.318	6.5	37.9	107.19
9.1	7939	67	23	0.239	4.5	67.9	112.05
8.9	9864	64	41	0.325	6.54	82	84.33
13.6	6878	90	30	0.244	5.53	43.7	103.73
21.1	11516	69	103	0.332	6.6	83.4	99.18

UA	GLU	Ca	P	Na	K	CL	CO2
310.5	15.35	2.35	1.33	137.2	4.11	103.5	24
229.7	19.68	2.31	0.89	138.2	4.35	104.2	27.6
403.7	13	2.26	0.92	134.9	4.27	101.9	23.1
415.4	10.96	2.33	1.15	133.9	3.63	96.6	28.3
313	6.36	2.22	0.98	143.3	3.52	103.7	26.9
213.5	6.51	2.32	1.26	137.6	4.59	102.6	28.2
298	11.68	2.32	1.24	136.6	4.08	104.6	25.2
316	5.71	2.46	1.54	140.2	4	105.3	22.1
368	6.76	2.27	1.47	141.8	3.56	106.1	27.6
247.9	16.54	2.24	1.13	136.9	3.77	104.9	23.6
332.1	14.43	2.35	1.16	136.3	3.65	100.9	23.9
325.1	9.52	2.35	1.26	132.6	4.23	102.5	20.4
292	10.4	2.43	0.9	138.8	4.04	104.7	24.2
604.4	12.55	2.38	1.27	136.3	3.63	100.2	23.8
383.5	17.65	2.4	1.29	135.4	3.94	98.9	26.1
458.1	7.07	2.35	0.93	139.7	3.94	104.7	23.4
355.5	12.18	2.46	1.23	133.5	4.3	101.4	22.2
276.2	5.94	2.43	1.29	139.7	3.62	103.7	27
410.4	7.57	2.41	1.63	140.6	4.81	100.9	27.3
348.3	15.29	2.51	1.3	137.2	4.14	99.3	25
430.7	8.76	2.36	1.15	141.2	3.93	108	23.5
279	15.37	2.26	0.94	136.7	4.21	99.2	28.3
377	9.91	2.35	0.97	138.5	3.84	101.8	23.7
289.2	5.09	2.29	1.27	137.9	3.96	105.5	27.1
270	9.12	2.43	1.35	137	4.56	101.5	26.5
440	4.13	2.38	1.45	138.7	3.51	100.7	26.2
223.3	7.74	2.22	1.19	137.9	3.67	103.4	28.5
529	4.04	2.18	1.28	141.4	4.6	111.5	19.6
273	7.88	2.27	1.21	143	3.8	106.9	26.3
237.8	10.08	2.4	1.24	139.4	3.83	101.5	28.5
339	13.81	2.22	0.88	137.8	4.1	104.4	24.8
442.1	5	2.32	0.91	142.7	3.46	107.7	26.5
361.2	9.36	2.26	1.11	136.1	3.83	104.9	26.2
205	16.18	2.34	1.09	139.7	4.32	106.9	24.5
330.6	15.86	2.31	1.13	136.5	3.86	100.7	30.1
303.5	9.52	2.26	1.25	134.3	3.97	101.4	25.5
263	7.12	2.28	1.42	140.4	3.6	107.3	26.3
228.8	22.85	2.35	0.98	132.1	3.96	99.7	23.9
266	4.7	2.23	1.51	141.6	3.98	106.6	25.2
268.5	12.71	2.35	1.24	138.5	4.1	99.4	29.5
622	5.86	2.38	1.07	139.7	4.35	108.3	25.7
202.7	12.58	2.31	1.15	137.4	3.65	102.5	27.2
295.2	12.45	2.27	0.85	135	3.84	103.5	23.6
268.2	9.11	2.35	1.13	136.4	4.2	105	23.1
419.8	15.22	2.38	0.92	135	4.04	99.4	27.9

221	12.02	2.22	1.1	139.1	4.36	103.1	29.2
573.3	14.06	2.37	0.95	130.4	2.7	90.3	27.9
435.5	7.94	2.33	0.99	136	4.03	102.7	24.6
205.4	13.17	2.21	1.05	135.5	3.85	102.7	23.7
282	8.78	2.32	0.96	135.9	3.85	100.7	25.9
294.7	7.81	2.4	1.06	139.6	4.24	103.1	29.2
236.7	15.45	2.3	1.1	134	4.33	97.9	28.3
342.2	18.85	2.4	1.21	134.2	4.43	105.4	25.1
265	10.53	2.3	1.05	134.4	3.54	101.6	24.2
491.2	13.6	2.45	1.2	137.5	3.74	102.6	21.7
257.4	17.81	2.21	0.98	126.5	3.94	95.4	25.5
193	18.93	2.23	1.17	133	3.85	101.1	24.5
491	5.24	2.11	1.15	139.9	4.1	101	32.4
260	8.46	2.2	0.89	137.3	3.91	105.1	23.9
301.7	12.71	2.52	1.18	137.7	4.59	101.1	28.8
219	20.57	2.41	1.37	137.9	3.59	101.6	22.7
550.2	22.14	2.27	1.32	131.5	4.91	96.5	32.1
243.8	8.5	2.32	0.94	136.6	4.2	102.4	26.1
308	9.75	2.28	1	136.7	4.28	101.1	25.6
264	6.09	2.25	0.86	136.8	3.92	106	27
540	17.45	2.39	1.35	138.3	3.89	101.1	24.9
445.7	19.07	2.32	1.15	132	4.24	99.3	24.5
454.9	9.2	2.35	1.18	137.3	3.89	103.5	26.7
289.9	11.88	2.28	1.11	135.5	4.34	101.6	26.2
226.1	10.72	2.34	1.23	136.2	4.43	99.1	33.1
378.3	13.2	2.3	1.04	136.3	3.74	101.9	25.2
228.3	13.37	2.31	1.29	133.9	4.03	100.9	26.2
298.2	18.95	2.34	0.83	136.6	4.03	101.2	29.1
345.1	10.7	2.35	1.06	138.3	3.92	105.9	26.2
336.6	11.85	2.31	0.93	135.4	4.46	103.1	27.7
228.4	20.26	2.33	1.22	134.2	4.11	102.5	26.3
335	7.45	2.25	1.06	142.6	3.83	106.4	26.4
238.4	16.58	2.27	1.12	135.1	4.12	102	22.1
275.4	9.91	2.35	1.23	137.7	4.09	104.1	28
352.1	18.3	2.36	1.07	137.4	4.51	104.3	25.9
288	4.36	2.07	1.52	140.1	3.67	108.3	21.1
261.3	9.13	2.22	0.79	136.4	3.81	102.3	28.2
435	10	2.28	1.19	139.4	3.69	101.8	25.3
299.7	14.12	2.38	1.13	135.4	4.12	102.3	25.7
209	9.28	2.28	1.09	137.6	4.14	107.2	26.7
350.8	17.24	2.49	1.25	132.6	4.33	90.6	22.8
306.1	24.89	2.37	1.01	131.9	3.88	100.8	23.3
431.5	17.87	2.45	1.17	131.8	4.58	94.4	29.4
367.1	20.38	2.31	0.93	137.9	4.08	102	28.6
279	9.74	2.31	1.06	139.7	3.97	108.4	21.8
422.8	12.65	2.32	1.05	134	4.51	101.5	25.6

288.9	10.58	2.25	1.01	134.2	3.9	101	26.3
228.1	21.19	2.31	1.13	137.4	3.76	99.8	31.2
259	15.99	2.32	0.84	136.2	4.22	97.8	28.3
297.4	11.38	2.32	0.98	138	3.9	102.7	28.9
231.4	13.32	2.43	0.95	133.8	4.61	102.2	24.4
260.3	9.66	2.31	1.4	136.1	4.03	104.8	25.6
312.2	15.05	2.47	1.5	137.1	3.83	102.5	26.2
239	6.67	2.2	0.85	143.3	3.99	108.9	24.7
241.3	13.41	2.35	1.03	135.1	4.54	100.5	25.8
354.2	14.53	2.3	1.02	134.2	4.1	102	27.5
287	8.1	2.23	1.05	141.2	4.08	106.2	28.3
212.5	28.02	2.19	1.02	125.9	4.41	94.3	26.7
505.2	14.9	2.34	1.02	133.6	3.86	97.2	26.2
374.8	10.94	2.35	1.33	136.5	3.93	101.9	26.8
436	4.73	2.23	1.37	140.9	3.24	104.2	26
429	13.84	2.42	1.17	136.3	4.18	101.1	21.8
235.2	12.07	2.25	0.92	135.9	3.99	102.5	26.3
264.7	22.04	2.22	0.83	132.7	4.72	99.2	30.6
337.3	12.02	2.47	1.34	140	4.5	104.2	28.8
297	6.67	2.37	1.2	139.1	3.8	103.4	24.6
323.9	4.79	2.35	1	139.8	3.95	104.1	28.4
354.8	12.75	2.16	1.11	136.4	3.73	104.8	26.9
292.4	15.7	2.37	0.91	134.4	3.95	99.7	27.1
203.8	16.35	2.26	1.12	134.7	4.75	100.8	27.7
361	7.76	2.11	0.93	138.4	3.88	106	21.4
313.8	15.01	2.31	1.03	135	4.29	98.7	35.6
367.9	11.4	2.18	0.75	135.3	3.8	102	29.6
307.5	20.66	2.15	0.72	134.1	3.95	102.2	26.4
221.8	10.29	2.39	1.17	139.3	4.47	103.1	35.2
279.9	8.84	2.32	0.95	138.9	3.59	100.5	35.2
370.3	6.42	2.17	0.94	139.8	3.62	106.5	30.4
232.5	17.27	2.28	0.79	131.7	4.37	97.7	27.5
381	10.19	2.25	1.21	142.1	4.14	104.2	26.3
462	10.16	2.23	1.1	138.7	3.68	104.6	29.6
378	14	2.28	1.15	128.4	4.68	95.4	28.9
387	6.15	2.06	0.97	143.6	3.51	108.7	27.5
304.7	9.49	2.5	1.24	136.1	3.58	98.2	29.1
338.4	9.82	2.35	1.26	137.5	4.42	102.9	24.4
291	12.81	2.29	1.06	136.4	4.36	99.7	29.4
211.8	12.1	2.27	1.03	140.6	3.94	109	24.1
557	5.21	2.06	0.88	141.5	3.89	112.1	21.8
264.3	8.99	2.36	1.12	136.9	3.91	103.5	24.8
200.9	19.3	2.21	1.18	132.4	4.2	102	23.3
266.1	18.92	2.44	1.2	137.3	4.28	104.1	27
223.8	18.05	2.25	1.24	135	4.22	103.1	26.4
308.1	9.36	2.45	1.23	139.6	4.1	105.4	23.7

370.2	16.89	2.53	1.47	138.6	4.14	99.6	30
594.9	18.09	2.43	1.22	136.5	4.24	100.8	22
395.8	23.86	2.38	1.11	132.4	4.05	97.3	25.1
269.2	13.3	2.37	1.05	134.7	4.26	102.1	28.3
286.4	7.74	2.39	1.21	144.3	4.15	108.8	26
224	8.18	2.32	1.14	141.5	4	107.1	24.7
400	6.6	2.12	1.38	139.7	3.98	104.3	25.8
312.2	15.17	2.46	0.89	138.9	4.27	103.8	25.1
465.7	9.77	2.41	1.1	134.7	3.89	98.6	27.4
224.1	18.42	2.28	1.33	129.7	4.34	98	25
368.2	9.28	2.46	1.47	137.6	4.14	103.2	25.3
266	7.14	2.25	1.3	144.5	4.3	105.8	29.3
418	17.44	2.34	0.99	134.3	4.54	102.7	24.5
230.7	10.08	2.48	1.26	137.5	4.59	100.9	29.7
318.5	7.87	2.36	1.1	137.1	4.04	102.3	27.2
340.1	9.98	2.39	0.95	137	3.97	101.1	27.5
362	5.54	2.15	1.15	141.1	3.72	107.4	23.6
341.8	9.29	2.45	1.3	133.9	4.06	100.6	25.7
253.1	6.03	2.34	1.07	139	4.4	105.7	29.6
375.2	17.44	2.44	1.33	132.6	4.96	93.7	31.9
151.6	28.75	2.22	1.03	128.2	3.68	98.3	23.8
324	6.63	2.48	1.12	137.6	4.47	105	23.9
282.9	14.13	2.3	1.32	132.5	4.07	99.9	22.4
401.5	5.46	2.21	1.21	139.6	4.05	106.9	26.6
443	11.87	2.28	1.13	138	3.99	106.3	22
346.8	15.15	2.18	1.13	138.2	3.59	105.1	23.5
346.7	8.16	2.41	1.37	139.4	4.08	104.7	25.6
334	7.34	2.33	1.41	137.6	4.35	104.2	24.2
334	7.89	2.38	1.1	141.4	3.95	106.9	26.2
469	6.08	2.45	1.37	139.5	4.08	105	26.6
333	10.28	2.37	0.85	138.6	4.27	101.9	27.7
196	9.65	2.34	0.89	138.8	4.39	100.8	27.9
259.4	10.41	2.38	1.32	135.9	4.01	100.3	29.6
308.3	9.27	2.3	1.1	137	3.95	102.9	27.8
327	11.51	2.3	1.06	137.5	4.23	103.8	29
360.7	10.84	2.39	1.03	136.5	4.42	101.8	29.4
201	12.29	2.4	1.18	138	4.76	103.8	30.7
298	15.8	2.38	0.86	133.8	4.26	98.2	22.4
225.1	18.08	2.35	1.28	136.2	4.3	100.9	28.8
212.4	14.94	2.37	1.01	134.9	4.15	100.3	27
274.5	12.11	2.25	1	137.2	3.99	103.8	26.6
507.1	11.06	2.34	1.14	135.6	4.53	103.8	25.6
225.6	15.64	2.17	1.27	134.3	3.74	99.8	28.6
262.8	15.57	2.33	1.17	134.2	4.04	101.6	22.6
157.5	9.67	2.3	1.08	138.7	4.18	104.3	29.1
331.1	18.52	2.3	0.91	133.5	4.2	102.5	23.9

447.7	24.61	2.28	1.32	129.2	6.01	100.3	20.8
269.8	23.94	2.37	1.26	135.3	3.62	100.4	27.6
333.3	10.01	2.25	1.15	137.6	4.14	103.5	26.3
98	10.44	2.39	1.18	138.9	3.73	98.7	26.1
241	3.35	2.22	1.1	143.9	3.73	110.2	26.2
336.5	8.34	2.34	1.22	135.5	4.34	100.4	26.3
373	3.88	2.26	1.3	143	4.59	107.5	24.6
391.5	16.37	2.33	1.11	135.2	3.7	99.8	22
413	15.38	2.42	1.16	137.2	3.92	102	24.2
396.2	11.5	2.2	1.34	137.8	4	102.9	25.5
303	17.92	2.33	0.92	134.6	2.96	93.4	27.4
247.4	10.55	2.18	1.04	138.8	4.6	104.3	28.6
327.5	8.66	2.32	1.19	138.2	3.9	105.1	25.3
421.4	14.03	2.31	1.06	134.7	4.5	101	27
303	11.48	2.31	1.03	138.3	4.25	104.9	23.9
381	9.85	2.25	0.75	143.7	4.19	104.1	23.8
293.4	7.74	2.37	1.4	137.7	3.97	103.4	25.5
242	3.21	2.19	1.24	143.5	3.9	104.5	27.6
255	14.26	2.43	1.07	142.3	5.01	102.5	24.6
201.7	15.37	2.29	1.14	135.1	4.18	98.4	26.5
370.2	15	2.26	0.96	136.5	3.2	100.6	25.4
308.8	12.87	2.28	1.16	133.7	4.67	103.4	21.7
370.2	6.25	2.36	1.22	139.8	4.42	104.3	25.5
236.9	21.84	2.25	0.8	132.6	3.69	95.7	27.6
239.9	14.04	2.46	1.54	136.7	4.44	102.8	26.3
215.4	15.69	2.27	1.16	135.8	4.34	101	26.9
374.1	17.48	2.34	1.32	135.2	3.51	95.8	19.7
236.2	9.39	2.31	1.11	137.8	4.38	101.3	30.6
329.7	9.69	2.17	1.35	136.7	3.7	103.2	23
201.8	15.13	2.24	1.18	136.1	4.22	102.5	27.3
370.8	11.91	2.24	0.93	136.2	4.84	104.7	26.2
344	6.38	2.19	1.26	142.1	3.71	104.9	27.3
385	11.24	2.3	1.13	147.9	3.91	106.9	22.7
304.2	5.31	2.39	1.18	140.4	4.27	107	26
281	9.4	2.36	0.99	139.3	5.41	110.1	20.4
233.3	24.19	2.31	0.94	132	4.39	99	24.4
321	10.41	2.35	0.97	141.3	4.43	103.6	23.9
250.3	8.55	2.49	1.37	136.6	3.31	101.8	24.1
359.4	13.87	2.44	1	135.1	3.88	100.8	26.1
397.4	32.26	2.34	1.29	127.9	4.02	92.1	26.5
429.7	15.02	2.36	1.03	135.6	4.7	100.4	25.6
329.3	12.61	2.41	1.23	136.4	4.11	101.3	27.3
294.5	8.12	2.33	1.19	137.1	4.26	103.8	24
464.8	10.81	2.21	1.11	136.4	3.65	104	25
216.3	6.51	2.17	1.41	138.1	3.62	105.6	27.9
241.4	18.36	2.16	0.99	134	4.42	99.5	29.8

393	8.49	2.46	1.25	138.9	3.64	102.5	24.1
343	17.12	2.29	1.11	136.5	4.38	98.1	27.9
204.4	15.9	2.27	1	133.6	3.96	100.5	22.1
302.8	9.17	2.24	1.02	139.4	4.02	103.9	27.4
224.9	9.88	2.38	0.93	133.1	4.24	99.9	25.1
366.9	5.94	2.38	1.26	139.3	4.48	102.6	30.7
393.9	18.45	2.23	1.48	131.3	4.18	97.4	27.4
228.7	5.76	2.2	1.09	135	3.72	103.4	20.7
183.5	4.91	2.29	1.15	138	4.21	103.3	24.7
124	10.33	2.24	1.25	138.3	4.07	100.2	26.9
208.3	5.18	2.34	1.27	143.4	4.31	106	29.4
356.9	11.19	2.31	1.18	137.5	4.69	102.8	26.4
224.4	9.62	2.29	1.19	135.2	4.61	104.3	26.9
328.2	6.93	2.32	1.01	136.2	4.52	104.3	26.4
225	11.09	2.25	1.38	143.4	3.97	107.4	26.4
256	6.52	2.31	1.06	140.6	4.33	106.8	26
269.1	6.37	2.42	1.14	139.5	4.34	102.3	31.7
396.8	15.17	2.33	1.04	132.9	4.57	96.7	29.6
296.2	9.57	2.25	1.09	135.7	4.49	101.1	29.8
284.1	7.52	2.31	1.19	137.7	4.28	105.6	26.4
271.7	9.91	2.26	1.26	138.2	3.78	103.8	27
224	15.22	2.4	0.99	136.3	4.2	101.7	22.3
342	23.52	2.24	1.55	134.1	4.62	102.6	22.9
316.1	8.01	2.22	0.92	134.5	3.93	102.7	23.4
179.9	16.4	2.26	1.29	134.7	3.75	97.9	27.3
118.4	15.16	2.27	0.91	135.3	4.13	100.3	26.4
282.2	7.28	2.31	1.29	138.5	3.8	105.1	25.3
214.5	15.71	2.38	1.15	136.5	4.3	99.8	27
325.5	11.86	2.33	1.23	137.5	4.09	102.4	29
236.4	11.55	2.26	1.11	139	4.33	104.1	29.1
257	22.43	2.51	1.34	136	4.89	99.9	26.7
301.8	10.43	2.25	1.13	139.6	3.09	101.8	28.1
620.1	8.67	2.34	1.31	135.4	4.28	100.8	25.9
159.7	14.35	2.46	1.36	134.5	4.1	100.5	22.6
334.6	7.48	2.22	1.26	138.6	4.58	107.4	22.3
286.7	18.49	2.11	0.92	133.5	3.84	99.1	22
177.8	8.72	2.16	1.14	135.5	4.46	103.1	24.4
364	18.93	2.16	0.92	131.4	3.45	98.7	23.1
198.1	18.78	2.4	1.16	136.8	4.36	101.2	26.4
275	8.27	2.29	1.19	142.1	3.95	104.3	28.6
343.4	11.48	2.33	0.93	138.6	4.5	101	23.9
242.2	17.16	2.52	1.36	137.7	4.39	101.3	30.6
258	7.36	2.33	1.11	136.4	4.79	101.2	28.8
370.6	8.93	2.39	1.34	136.1	4.47	98.6	26.9
273.5	30.81	2.3	1.43	131.7	4.59	100.9	21.1
376.5	12.86	2.6	1.13	134	4.06	96.6	27.7

369.8	11.19	2.34	1.07	138.2	4.78	106.8	23.2
430	5.33	2.32	1.23	141.7	4.33	106.3	25.8
207.8	12.44	2.28	0.94	140.2	4.53	99.4	32.2
421	10.45	2.29	1.41	141.4	4.67	107.2	22.6
271.4	13.75	2.28	0.97	140.7	4.07	102.6	28.1
185.3	8.93	2.36	1.45	139.8	4.08	103.6	28.6
303.6	6.41	2.08	0.79	133.6	3.85	100.5	23.8
239.7	11.43	2.32	1.1	136.8	4.02	102.7	28
227	5.31	2.23	0.92	140.4	3.84	104.5	27.2
314.7	12.46	2.36	0.97	140.5	4.49	103.5	31.4
389.3	12.9	2.46	1.45	136	4.63	97.4	30.7
444.4	12.79	2.24	1.29	137.7	4.24	102.9	24.6
369	11.83	2.41	1.03	134.2	4.27	99.2	26.6
298.5	13.78	2.2	0.92	133.7	4.38	102.1	27.4
350	4.1	2.27	1.08	139.6	4.3	105.2	25
299	6.64	2.4	1.31	141.5	4.89	104.7	23.1
404	9.91	2.39	1.33	138.6	3.82	103.4	21.4
281.5	11.65	2.31	1.17	136.9	4.26	103.1	28.1
321.5	12.09	2.33	1.75	138.9	3.94	97.8	33.6
390	9.44	2.38	1.31	142.7	3.91	105.8	25.2
185.8	15.65	2.21	0.85	138	3.62	106.4	24.7
383	10.56	2.26	1.32	137.6	3.85	107.6	22.4
267.3	14.2	2.26	1.11	135.6	3.77	99.4	28.7
312.2	15.89	2.29	1.24	134.3	3.63	100.6	24
299	7.57	2.37	1.4	141.5	4.01	106	21.9
340.1	26	2.08	1.15	132.2	4.29	98.8	28.2
285.5	13.07	2.21	1.16	132.6	4.07	102.5	21.6
315	12.19	2.28	0.99	138.8	3.91	103.4	29
582	19.16	2.42	1.19	133.4	5.78	98.8	23.4
202	19.05	2.33	0.93	132.3	4.78	95.6	24.5
281	13.77	2.35	1.25	133.7	4.7	98.3	29.3
352.9	11.31	2.32	1.22	131.9	4.22	98.8	24.3
170.2	15.37	2.3	1.22	133.6	4.08	98.8	26.6
219.5	10.26	2.18	1.1	134.4	4.03	101.9	26.1
367	8.92	2.34	0.96	139.1	4.03	100.7	27.5
241	3.35	2.22	1.1	143.9	3.73	110.2	26.2
454.6	14.65	2.6	1.21	133.1	4.53	95.8	26.6
205.7	9.53	2.26	1.05	136.2	3.81	102.8	26.3
210.5	9.79	2.4	0.96	139.2	4.01	103.3	29.6
203.2	9.54	2.4	1.09	136.5	4.27	103.3	24
202.6	10.74	2.33	1.03	136.3	4.21	101.6	27
438.7	11.58	2.21	1.02	137.9	4	103.1	29.2
306.1	11.82	2.29	0.97	133.7	4.09	100.7	27.8
427.9	12.24	2.42	1.23	138.8	4.4	101.8	25.7
257.3	24.57	2.18	1.1	128.9	4.3	92	29.1
176	5.69	2.34	1.28	141.8	4.26	103.2	28.9

230.6	9.65	2.25	1.81	134.8	3.86	101.3	26.3
237.9	14.38	2.29	0.92	133.4	4.21	100	24.9
203	10.85	2.32	1.36	141.9	4.55	103.4	27.3
309.4	28.27	2.21	1.06	132.2	4.24	97.6	29
421.3	10.86	2.26	1.16	137.6	4.31	105.2	20.9
351.2	9.85	2.51	0.87	134.9	4.27	98.6	31.9
595.5	14.55	2.46	1.19	133.7	4.36	100.1	25.5
306.1	11.82	2.29	0.97	133.7	4.09	100.7	27.8
290	11.68	2.34	1.22	135.4	4.23	100.1	27.4
291.5	10.58	2.4	1.68	135.3	4.28	100.6	24.8
283	9.25	2.35	1	138.8	4.27	102.2	27.2
324.2	13.37	2.36	1.51	137	4.1	105.4	23.1
366.4	7.78	2.39	1.33	138.6	4.12	101.7	28.7
107	16.28	2.28	0.97	138.7	4.17	101.2	27.1
275.4	12.07	2.25	1.13	135.7	4.29	103.2	24.1
263	12.41	2.4	1.07	138.3	4.9	101.9	28.1
645	31.92	2.37	1.22	125.2	3.98	87	27.1
395	10.97	2.39	1.15	138.3	4.49	104.6	24.3
270.4	10.65	2.57	1.17	134.4	4.56	101.3	26.6
301	8.98	2.44	0.91	133.6	3.96	96.1	25.7
340	8.08	2.34	1.06	134.3	4.52	105.3	21.1
293.7	8.83	2.28	1.43	134.9	4.29	100.2	26.7
235.6	10.46	2.35	1.04	139.5	4.16	103.6	28.7
403.6	10	2.25	1.21	136.6	4.47	100.2	26.6
285	9.1	2.45	1.22	139.9	5.02	100.7	27.1
344	9.36	2.29	1.09	133.8	4.67	101	23.9
448.2	12.29	2.43	1.28	135.3	4.28	99.6	27.6
162	13.42	2.18	0.87	137.1	3.96	106.9	25.3
330.3	14.98	2.23	1.11	134.8	4.38	101.4	25.9
254	10.21	2.46	1.41	139	4.37	100.2	26.8
330	7.43	2.29	1.06	140.6	3.88	107.1	22
401.1	20.74	2.24	1.42	133.6	4.29	101.5	22.1
271.2	16.47	2.05	0.72	132.5	3.47	104.4	19.1
366.1	6.12	2.29	1.09	138.1	4.48	106.1	24.8
373	8.38	2.41	1.08	142.2	4.42	105.5	26.9
381	6.56	2.11	0.96	136.4	4.05	105.5	24.5
497.9	13.36	2.31	1.27	134.9	3.23	99.6	24.4
570.1	10.63	2.28	1.11	136	3.84	99	27.1
431	19.53	2.55	1.19	138.9	4.45	97.6	28.4
371.2	7.2	2.36	1.39	137.8	3.93	103.9	23
250	11.01	2.3	1.43	139.6	4.5	105.6	29.3
193	12.54	2.42	1.3	134.4	3.76	102.3	30.1
247.2	9.12	2.35	1.46	138.3	3.73	102.1	25.7
354	10.88	2.29	1.19	141.1	4.59	105.3	26.3
224	7.27	2.3	1.05	142.2	3.77	106.6	26.3
303	10.25	2.28	1.08	136.1	3.77	103.5	23.1

429	9.27	2.36	0.91	136.2	4.21	104.5	23.2
142.1	7.72	2.26	1.08	139.1	4.06	106.4	27
255	14.35	2.28	1.19	138.3	4.22	104.3	25.5
456	15.41	2.53	1.14	139.8	4.99	98.2	26.7
384	5.6	2.38	1.2	138.8	3.59	103.6	23.9
424.7	16.03	2.37	1.11	138.2	4.77	102.8	23.1
352.7	20.95	2.19	1.21	138.2	4.42	103	26.2
208.2	7.81	2.24	1.18	141.8	4.22	107.2	24.9
246.5	16.66	2.29	1.18	139.1	4.13	104.5	18.3
260	6.42	2.24	1.12	138.2	3.73	103.3	25.1
285	10.39	2.3	1.18	134.6	4.15	101	25.6
388.6	13.23	2.28	1.05	135.3	3.95	101.7	23.6
177.6	14.61	2.3	1.21	136.6	4.38	103.1	25.8
276.7	7.5	2.55	0.97	139.6	3.41	104.9	24.8
359.7	4.9	2.2	1.28	139.5	4.33	107.6	23.2
339.3	17.43	2.31	1.35	134.2	4.12	97.9	25.8
182	8.68	2.34	1.26	141.5	4.33	104.5	29.1
290.6	5.8	2.3	1.14	139.4	3.86	104.4	29
449.7	19.26	2.39	0.98	136.7	4.45	103.1	20.4
300.7	14.42	2.33	1.08	138	4.09	105.8	21.5
561	7.46	2.43	1.05	137.2	3.92	100	24.9

TCH	TG	HDL-C	LDH-C	APOAI	APOB	APOa	ADA
5.36	2.35	1.08	3.02	1.51	1.1	0.644	15.6
3.57	1.54	0.76	1.87	1.28	0.68	0.405	12
4.21	2.19	0.73	2.16	1.28	0.73	0.027	11.1
5.68	1.85	0.9	3.23	1.49	1.05	0.037	11.6
4.39	1.33	0.99	2.59	1.41	0.85	0.088	12.2
5.04	1.05	1.31	2.94	1.65	0.95	0.653	9.6
2.74	1.06	0.56	1.33	1.03	0.59	0.066	13.5
4.62	1.26	1.37	2.19	1.69	0.8	0.112	13.4
3.42	2.82	0.83	1.54	1.38	0.64	0.829	8.1
4.64	1.33	0.72	2.72	1.13	0.95	0.15	13.6
4.26	5.34	0.69	1.84	1.25	0.74	0.006	10.7
4.92	0.53	1.89	2.43	1.98	0.69	0.116	13.5
5.66	2.48	0.7	3.33	1.14	1.18	0.006	13.3
4.11	1.16	1.58	1.74	1.87	0.54	0.005	9.7
2.52	1.85	0.91	0.89	1.58	0.47	0.178	24.9
4.93	3.92	1.01	2.53	1.52	0.8	0.025	8
8.33	1.9	1.13	5.16	1.45	1.64	0.537	13.1
5.93	1.64	1.1	3.62	1.52	1.2	0.204	17.2
2.58	1.18	0.81	1.24	1.32	0.47	0.103	11.6
5.99	1.76	1.03	3.63	1.41	1.17	0.074	17.8
3.71	1.7	0.77	2.01	1.32	0.68	0.007	15.4
6.66	10.1	0.98	2.62	1.54	0.91	0.014	20.3
6.3	4.29	0.92	3.04	1.58	1.1	0.006	12.4
4.53	1.73	0.71	2.47	1.17	0.86	0.198	8.8
3.6	1.38	1.08	1.77	1.51	0.63	0.29	15.5
4.93	1.38	0.89	2.83	1.3	0.91	0.094	10.2
4.98	2.03	1.03	2.82	1.61	0.91	0.123	14.6
5.31	1.22	0.93	3.22	1.32	1.05	1.196	20.9
4.48	2.14	0.92	2.43	1.45	0.88	0.248	51.2
4.59	0.87	1.21	2.45	1.65	0.81	0.042	15.2
4.41	1.35	0.69	2.73	1.13	0.91	0.056	16.8
3.52	1.17	1	1.7	1.47	0.57	0.043	12.1
4.16	1.35	0.63	2.54	0.93	0.82	0.25	12.6
6.02	1.34	1.02	3.43	1.47	1.01	0.075	20.3
5.1	0.99	1.23	2.85	1.66	0.88	0.055	15.1
6.05	1.87	1.02	3.38	1.6	1.13	0.051	10.1
4.48	0.89	1.11	2.16	1.39	0.71	0.35	25.2
4.25	10.51	0.8	1.37	1.45	0.5	0.125	21.5
4.48	1.21	0.75	2.71	1.13	0.94	0.198	16
6.51	2.81	1.67	2.99	2.27	0.99	0.013	11.3
4.02	1.06	0.9	2.08	1.19	0.7	0.106	9.3
4.7	2.18	1.19	2.98	1.3	0.98	0.368	14.9
4.46	2.65	0.8	2.44	1.21	0.79	0.079	18.5
5.12	3.65	0.83	2.56	1.37	0.89	0.051	16.6
4.33	4.61	0.58	2.53	1.08	0.85	0.017	12.8

4.19	0.6	1.28	2.05	1.41	0.63	0.033	13.8
8.31	8.68	0.56	3.55	1	1.1	0.03	13.5
6.06	1.16	0.94	3.81	1.24	1.27	0.289	10.7
4.08	2.82	0.59	2.51	0.99	0.88	0.121	7.2
4.64	1.07	1.25	2.56	1.61	0.87	0.109	10.5
2.6	0.33	1.09	1.07	1.5	0.32	0.007	15.9
6.66	0.99	2.42	3.21	2.32	0.97	0.11	9.2
5.51	7.36	0.57	2.4	0.97	0.85	0.189	10.5
5.49	6.26	0.9	2.36	1.47	0.92	0.025	8
5.57	5.08	0.8	2.87	1.27	1.09	0.08	18.1
3.06	0.87	1.01	1.68	1.13	0.54	0.074	8.8
6.03	0.67	2.04	3.04	2.1	0.85	0.28	9.4
3.24	1.5	0.46	1.89	0.83	0.69	0.074	10.3
2.55	0.62	0.8	1.26	1.19	0.5	0.255	14.1
6.32	2.26	1.22	3.51	1.59	1.19	0.089	13.4
6.36	1.09	1.15	3.91	1.61	1.32	0.736	17.5
3.18	1.63	0.8	1.53	1.34	0.58	0.088	21
3.7	1.71	1	1.66	1.47	0.66	0.68	8.4
3.75	2.57	0.77	1.8	1.35	0.72	0.193	9.9
3.34	0.64	1.08	1.56	1.37	0.51	0.007	14.7
7.25	5.85	0.89	3.56	1.46	1.29	0.002	13
5.93	10.21	0.9	2.51	1.3	0.98	0.001	13.9
8.34	23.54	1.37	1.22	1.05	0.81	30	11.8
5.88	0.62	1.56	3.2	1.7	0.9	0.074	12.9
7.43	1.39	1.85	4.25	2.13	1.29	0.153	17.7
4.61	2.78	0.81	2.58	1.31	0.92	0.095	15.8
5.72	3.45	1.02	3.16	1.64	1.04	0.354	14.6
5.17	4.31	0.91	2.58	1.48	1	0.402	12.5
6.86	10.28	0.87	2.55	1.49	1	0.07	12.2
5.07	1.83	1.05	3.03	1.36	1.02	0.385	10
3.55	0.81	0.89	1.9	1.31	0.63	0.02	10.6
3.19	0.97	0.8	1.93	1.09	0.69	0.06	9.2
3.45	0.9	0.52	2.17	0.77	0.77	0.002	18.1
5.87	1.18	1.07	3.58	1.44	1.16	0.619	14.3
5.01	1.66	1.04	3.1	1.37	1.05	0.044	13.1
3.22	0.81	0.68	1.98	1.06	0.64	0.226	7.6
4.25	3.75	0.73	2.3	1.23	0.78	0.136	15
4.69	2.51	0.63	2.67	0.99	1.02	0.066	13.6
5.34	1.81	0.73	3.34	1.08	1.13	0.093	13.9
3.28	0.58	1.17	1.71	1.49	0.6	0.125	14.4
6.37	1.68	0.72	4.04	1.19	1.37	0.288	22
5.23	1.72	0.71	3.2	1.12	1.06	0.033	10.1
2.59	2.25	0.57	1.42	1.04	0.54	0.003	26.5
5.86	2.56	0.66	3.92	1.1	1.34	0.127	17.4
4.37	2.27	0.62	2.81	1.04	1.03	0.245	11.4
5.3	4.06	0.82	2.86	1.25	0.99	0.01	7.9

4.04	1.73	0.89	2.44	1.34	0.87	0.04	17.2
3.91	1.57	0.85	2.24	1.22	0.8	0.133	20
5.23	2.69	0.81	3.07	1.22	1.01	0.002	14.3
6.41	1.4	1.38	3.75	1.59	1.19	0.015	14.3
6.29	1.32	1.15	3.87	1.5	1.22	0.206	15.2
3.23	3.36	0.65	1.61	1.26	0.63	0.06	16
4.79	1.36	1.1	2.81	1.44	0.91	0.237	21.5
4.5	1.1	0.97	2.68	1.27	0.92	0.194	9.4
3.64	1.81	0.83	1.72	1.42	0.62	0.006	23
4.16	3.91	0.78	2.15	1.31	0.81	0.022	12.3
4.01	0.97	0.76	2.46	1.09	0.79	0.143	8.5
4.32	1.75	0.68	2.61	0.91	0.92	0.058	19.2
3.59	2.22	0.66	2.1	1.11	0.76	0.036	8.7
4.63	2.99	0.9	2.52	1.25	0.86	0.03	15.8
4.24	1.5	1.15	2.22	1.46	0.84	0.094	7.5
6.69	2.27	1.23	3.79	1.75	1.2	0.018	8.7
5.81	2.71	1.02	3.42	1.39	1.13	0.023	14.2
3.69	2.04	0.99	1.83	1.58	0.66	0.156	10.2
5.27	0.88	1.01	3.15	1.14	0.84	0.178	12.5
3.94	2.28	1.42	1.7	1.89	0.67	0.006	9.8
3.28	1.41	0.69	1.87	1.13	0.7	0.028	10.4
3.76	1.31	0.78	2.08	1.05	0.72	0.167	16
4.94	1.73	0.99	2.93	1.43	0.97	0.331	17.4
4.41	0.74	1.05	2.37	1.32	0.74	0.162	11.8
5.53	1.69	1.34	3.09	1.84	1	0.084	9.1
4.22	0.61	1.07	2.3	1.4	0.76	0.54	13.2
2.67	1.38	0.45	1.5	0.84	0.6	0.034	12.6
6.11	1.44	0.88	3.9	1.29	1.26	0.132	18.4
5.16	1.02	1.61	2.8	2.08	0.9	0.087	11
4.98	0.9	1.51	2.7	1.91	0.81	0.002	10.1
4.81	4.89	0.79	2.15	1.45	0.85	0.023	8
3.04	0.43	0.71	1.79	1.08	0.62	0.153	23.2
4.09	1.32	0.81	2.34	1.07	0.75	0.032	12.1
4.07	4.69	0.7	2.03	1.09	0.82	0.113	10.5
4.82	2.89	0.78	2.71	1.13	1.08	0.096	35.3
2.69	1.36	0.54	1.66	1.05	0.59	0.334	9.9
3.6	0.89	1.01	1.63	1.36	0.5	0.281	13.1
5.69	1.65	1.21	2.99	1.65	1	0.092	15.1
4.43	2.19	0.69	2.4	0.97	0.75	0.029	10.3
5.44	1.17	1.47	3.08	1.34	0.8	0.038	12.5
3.13	2.23	0.67	1.58	1.2	0.56	0.069	11.5
3.11	0.95	0.85	1.55	1.44	0.56	0.056	9.6
7.46	1.29	2.02	4.2	2.17	1.26	0.169	12.6
4.03	1.95	0.81	2.11	1.11	0.8	0.113	27.5
4.83	1.2	1.02	2.83	1.45	0.91	0.09	21.1
4	1.27	0.97	2.09	1.4	0.77	0.218	21.6

4.24	4.05	0.96	2.28	1.57	0.9	0.082	28.4
5.48	3.79	0.81	3.08	1.38	1.16	0.014	15
4.38	1.89	0.66	2.79	0.98	1.02	0.072	9
5.27	2.77	0.97	3.26	1.3	1.01	0.022	7.9
4.43	1.72	0.76	2.75	1.25	0.89	0.115	6.3
5.36	1.12	1.08	3.14	1.35	0.98	0.189	6.2
4.05	2.43	0.68	2.25	1.07	0.77	0.519	11
4.57	0.95	0.87	2.94	1.36	0.93	0.108	13
5.8	2.77	0.74	3.69	1.18	1.26	0.403	7.8
4.76	1.12	1.02	2.87	1.43	0.91	0.506	22.9
4.79	2.19	1.12	2.57	1.62	0.86	0.135	11.5
5.58	1.49	0.98	4.24	1.03	1.1	0.08	6.8
7.76	6.82	1.03	3.52	1.53	1.1	0.074	19.6
5.57	1.15	1.4	3.04	1.68	0.92	0.003	11.6
5.65	1.91	0.96	3.36	1.4	1.01	0.143	13.2
4.68	1.91	1.06	2.46	1.57	0.75	0.024	12.1
3.79	1.02	1.34	1.82	1.67	0.64	0.289	20.6
5.69	1	1.3	3.37	1.52	0.99	0.41	9.1
4.84	0.75	1.47	2.53	1.45	0.74	0.143	11.6
5.65	1.81	0.83	3.42	1.32	1.1	1.237	16.2
2.66	0.45	1.17	1.29	1.48	0.37	0.008	14
5.11	1.69	1.08	3.39	1.26	1.29	0.076	8.9
5.66	1.26	1.69	3.54	1.7	1.32	0.2	15.1
3.23	1.19	1.2	1.73	1.46	0.56	0.095	11.7
5.62	6.25	0.71	2.37	1.37	0.92	0.162	15.2
4.08	7.33	0.78	1.55	1.26	0.66	0.008	6.1
4.98	0.79	0.9	2.91	1.26	0.87	0.06	6.3
4.82	1.29	1.07	2.7	1.42	0.79	0.24	14.3
3.53	1.51	0.96	1.75	1.46	0.59	0.146	4.9
6.62	2.08	0.99	4.12	1.3	1.26	0.04	15
5.1	5.9	1.02	2.22	1.26	0.82	0.003	4
4.51	2.9	0.98	2.34	1.44	0.83	0.056	6.9
4.06	2.91	0.78	2.2	1.41	0.74	0.012	13.3
3.32	1.33	1.14	1.61	1.55	0.6	0.549	5.9
4.82	2.49	0.97	2.65	1.36	0.82	0.013	8.6
3.79	1.21	0.85	2.2	1.13	0.7	0.091	10.8
5.44	2.05	1.21	2.84	1.67	1.02	1.538	12.4
6.78	7.99	0.8	2.95	1.39	0.92	0.003	12.6
4.06	2.93	0.73	2.2	1.18	0.78	0.045	13.9
6.06	2.32	0.92	3.73	1.34	1.05	0.055	10.7
4.68	1.81	0.73	2.89	1.18	0.92	0.464	10.5
3.75	2.23	0.66	2.13	1.24	0.79	0.145	14
5.96	1.35	1.03	3.62	1.42	1.03	0.42	17.8
4.23	6.18	0.64	1.68	1.01	0.72	0.174	26.6
4.57	1.02	1.21	2.26	1.6	0.78	0.095	13.2
2.7	1.93	0.67	1.25	1.15	0.52	0.093	12.3

5.13	6.62	0.7	2.27	1.15	0.88	0.013	22
3.59	1.54	0.76	1.94	1.16	0.7	0.014	13.2
4.02	1.24	0.79	2.11	1.3	0.78	0.109	8.5
4.94	1.79	1.22	2.51	1.68	0.85	0.042	11.2
4.13	0.64	1.28	1.95	1.47	0.67	0.26	11
3.85	1.19	0.85	2.12	1.41	0.78	0.039	16.6
4.23	1.23	0.69	2.45	1.15	0.89	0.719	14.1
5.17	6.43	0.63	2.29	1.16	0.87	0.235	15
5.55	1.01	0.91	3.39	1.23	1.14	0.528	13
3.73	1.83	0.87	2.03	1.53	0.67	0.004	12.4
2.98	4.7	0.75	1.07	1.3	0.56	0.011	38.9
3.87	1.39	0.81	1.89	1.36	0.7	0.165	11.2
4.47	1.33	0.8	2.65	1.21	0.92	0.13	8.9
4.26	2.01	0.74	2.53	1.13	0.89	0.239	19.1
4	0.73	0.96	2.37	1.34	0.72	0.041	9.1
4.5	0.96	1.04	2.65	1.26	0.7	0.024	6.4
2.75	1.17	0.8	1.57	1.05	0.56	0.06	6.8
3.89	0.58	1.52	1.69	1.7	0.54	0.159	9.7
4.81	1.12	1.05	2.7	1.6	0.91	0.085	14.3
3.87	1.58	0.61	2.21	1.07	0.78	0.033	16.2
3.59	0.93	1.69	1.41	0.61	0.025	0.025	10.7
4.74	1.17	0.92	2.87	1.24	0.86	0.012	11
5.8	1.5	0.83	3.51	1.2	1.06	0.036	16.7
4.06	1.28	0.84	2.4	1.18	0.78	0.103	22
4.17	0.86	0.92	2.24	1.18	0.71	0.04	16
5	1.41	0.94	2.85	1.36	0.95	0.115	23.1
4.64	2.66	0.9	2.53	1.28	0.94	0.034	16.6
3.94	1.18	0.82	2.2	1.23	0.7	0.039	15.3
4.49	2.08	0.81	2.7	1.28	0.99	0.201	10
4.15	0.63	0.87	2.42	1.26	0.79	0.651	19.2
6.75	1.8	1.16	4.06	1.38	1.36	0.607	17.2
5.16	1.76	1.02	3.16	1.27	0.96	0.363	6.4
4.43	2.24	0.84	2.52	1.23	0.97	0.453	22.2
2.42	0.94	0.69	1.28	1.14	0.5	0.038	6.8
6.1	1.99	1.1	3.46	1.34	1.16	0.014	10.2
4.43	2.49	0.81	2.46	1.34	0.86	0.175	15.6
5.47	1.88	0.7	3.31	1.21	1.06	0.032	7.4
3.38	1.31	0.94	1.76	1.38	0.67	0.071	8.3
6.17	6.11	0.94	2.81	1.61	1.01	0.013	11.5
6.05	1.73	0.76	3.68	1.16	1.17	0.491	18
4.96	2.39	0.69	2.85	1.03	0.91	0.052	9.8
4.9	1.31	0.92	3.2	1.32	1.04	0.743	18.3
3.33	3.22	0.71	1.5	1.33	0.6	0.169	12
4.18	2.17	0.61	2.67	0.91	0.8	0.066	8.4
3.93	0.78	1.09	2.21	1.36	0.69	0.22	13.5
4.69	1.82	0.69	2.92	1.25	0.98	0.199	12.2

8.4	3.72	1.62	5.39	1.82	1.72	1.038	12.2
5.23	2.03	0.67	2.53	1.19	0.74	0.096	28.1
5.34	2.05	1.11	3.26	1.56	1.03	0.235	12
3.06	1.96	0.7	1.75	1.18	0.63	0.058	8.1
3.58	0.65	0.94	1.86	1.31	0.57	0.1	14.7
4.82	1.1	1.01	2.84	1.42	0.86	0.213	9
4.32	1.55	1.02	2.1	1.48	0.81	1.149	29.3
4.27	1.27	2	1.36	1.98	0.59	0.082	21.6
5.81	0.59	1.27	3.42	1.62	0.99	0.077	9.3
3.67	1.92	0.78	2.06	1.29	0.72	0.044	16.3
4.51	1.34	1.12	2.4	1.58	0.77	0.148	8.7
4.75	0.85	1.11	2.95	1.48	0.89	0.407	8.6
2.87	1	0.63	1.5	1.2	0.58	0.381	18
4.87	1.04	1.02	2.79	1.46	0.85	0.389	9.4
2.56	1.34	0.71	1.24	1.28	0.53	0.567	7.8
3.34	0.96	0.77	1.74	1.22	0.55	0.166	9.6
2.72	0.78	1.04	1.16	1.44	0.36	0.146	7.3
5.44	1.51	1	3.36	1.5	0.97	0.232	14.1
5.48	1.4	0.96	3.38	1.36	1.05	0.112	13.5
5.02	2.6	0.96	2.73	1.52	0.91	0.243	8.7
4	1.3	1.19	2.12	1.66	0.66	0.328	14.3
4.36	1.11	1.03	2.54	1.38	0.76	0.361	17
9.99	3.15	1.36	5.79	1.77	1.9	1.354	29.4
3.73	1.24	0.77	2.05	1.09	0.67	0.03	15.3
4.33	1.41	0.67	2.74	1.02	0.88	0.328	17.3
5.94	1.14	1.22	3.57	1.56	1.1	0.065	7.8
5.13	1.33	1.08	3.03	1.51	0.86	0.119	10.9
5.26	1.15	1.05	3.16	1.33	1.03	0.02	12.6
6.66	1.14	1.38	3.99	1.67	1.2	0.071	16.4
3.61	0.83	1.15	2.01	1.59	0.61	0.242	14.1
3.06	1.53	0.81	1.64	1.45	0.58	0.013	24.9
5.37	1.86	1	3.22	1.41	1.05	0.052	20.6
3.84	0.81	1.08	2.02	1.42	0.65	0.014	11.8
4.01	1.24	1.04	2.26	1.4	0.85	1.036	17.4
4.1	0.86	0.76	2.61	1.15	0.82	0.082	14.4
5.08	1.21	1.17	3.03	1.45	0.97	0.163	15.7
4.8	1.11	1.23	2.58	1.56	0.78	0.042	12.7
6.54	2.76	0.82	4.12	1.2	1.41	0.098	18.1
4.59	1.18	0.95	2.61	1.38	0.89	0.227	8.2
4.67	1.23	0.63	2.88	1.12	0.88	0.272	12
4.22	0.89	1.01	2.37	1.43	0.85	0.262	10.8
3.99	1.36	1.26	1.9	1.67	0.59	0.015	9.7
4.66	2.47	0.65	2.66	1.08	0.84	0.071	15
8.13	1.17	1.78	4.66	2.24	1.32	0.004	10.3
3.68	1.49	0.69	2.25	1.1	0.84	0.87	15
5.48	2.65	0.87	3.15	1.28	1	0.005	13

4.03	1.21	0.88	2.23	1.27	0.78	0.058	22.6
4.66	2.45	0.73	2.63	1.24	1.05	0.088	15.9
6	1.67	1.08	3.57	1.57	1.13	0.268	12.7
6.57	1.28	1.56	4.67	1.69	1.66	0.292	16.5
4.62	1.11	1.13	2.63	1.54	0.82	0.086	7.8
5.11	1.76	1.04	2.92	1.52	0.9	0.034	16.1
3.14	1.04	0.82	1.54	1.1	0.56	0.062	12.9
4.75	2.01	0.98	2.82	1.42	0.92	0.08	18.1
4.33	0.73	0.99	2.21	1.49	0.82	0.203	7.8
3.08	0.8	1.08	1.65	1.51	0.56	0.149	11.3
4.56	4.96	0.71	2.42	1.13	0.82	0.047	12.9
4.87	2.82	1.14	3.48	1.33	1.31	0.052	6.1
4.54	2.24	0.78	2.87	1.22	0.98	0.018	11.8
3.16	0.89	1	1.7	1.33	0.54	0.001	10.2
4.4	2.03	0.89	2.76	1.33	0.95	0.256	8.8
4.11	1.13	1.29	2.33	1.74	0.75	0.142	11.6
3.08	3.61	0.68	1.72	1.29	0.61	0.075	16.6
6.46	2.03	1.26	3.89	1.79	1.28	0.041	9.9
5.99	1.57	0.76	3.71	1.17	1.28	0.279	11.2
4.02	2.63	1.14	2.01	1.81	0.65	0.328	19.3
3.42	1.35	1.16	1.57	1.5	0.524	63.2	19.1
3.39	2.08	0.7	1.87	1.19	0.68	0.211	7.5
5.95	2.58	1.1	3.49	1.63	1.05	0.005	11.7
4.81	0.86	0.82	2.76	1.24	0.92	0.222	11.4
3.84	1.59	0.85	2.38	1.36	0.75	0.045	5.8
5.8	0.76	0.86	3.7	1.13	1.15	0.299	14
3.8	0.78	1.34	1.9	1.6	0.65	0.235	10.8
2.75	1.24	0.78	1.55	1.28	0.58	0.274	13.1
4.19	1.78	0.82	2.56	1.3	0.88	0.01	15.4
4.48	1.56	0.74	2.86	1.14	0.93	0.136	26
6.05	3.37	0.9	3.4	1.39	1.17	0.16	21.4
4.81	3.36	0.93	2.73	1.47	0.94	0.063	19.7
5.36	1.06	1.1	3.39	1.51	1.07	0.196	15.9
3.2	0.98	0.95	1.7	1.47	0.68	0.799	13.6
3.47	1.48	0.92	2.06	1.45	0.69	0.075	13
4.13	0.64	1.28	1.95	1.47	0.67	0.26	11
9.5	7.67	0.9	4.86	1.22	1.77	0.02	15.4
4.59	1.3	0.74	2.77	1.16	0.92	0.136	7.8
3.4	0.93	0.94	1.8	1.39	0.62	0.131	12.9
3.84	1.28	1.21	1.81	1.6	0.62	0.224	8.1
3.9	1.29	0.84	2.52	1.27	0.77	0.366	18.1
5.26	2.16	1.01	2.93	1.38	0.94	0.055	13.3
3.94	1.75	0.99	2.33	1.5	0.74	0.02	15.4
3.63	3.4	0.66	2.38	1.17	0.77	0.015	10.9
2.84	0.8	0.65	1.87	0.95	0.64	0.183	27.3
4.7	0.89	1.28	2.71	1.71	0.8	0.005	10.3

4.3	1.47	0.81	2.82	1.15	0.84	0.024	8.6
6	2.63	0.86	4.01	1.34	1.22	0.046	13.2
5.5	2.01	0.93	3.91	1.35	1.28	0.842	17.6
5.4	1.4	0.95	3.28	1.38	1.07	0.161	19.2
6.22	5.42	0.93	3.22	1.63	0.99	0.18	8.9
4.78	4.68	0.86	2.55	1.34	0.85	0.029	10.2
5.51	3.94	0.65	3.52	1.1	1.11	0.142	10
3.94	1.75	0.99	2.33	1.5	0.74	0.02	15.4
5.55	2.26	1.01	3.81	1.55	1.14	0.016	8.7
4.35	2.44	0.77	2.95	1.17	0.9	0.021	8.2
4.44	1.41	0.81	3.02	1.32	0.87	0.045	4.8
2.94	1.04	1.1	1.23	1.45	0.43	0.278	31.3
3.39	0.48	1.35	1.73	1.73	0.59	0.031	13.7
3.29	0.7	1.15	1.93	1.65	0.66	0.497	16.5
3.98	1.08	0.92	2.44	1.15	0.87	0.323	11.4
5.4	2.09	0.95	3.37	1.42	1.03	0.055	16
3.76	2.73	0.69	2.33	1.2	0.73	0.111	41.2
5.35	2.39	0.86	3.48	1.25	1.06	0.048	17
5.15	1.39	1.35	3.42	1.59	1.32	0.027	15.4
5.16	1.85	0.86	3.65	1.27	1.1	0.331	10.8
2.81	1.39	0.58	1.77	1.05	0.57	0.004	18.4
5.49	1.63	1.28	3.62	1.46	1.34	0.518	14
3.73	1.11	0.79	2.42	1.24	0.74	0.171	15.6
4.38	1.67	0.84	3.03	1.37	0.9	0.065	12.1
3.38	2.63	0.96	1.94	1.49	0.69	0.014	5
4.93	1.53	1.53	2.76	1.23	0.98	1.095	8.8
4.55	3.75	0.76	2.39	1.15	0.97	0.196	7.9
2.99	1.02	0.87	1.66	1.43	0.6	0.076	10.7
4.81	0.58	1.29	3.12	1.44	1.05	0.059	18.7
5.38	2.53	0.7	3.65	1.25	1.18	0.057	16.6
5.08	1.74	0.84	3.53	1.22	1.15	0.371	9
6.83	5.41	0.98	4.22	1.54	1.4	0.854	19.5
6.66	2.3	1.15	4.17	1.48	1.25	0.05	11.5
4.96	0.89	1.1	3.08	1.47	0.93	0.296	9.8
3.82	1.81	0.76	2.32	1.32	0.72	0.502	16.2
4.41	1.63	0.97	2.98	1.41	0.91	0.007	12.5
4.84	1.35	0.72	3.38	1.14	0.99	0.23	14
6.12	5.5	0.79	4.47	1.15	1.33	0.053	13.8
4.8	5.1	0.77	2.99	1.25	1.06	0.292	25.5
5.99	4.33	1.06	3.36	1.61	1.09	0.207	11.8
4.28	0.87	1.25	2.52	1.66	0.84	0.745	15.3
4.66	1.87	0.9	3.19	1.3	1.01	0.076	11.1
5.04	2.15	0.8	3	1.33	0.89	0.115	11.5
6.63	1.52	1	4.42	1.39	1.3	1.003	11.2
4.1	2.29	0.97	2.22	1.54	0.79	0.17	9.4
4.22	1.85	0.97	2.57	1.42	0.73	0.151	7.1

4.25	4.66	0.69	2.36	1.16	0.82	0.075	9.1
4.49	0.9	0.92	2.98	1.27	0.86	0.123	6.6
4.47	0.62	1.64	2.15	1.9	0.65	0.007	16.3
4.57	7.3	0.92	2.37	1.48	0.79	0.011	20.4
3.14	1.62	1.01	1.88	1.22	0.64	0.165	6.6
2.85	1.52	0.63	1.6	1.01	0.64	0.073	26.2
6.42	1.18	0.96	4.45	1.36	1.44	1.381	12.2
4.64	3.43	0.86	2.56	1.4	0.79	0.058	20.6
8.05	10.85	1.06	3.24	1.72	1.07	0.052	15.6
5.9	2.25	1.04	3.74	1.45	1.13	0.262	12.1
4.86	1.13	1.02	3.19	1.44	0.95	0.437	12.2
4.72	3.08	0.75	2.91	1.26	1	0.035	8.2
6.36	0.77	2.58	2.96	2.4	0.88	0.071	12.1
2.57	2.05	0.47	1.3	1.08	0.47	0.013	10.4
3.56	0.64	0.9	1.99	1.4	0.36	0.019	9.7
5.96	1.34	1.2	4.46	1.27	1.6	0.182	17.5
6.02	1.46	1.73	3.61	2.09	1.16	0.125	15.4
4.63	1.29	0.9	2.8	1.33	0.84	0.433	14.2
5.48	1.32	0.97	3.59	1.45	1.02	0.21	14.3
4.7	0.91	0.98	3.03	1.35	0.89	0.458	14.5
6.2	3.22	1.14	3.37	1.52	1.2	0.486	9.8

Hab1c	FPG	2h-PG	FCP	FINS	2HC-P	2h-ins	CK
11.3	5.9	11.79	0.3	2.22	1.72	15.23	66
10.9	6.18	5.46	1.34	8.39	1.6	15.45	288
6.4	6.87	11.56	2.71	13.65	6.8	52.94	69
9.1	4.91	8.92	1.88	11.17	5.03	41.36	169
10.7	9.13	19.75	1.4	9.7	3.9	32.8	106
6.6	4.65	8.57	0.91	20.12	4.03	50.38	71
10.4	4.75	6.02	0.21	1.31	2.26	10.87	38
9.6	5.16	12.75	1	9.73	2.43	27.51	60
7.5	6.85	8.42	2.74	12.81	4.01	21.96	59
10.9	6.92	9.07	1.51	9.07	1.76	23.97	44
10.7	5.19	9.15	1.45	7.87	3.32	67.57	29
10.6	5.21	13.76	0.2	0.79	1.68	7.59	133
9.4	5.97	11.3	3.89	21.57	13.91	208.9	82
9	5.23	12.07	1.74	15.4	4.76	33.64	72
12.3	6.7	3.74	1.81	6.5	1.67	6.8	55
8.1	6.65	8.1	2.41	8.72	4.52	31.52	236
9.7	7.43	12.14	0.96	2.34	3.45	41.08	87
10.2	10.42	12.05	4.03	33.11	6.15	142.8	79
9	6.55	11.25	0.88	2.16	2.93	55	102
11.8	8.71	15.47	1.44	6.36	2.13	23.94	65
8.4	7.38	11.67	3.85	35.38	7.11	84.47	80
11.3	7.11	15.15	1.42	7.79	4.18	66.84	86
7.6	5.27	10.88	1.58	16.72	5.2	82.46	102
7.8	5.4	10.17	0.73	8.8	4.65	26.44	88
12.8	9.14	8.76	2.37	17.3	2.17	59.75	76
9.1	5.93	6.28	2.87	9.1	4.11	30.07	153
8.6	4.92	4.18	0.15	0.83	0.07	0.6	39
6.6	3.84	9.45	0.41	50.53	1.13	52.97	125
11	6.13	7.27	0.77	16.33	0.81	62.08	141
11.3	7.76	8.19	1.16	5.37	1.3	10.64	32
10.1	6.01	9.13	2.01	11.11	4.77	45.09	58
6.5	6.53	7.21	1.59	7.92	2.43	14.41	64
7.6	5.39	12.32	1.66	10.38	4.94	51.62	73
12.4	7.53	7.84	1.07	3.37	1.16	23.72	57
11.1	9.02	15.69	1.51	15.07	2.73	98.27	89
9	3.83	5.99	0.19	1.27	2.38	11.76	39
6.6	6.27	15.21	1.67	9.21	5.54	74.76	52
8.9	6.93	7.98	0.23	63.88	0.84	64.69	82
8.5	6.59	10.64	0.47	38.23	1.44	59.66	210
9.4	6.16	8.37	1.47	6.09	2.34	18.73	44
13.5	5.87	5.16	1.56	5.3	1.81	10.29	74
11.1	5.36	9.76	1.72	8.33	8.49	45.47	55
6.8	7.97	14.18	2.08	10.67	4.49	65.64	99
9.2	7.74	9.93	1.63	9.11	3.3	39.26	133
10.3	6.83	8.35	2.83	12.9	5.82	20.01	84

8.6	11.42	13.8	0.94	9.97	1.23	46.75	56
9.9	5.69	6.07	0.74	2.41	2.06	26.03	28
9.3	6.45	7.56	2.62	12.28	6.45	35.63	49
10.3	7.57	11.31	2.87	16.57	5	65.11	65
6.7	7.33	8.08	0.47	2.28	1.63	8.12	80
6.9	6.17	12.78	1.96	15.19	7.65	112.2	70
10.8	6.53	13.28	1.56	10.9	2.29	73.47	75
9.1	6.84	12.15	1.22	14.73	3.13	27.28	59
8.2	8.16	14.45	4.56	34.8	10.41	180.2	37
9.7	6.41	9.7	2.23	8.59	6.16	35.63	131
10.9	7.37	13	1.56	14.46	2.31	31.09	118
10.1	6.17	10.63	0.14	0.88	0.32	13.87	55
9.4	5.16	5.39	4.52	16.52	5.73	71.74	78
7.8	7.57	13.23	1.88	6.45	2.58	12.92	100
11.4	9.21	12.65	1.55	6.99	1.64	14	57
14.6	4.58	10.15	0.96	4.65	2.79	36.53	45
13	4.74	7.13	2.54	25.44	2.86	33.96	98
7.8	6.29	9.67	2.19	7.41	5.74	17.63	184
9.6	7.25	9.05	4.27	21.47	8.33	76.73	58
6.7	5.93	12.24	2.4	13.27	10.12	116	109
11.3	5.17	9.83	1.93	5.28	6.4	30.83	68
11.3	4.92	6.57	2.66	7.86	4.69	21.99	51
10.6	5.2	7.23	1.96	8.56	6.69	48.01	81
13.8	4.5	12.08	1.24	2.5	4.45	20.26	57
8.9	5.85	9.59	0.14	1.73	1.29	36.85	93
8.6	4.95	6.1	0.19	1.19	1.34	5.47	65
9.7	5.8	9.5	1.65	21.01	3.42	38.74	96
12.1	6.49	6.96	0.75	2.57	1.91	10.95	70
7.8	5.58	12.13	1.86	38.46	4.68	58.29	85
9.5	6.97	11.1	2.41	7.26	5.98	38.2	57
10.4	6.25	9.47	2.1	13.39	7.04	91.73	45
6.4	7.22	10.02	3.22	64.65	5.15	36.78	38
15.8	7.13	13.41	0.12	0.69	0.24	4.63	34
8.4	4.65	5.41	1.36	9.19	0.8	6.53	79
11	8.55	6.35	0.21	0.81	0.07	0.83	56
7	7.34	13.95	1.76	11.3	6.49	77.89	74
10.2	7.82	14.54	2.72	12.49	7.79	71.41	140
8.9	6.49	15.39	1.36	5.18	5.13	30.59	35
12.9	7.78	9.3	1.62	2.99	3.66	17.89	74
9.7	7.58	16.9	0.37	3.49	2.44	24.11	89
15.1	4.59	7.55	0.21	9.03	0.14	10.41	82
12.3	6.77	5.93	1.64	3.93	2.25	8.27	39
12	7.62	16.44	2.54	12.7	6.01	71.74	100
11.3	9.84	16.44	1.98	8.41	5.36	35.49	75
11	6.94	10.42	1.68	4.6	3.08	15.5	69
9	8.39	13.8	1.38	20.41	2.53	27.91	82

9.3	7.85	12.34	3.94	15.91	7.13	37.81	38
9.5	5.22	10.22	1.26	9.11	3.12	84.98	61
10.2	6.95	10.02	1.96	6.5	4.25	23.46	200
9.1	7.56	12.87	2.06	11.68	8.02	114.7	171
8.7	5.84	4.81	0.93	1.93	0.62	2.47	45
7.1	8.78	10.27	3.22	12.52	5.07	59.6	67
13.8	5.61	6.57	0.2	2.28	0.95	9.36	91
8.5	6.21	9.3	1.6	15.92	2.82	25.67	66
9.6	6.38	4.35	1.05	8.66	0.09	77.27	87
10.7	7.24	10.03	1.19	5.02	2.53	54.48	79
8.1	7.77	13.08	1.89	34.63	6.94	105.3	35
8.7	7.51	18.28	1.69	11.22	9.59	121.6	38
12.5	6.91	8.28	1.87	6.55	2.97	31.54	106
11	8.82	11.35	2.43	13.63	3.55	33.84	72
7.3	3.82	12.85	0.76	35.42	7.62	106.6	179
9.3	5.69	7.6	0.83	1.66	1.55	3.44	138
10.3	7.59	14.35	2.03	7.54	3.78	28.19	254
12.1	6.7	8.86	1.78	6.42	1.85	11.4	66
11.9	8.64	9.48	2.82	13.71	4.08	49.49	54
7.3	6.2	5.36	1.26	6.42	0.21	131.9	83
7.6	8.85	12.19	1.98	6.93	3.48	25.79	30
9	7.96	8.4	2.08	3.43	11.49	17.5	29
9	7.13	8.4	1.65	8.25	3.71	14.15	126
13.4	8.4	10.84	1.26	3.4	2.25	29.03	71
7.3	7.55	9.97	6.11	21.68	10.31	69.04	76
11.2	8.08	15.68	1.17	9.68	3.51	26.04	131
11	6.65	10.96	0.7	13.89	1.93	52.55	130
6.4	14.44	16.46	0.01	0.3	0.01	17.85	104
9.7	6.23	17.41	0.14	1.39	2.08	39.43	96
6.3	6.15	9.72	2.12	11.72	3.1	80.38	102
8.7	5.84	10.51	1.26	5.96	4.34	22.56	90
14	6.21	10.58	0.36	2.71	2.09	7.35	94
8.9	6.74	13.99	2.29	28.5	8.39	93.29	84
9.5	6.71	14.2	2.04	5.93	5.06	25.78	93
11.6	5.78	9.56	2.19	11.37	7.87	65.58	90
6.6	6.76	12.31	1.4	48.88	4.47	91.62	154
8	8.23	17.04	1	1.45	2.1	7.11	56
8.6	7.7	13.54	0.65	1.38	2.38	18.16	49
7.8	9.96	22.34	2.27	8.56	5.15	27.84	74
10.4	6.57	8.06	1.46	10.03	2.33	23.65	222
8.1	4.12	11.54	5.49	16.24	7.12	23.35	168
7.3	6.84	5.99	3.35	18.71	4.16	22.6	63
12.8	9.56	17.65	0.26	3.33	1.5	20.01	52
12.7	6.47	9.72	1.25	5.31	1.92	25.06	26
12.5	4.59	20.65	0.74	5.39	4.18	16.9	61
9.3	8.04	17.26	3.99	20.3	13.1	155.6	99

12.5	6.52	10.75	2.3	15.22	7.38	71.48	44
11.3	4.92	7.07	0.68	3.68	1.56	38.09	77
14.4	6.16	7.64	1.78	5.07	2.3	17.56	58
10	10.84	13.63	2.57	13.59	4.81	73.12	84
7.3	5.56	9.99	1.99	9.35	6.78	35.06	134
8.2	6.45	8.45	2.3	7.46	8.41	35.9	67
10.1	5.36	6.38	4.04	15.34	12.35	63.96	51
12.1	5.23	5.56	1.3	21.3	3.44	66.28	86
9	5.68	8.56	2.18	8.16	4.23	24.7	51
12.2	11.14	16.01	1.96	8.02	2.54	37.08	70
9	7.27	8.16	2.26	5.92	5.45	17.43	69
6.2	6.38	12.74	1.9	13.9	7.8	70.5	57
10.7	6.97	9.22	1.62	56.87	2.01	85.26	271
9.1	8.42	16.23	2.01	5.94	4.86	17.63	110
8.5	6.72	9.03	2.73	12.8	4.65	34.53	105
9	6.57	17.39	1.2	8.03	4.2	24.28	45
10.4	6.6	11.22	2.14	2.28	2.3	17.73	98
9	6.84	15.96	2.38	8.96	5.88	53.27	50
7.1	6.84	9.55	1.02	4.61	1.82	15.48	45
9.6	6.45	6.51	1.46	5.33	1.81	42.32	43
12	3.25	14.12	0.01	10.4	0.01	14.73	119
7.7	7.12	8.28	3.32	17.87	5.33	35.09	100
10.6	7.74	10.58	0.23	0.72	1.52	5.73	33
8.3	5.13	10	1.65	5.43	2.89	24.63	59
8.6	11.28	6.63	1.34	2.77	0.8	2.56	69
8.5	11.09	10.61	3.87	18.29	6.66	59.84	71
6.5	5.05	6.22	1.4	30.13	3.79	56.08	292
7.1	9.85	15.01	2.83	17.69	3.21	57.07	91
10.7	8.6	6.41	2.1	5.11	1.85	6.6	42
11	7.36	8.77	1.97	7.02	3.44	21.8	66
8.3	8.46	8.94	2.22	11.2	3.42	26.29	65
7.5	6.88	8.25	3	12.38	4.09	18.38	54
9.9	4.13	7.36	0.28	31.85	3.73	53.51	49
7.2	7.93	9.52	2.07	6.78	7.22	29.4	125
8.7	7.4	12.47	1.87	3.62	5.9	18.36	48
9	8.83	9.77	1.95	7.29	4.41	18.77	56
9.5	8.05	9.75	0.83	10.09	1.48	22.47	128
10.5	16.49	17.05	2.83	11.68	3.25	17.44	76
11.4	6.88	10.01	1.64	5.49	4.27	21.99	42
10	7.89	8.57	2.8	8.02	4.84	18.12	38
10.2	10.36	13.34	3.89	15.92	5.55	37.81	60
9.3	5.52	12.25	4.07	59.94	13.2	163.8	114
10.1	10.25	9.78	1.68	5.81	1.74	22.79	34
12.2	6.95	14.5	0.39	6.91	1.74	25.19	51
9.6	6.86	10.51	0.88	7.36	1.48	14.17	69
10	4.74	10	0.28	8.94	2.76	54.59	97

11.5	6.9	11.85	6.03	16.53	7.89	59.43	36
10.3	7.01	7.59	0.91	2.65	1.78	14.76	130
8	5.96	10.24	1.21	8.17	4.4	46.51	64
9.7	7.49	8.32	1.85	4.53	3.47	10.39	14
6	4.91	5.52	0.09	0.32	1.49	5.46	89
7.1	6.58	10.91	1.82	68.94	3.75	127.9	81
7.4	10.72	6.53	5.71	15.53	6.89	52.52	40
10.6	6.11	4.03	1.24	5.76	0.28	22.98	79
10.6	6.72	10.89	0.21	1.46	1.56	28.92	35
7.6	5.32	9.47	0.17	5.4	1.06	76.26	140
11	7.91	10.03	3.49	42.75	5	63.08	44
5.8	6.19	9.25	2.03	7.75	7.01	39.53	40
9.2	7.75	11.59	1.7	7.17	3.32	36.77	65
10.7	8.38	11.98	2.44	11.45	4.7	39.23	81
9	6.68	9.41	1.01	3.46	2.75	16.12	55
5.8	5.97	13.84	1.79	40.58	8.56	118.1	95
8.6	6.74	13.24	1.5	14.43	2.49	27.05	59
10.4	5.87	11.2	0.7	10.43	1.12	15.16	65
10.4	10.82	13.77	1.35	14.1	2.67	34.58	38
10.7	6.1	13.42	1.7	6.39	4.48	32.99	30
5.9	5.32	9.61	2.67	15.59	11.93	159.1	111
11.3	6.5	9	0.94	13.48	2.69	28.07	39
7	5.66	14.24	5.1	24.42	12.88	121.2	182
13.1	9.57	10.81	0.24	2.95	1.75	13.86	57
10.4	6.11	11.92	1.55	6.72	2.56	22.85	51
12.8	6.65	12.5	1.67	5.12	2.76	15.28	56
11	6.91	13.29	1.21	7.69	2.44	51.5	50
8.8	6.7	16.38	2.59	6.43	8.68	38.81	80
10.4	6.31	12.06	1.52	9.8	4.66	25.43	50
12.3	6.85	6.03	2.14	11.32	4.92	65.81	77
8.6	6.18	12.74	1.96	82.87	4.81	196.3	301
8.6	7.06	8.43	5.04	16.37	13.06	84.16	187
10.1	6.43	11.22	1.53	3.55	2.99	28.77	68
6.3	6.03	9.4	2.28	9.72	3.57	18.23	79
9.1	6.45	5.12	1.89	5.48	1.5	5.75	49
10	5.29	7.01	0.38	6.63	1.98	18.94	71
9.4	8.18	10.28	1.65	8.32	4.43	26.92	87
9.6	5.76	8.69	1.09	4.63	2.62	33.93	45
8.9	5.43	7.1	2.44	16.58	7.88	93.04	84
12.5	6.16	9.7	0.37	2.84	0.89	5.14	52
10.8	6.06	8.91	1.64	7.91	3.85	14.22	52
11.4	5.61	7.52	1.31	3.66	1.91	13.72	18
7.9	11.38	15.41	2.88	6.97	4.32	44.62	145
11.2	6.97	12.2	3.06	37.14	9.41	127.9	67
7.7	4.59	10.79	1.29	4.47	3.38	12.47	96
9.8	9.27	11.55	1.33	4.48	1.91	11.46	72

10.6	9.11	14.31	1.26	35.88	3.23	76.57	43
13	12.33	14.41	2.18	10.2	2.26	24.57	58
12	5.83	12.59	0.13	4.71	2.4	18.14	41
7.4	6.12	10.44	1.55	7.73	3.78	35.45	125
9.7	7.14	11.73	0.94	3.93	1.86	9.63	134
7	6.02	8.54	1.01	4.38	4.77	23.96	79
8.5	4.98	9.74	0.01	59.83	0.01	116	31
5.5	5.48	5.7	4.27	10.93	9.16	20.72	99
8.8	5.17	8.59	0.23	5.51	2.05	13.17	79
10.4	8.81	19.28	3.48	12.19	17.85	75.23	67
7.1	5.06	10.48	1.91	29.49	6.6	139.9	96
9.4	6.96	11.16	1.06	7	5.48	21.07	68
9.6	5.53	3.67	0.87	2.56	0.31	3.52	125
7.2	5.53	13.87	2.09	8.14	7.73	58.19	65
9.1	9.33	12.65	5.41	57.4	15.86	138	45
8.3	5.27	9.27	2.58	8.56	9.45	63.69	64
10	6.96	13.24	0.94	16.07	4.82	136.2	81
10.7	9.74	8.39	1.17	2.96	1.55	5.92	72
8.8	4.63	10.3	2.94	8.12	8.15	27.39	52
7.2	6.27	12.07	1.98	6.71	4.08	67.33	192
9.6	7.54	12.8	2.45	9.13	10.22	86.43	66
11.8	6.86	9.13	0.97	5.88	2.06	17.44	31
12.8	5.21	9.27	0.18	0.3	0.85	5.49	38
7.5	8.8	9.91	1.93	6.47	2.35	12.97	427
12	6.54	7.25	0.53	18.34	0.33	17.75	19
12.1	8.69	7.36	0.29	1.03	0.22	2.15	37
6.9	6.24	5.97	2.76	159.1	8.69	335	109
13.7	7.35	4.03	0.12	0.98	0.05	1.4	38
10.5	7.18	10.32	0.25	2.39	2.9	15.8	131
8.7	6.69	12.96	1.22	21.43	3.44	42.25	105
10.8	12.68	9.3	1.43	3.9	1.19	14.5	51
8.7	5.86	12.68	1.66	20.81	3.88	38.33	45
8.9	5.99	9.47	2.37	5.55	10	39.55	120
9.1	11.82	17.42	1.92	6.17	4.15	27.46	44
8.7	6.11	5.12	2.72	7.75	2.35	9.47	26
10.5	9.84	10.3	2.5	39	4.1	79.2	105
8.2	8.11	13.47	1.85	5.05	5.56	60.96	39
12.5	9.41	21.46	4.93	32.26	10.34	85.23	65
9.8	6.38	9.87	0.76	6.34	2.53	18.56	35
8.4	6.34	12.31	3.31	7.64	10.44	67.51	44
8.1	5.66	10.63	1.56	5.87	9.98	59.41	44
8.5	7.61	13.6	1.38	3.5	2.89	12.57	35
10.9	5.43	12.01	1.94	6.48	4.47	27.86	57
7.5	6.75	11.13	3.16	8.16	4.88	18.08	79
12	8.82	16.21	4.54	17.1	8.57	48.43	54
9.1	6.72	8.36	3.34	14.1	5.38	37.8	228

6.9	5.27	11.66	1.41	34.56	5.57	95.75	44
8.6	6.27	9.5	11.05	16.08	14.42	37.21	46
8.5	6.73	7.32	2.3	8.19	4.23	12.74	195
7.9	8.18	11.27	3.21	16.63	5.14	40.91	274
9	10.09	8.05	1.92	21.49	1.74	25.07	103
10	8.6	14.55	2.37	8.75	5.34	22	105
8.7	8.67	16.23	1.79	15.24	4.8	93.84	391
9.7	7.73	17.83	2.08	82.94	6.87	145.1	68
7.6	5.16	8.51	0.74	5.82	2.98	15.06	65
7.8	8.86	14.15	3.92	9.79	9.66	30.79	82
12.2	11.18	12.43	3.35	14.6	3.64	17.12	91
10.4	7.74	8.54	3.32	9.61	6.02	21.83	91
10.5	5.03	6	1.07	1.89	1.87	9.24	62
9.2	4.2	10.12	0.24	27.03	2.04	66.19	84
6.5	5.53	12.26	2.8	9.81	8.25	32.4	56
7.3	6.67	12.59	1.46	2.8	5.58	45.16	148
8.3	5.9	10.2	2.72	7.86	10.66	31.86	176
8.3	7.13	10.87	1.4	7.12	5.01	59.64	152
9.2	6.24	11.79	2.34	8.93	6.42	21.41	122
9	7.67	5.96	1.68	14.09	1.99	21.33	73
8.9	10.02	10.2	1.6	11.92	0.92	31.84	95
9.9	7.68	13.89	3.74	10.49	9.2	62.4	100
9.7	6.8	10.07	1	12.18	2.02	37.76	156
13	5.77	4.91	1.07	2.46	0.82	5.04	32
7.2	5.87	7.03	1.57	8.1	3.93	25.93	59
11.3	6.97	1.71	0.23	0.54	0.08	2.27	54
13	6.29	11.32	1.12	3.17	1.76	10.73	29
8.4	6.44	8.03	1.98	8.07	6.24	38.42	78
14.2	10.75	14.86	2.14	4.87	3.13	13.76	39
13.8	12.56	18.17	3.24	8.4	5.73	78.11	83
10.1	5.27	6.87	0.55	2.05	2.23	11.26	56
8.5	7.79	13.18	1.65	5.68	3.33	20.78	142
11.6	7.36	7.44	1.87	6.81	3.41	18.06	86
9.7	8.23	11.12	2.55	11.42	3.93	42.65	113
9.1	9.38	18.28	2.12	10.98	9.76	71.44	149
6	4.91	5.52	0.09	0.32	1.49	5.46	89
11.1	5.81	12.57	2.07	6.88	8.94	60.64	98
7.7	6.95	11.37	2.76	8.43	4.76	13.68	62
7.6	5.78	7.74	3.12	12.52	3.9	21.18	64
8.8	16.45	13.53	0.01	27.83	0.01	25.55	124
10.2	9.12	14.1	2.97	9.23	5.25	23.42	69
9.8	6.06	10.06	1.38	5.36	3.16	16.2	72
9.1	7.12	13.69	1.35	32.9	4.56	82.3	58
8.3	10.13	16.21	1.81	2.89	4.84	14.15	41
13.3	8.2	7.4	2.34	6.52	2.43	7.95	23
10.2	5.47	6.74	1.03	14.06	2.58	25.71	54

8.8	6.96	11.07	1.96	5.74	6.97	27.65	44
10.3	6.56	7.57	3.72	15.21	5.8	27.25	112
9.9	6.97	17.81	0.26	1.47	1.87	18.27	46
11.8	8.84	10.88	0.62	2.19	1.49	6.69	62
8.3	4.95	10.14	1.46	8.03	6.63	18.72	88
7.1	5.01	11.81	1.49	13.35	10.17	58.65	137
11.9	9.56	14.18	4.14	11.73	10.82	48.45	43
9.1	7.12	13.69	1.35	32.9	4.56	82.3	58
9.4	6.56	8.63	2.92	8.39	6.94	23.84	79
9.9	8.13	9.73	1.66	4.62	4.33	16.44	82
8.1	6.03	13.39	1.16	9.24	6.67	28.26	78
9.2	8.55	8.71	1.6	10.34	1.9	15.04	57
6.5	6.49	12.61	2.54	7.49	10.41	34.15	56
13.9	5.33	7.02	0.2	1.32	0.64	5.34	72
7.1	6.74	8.17	3.54	14.28	5.91	39.56	47
11.2	8.69	17.16	2.14	7.75	5.58	53.71	91
13.1	7.73	10.15	1.7	4.71	2.62	24.45	499
7.2	7.64	14.22	1.58	2.08	2.11	8.89	67
8.5	5.7	12.15	2.7	16.22	8.88	59.98	37
9	5.81	11.54	0.96	1.99	2.43	11.07	28
6.6	9.63	14.29	1.9	5.81	3.34	19.62	25
9.1	7.49	6.07	1.45	3.52	1.7	31.07	55
8.4	6.25	10.19	1.03	5.33	3.59	14.03	51
8.3	6.92	9.51	2.19	6.46	7.85	24.96	86
8.7	16.05	9.5	5.2	50.79	1.54	16.17	93
13	7.09	7.3	2.15	3.46	2.78	13.76	97
9.3	7.09	10.65	2.16	7.46	3.54	20.15	94
8.6	8.47	13.56	0.26	5.16	1.86	16.97	80
9.3	8.28	14.01	0.17	18.45	0.57	55.74	97
11.2	6.9	11.64	1.41	4.17	3.44	31.52	36
7.6	8.86	13.18	1.61	8.89	6.42	99.79	191
11.5	8.83	17.79	1.29	4.44	2.93	17.03	33
13.3	6.81	15.22	3.06	18.21	7.45	202.5	48
7.7	5.24	9.31	2.68	67.07	6.04	122.6	104
10.3	7.34	12.86	3.11	13.68	7.6	57.03	161
7.2	6.79	7.69	2.22	17.25	6.35	74.63	60
11.8	9.25	11.21	1.8	6.84	4.49	21.23	31
9.9	9.38	11.13	4.52	27.89	6.52	35.1	88
8.8	5.81	3.7	0.93	2.29	1.41	4.83	42
7.2	5.78	8.55	1.85	15.07	4.24	63.23	225
10.2	10.72	26.13	2.94	7.73	6.12	16.22	104
10.7	6.53	7.43	1.47	5.3	3.8	20.96	32
8.6	7.8	14.01	2.25	9.03	4.86	60.45	55
9.3	7.88	13.6	1.9	8.79	5.14	54.65	64
7.1	7.61	8.88	2.24	31.44	6.9	74.96	67
7	6.19	8.86	0.9	5.44	3.62	66.19	95

7.2	7.88	12.55	5.12	17.82	14.73	126.3	68
6.7	5.92	7.86	3.2	11.4	10.36	61.08	103
8.9	6.84	8.66	0.32	2.13	1.02	7.85	39
10.7	7.66	10.85	2.16	6.04	6.87	21.68	101
7	5.69	4.16	5.28	16.71	12.8	63.71	116
11.4	8.42	6.6	1.47	3.86	2.48	13.15	76
11.6	7.71	7.44	0.24	0.81	0.88	2.61	110
10	7.98	10.49	1.05	30.77	1.62	51.95	106
13.2	10.64	5.3	3	9.6	2.25	6.3	54
10	9.45	10.5	1.19	6.17	1.54	13.68	37
10.2	8.3	12.03	1.98	10.9	2.47	22.27	76
8.6	6.92	8.94	1.71	7.11	3.71	15.91	129
9.9	6.09	8.44	0.13	1.2	0.9	2.78	91
7.8	8.17	12.79	2.66	9.85	4.64	23.72	100
7.8	5.67	9.39	2.43	6.6	2.47	48.9	99
13.5	8.3	9.88	0.55	1.82	1.46	8.41	30
9.8	7.54	15.96	1.74	11.43	6.21	55.57	121
7.1	12.31	20.27	0.01	3.98	0.01	14.22	131
9.8	7.01	16.91	2.66	12.13	13.9	168.4	61
9.7	7.85	13.31	1.96	7.12	5.5	49.36	78
6.4	6.8	5.6	5.49	10.71	6.16	13.58	129

CK-MB	LDH	HBTH	hs-CRP	Mb
13	179	111	0.86	10
20	182	114	0.47	65
12	152	85	2.19	17
29	178	111	1.67	33
14	172	115	1.59	42
12	194	124	0.31	14
5	123	76	1.38	17
8	158	106	5.51	18
9	123	81	1.62	13
25	150	86	1.76	9
11	120	71	2.57	15
16	207	135	0.34	10
11	152	86	3.37	19
9	184	105	1.27	20
10	162	101	3	17
15	175	110	1.41	21
15	162	104	3.31	16
11	205	111	6.13	13
11	187	119	1.59	31
12	174	99	2.02	10
9	172	110	1.97	15
13	162	100	1.75	11
17	204	133	1.66	26
12	180	118	0.32	14
22	178	100	4.57	23
13	220	142	3.79	15
26	164	106	1.09	8
86	214	154	0.8	65
15	230	147	4.02	23
13	159	109	1.03	11
8	154	94	0.78	13
10	173	110	1.35	21
13	142	94	1.99	38
16	148	96	0.43	8
12	169	112	0.95	22
14	140	91	4.85	17
32	151	94	0.92	11
17	176	132	0.99	32
10	166	100	5.52	31
10	138	89	1.23	13
12	159	88	1.81	15
19	229	149	1.64	39
12	175	109	0.74	33
15	163	100	5.83	15
14	188	104	6.71	28

9	164	97	0.49	16
11	176	98	10	13
12	207	111	10	8
9	145	90	3.89	11
13	196	129	1.68	18
8	182	112	2.02	43
13	156	94	0.3	11
14	158	97	0.64	15
11	136	84	10	16
10	183	94	4.72	23
16	214	134	0.69	59
13	179	109	0.81	18
12	165	97	3.17	26
13	168	99	1.79	19
12	172	106	2.85	12
14	188	125	5.48	20
15	233	153	0.96	94
10	168	102	0.95	32
9	129	73	2.36	21
13	191	117	1.16	31
14	196	113	2.46	16
12	123	78	10	12
10	202	112	5.61	16
9	149	97	0.25	21
10	220	136	1.11	23
12	166	99	3.28	31
19	135	89	2.62	25
13	151	95	3.01	32
12	166	111	0.84	17
14	155	84	2.08	16
15	173	99	4.09	17
11	143	102	6.15	22
14	119	70	10	16
9	229	149	1.01	32
12	181	111	3.84	19
9	113	76	2.88	28
16	202	127	1.2	37
13	150	86	2.43	16
18	164	98	1.03	15
13	148	89	2.76	27
15	179	123	10	35
17	132	80	0.57	19
14	157	97	0.88	43
9	187	106	1.85	32
10	184	106	2.13	39
12	150	92	1.27	22

9	150	76	5.23	15
10	192	122	1.5	40
13	173	106	1.17	30
15	184	90	3.15	20
10	162	101	2.17	14
13	168	106	0.72	21
11	217	142	4.8	45
10	150	92	1.78	21
12	175	107	1.12	51
11	182	97	2.31	24
9	112	74	0.86	22
13	186	103	10	32
13	157	95	1.74	34
10	162	99	3.75	19
22	218	142	2.7	52
10	177	110	3.45	30
13	229	129	1.65	38
7	194	115	3.93	34
9	155	107	3.93	24
12	158	92	1.73	25
9	135	81	10	21
14	127	86	10	17
15	170	108	2.66	32
12	133	80	0.33	45
9	148	92	1.28	20
18	181	114	1.23	25
13	163	96	0.38	31
13	182	107	2.77	42
11	185	116	0.74	18
7	191	121	4.48	39
13	166	98	1.28	25
11	163	114	0.56	46
19	189	113	1.31	24
13	156	98	0.82	24
9	161	98	10	70
19	154	95	3.35	30
9	144	90	0.59	44
9	166	104	4.28	28
12	158	93	3.15	19
14	310	183	0.67	41
37	213	134	2.26	96
10	189	108	1.77	20
13	153	101	0.46	20
15	179	117	10	11
11	126	73	2.72	19
19	224	129	2.9	22

26	166	93	9.41	14
8	171	101	10	44
12	129	74	1.74	21
16	144	90	2.02	16
9	158	96	0.91	45
11	207	132	3.26	12
14	167	83	10	22
12	140	84	0.57	19
11	168	108	1.77	21
16	180	116	2.86	44
12	129	77	1.01	22
9	146	93	0.28	52
12	195	126	2.01	61
14	174	107	1.74	25
76	164	94	7.33	24
12	128	72	2.78	23
16	216	142	3.64	41
13	145	94	1.08	27
19	211	141	0.65	25
8	207	121	1.93	22
18	218	142	1.14	29
11	147	95	1.26	46
6	133	101	1.5	18
5	214	140	2.54	59
9	217	143	2.59	22
11	122	74	2.59	21
17	195	131	0.41	88
13	161	97	4.81	31
7	129	77	2.92	23
8	189	98	4.33	26
12	141	92	1.54	20
8	149	88	2.94	28
10	153	102	1.31	17
7	161	110	3.32	18
10	121	81	0.76	25
11	134	79	10	19
14	238	170	0.54	27
9	139	90	1.71	24
8	107	63	0.96	24
7	139	95	0.38	22
9	138	101	2.22	16
12	190	121	1.12	82
18	176	113	1.97	10
15	171	110	1.14	11
18	190	108	3.7	14
17	158	98	1.44	17

12	201	128	10	49
12	170	95	1.23	21
20	165	105	1.29	15
48	15	172	10	9
11	182	117	0.98	11
15	163	99	2.46	24
18	180	104	4.92	24
15	140	76	6.35	17
9	175	126	0.66	13
13	176	108	1.46	20
14	182	93	6.85	7
17	131	80	1.02	14
15	168	106	1.69	15
11	144	92	2.45	34
10	124	74	3.32	8
12	180	108	0.67	32
10	189	114	0.54	15
15	151	103	0.47	19
11	127	79	1.31	14
15	150	104	2.6	11
17	162	111	0.74	21
13	126	81	0.71	11
14	198	118	5.06	22
14	162	116	0.74	10
18	145	88	10	15
15	160	98	1.85	15
10	131	85	10	11
16	171	101	1.73	18
13	175	110	2.02	9
12	120	64	2.01	14
11	192	123	3.21	76
13	173	97	0.96	42
15	205	133	2.75	28
11	181	128	0.35	24
12	124	84	1.82	29
13	194	120	0.92	15
16	211	134	4.04	14
11	147	98	1.48	9
10	179	106	1.94	11
20	158	90	0.66	19
11	132	82	1.28	9
10	193	118	4.81	14
18	166	109	0.62	31
14	169	93	2.69	33
11	179	109	2.31	21
8	148	98	1.7	33

13	176	109	1.67	22
9	175	104	2.82	16
9	149	98	0.33	17
8	163	107	2.11	24
21	153	102	0.29	21
8	166	111	0.74	18
15	186	113	9.27	27
13	201	113	10	33
17	164	102	1.18	30
9	186	105	1.14	36
13	144	82	0.75	20
6	167	97	0.64	18
11	224	142	1.45	40
7	155	92	1.05	29
12	147	98	0.53	26
12	163	110	0.62	24
10	110	73	0.16	37
14	144	102	0.24	38
9	123	81	2.35	27
9	178	115	0.94	72
10	209	117	1.42	24
13	119	77	0.5	12
8	151	106	2.68	36
9	207	118	10	91
6	144	102	0.97	17
9	131	90	0.83	8
14	223	138	2.73	21
11	131	84	5.19	9
10	188	118	0.89	23
13	168	115	0.47	28
6	160	113	2.9	25
12	149	107	9.13	26
9	184	120	1.35	28
10	165	106	1.88	24
8	120	76	10	24
18	208	121	10	27
10	142	90	4.43	11
12	251	178	6.06	28
10	142	95	0.48	14
9	145	91	0.73	27
9	132	89	8.39	16
15	126	83	0.84	12
6	155	103	1.29	19
11	173	102	2.14	31
12	151	98	1.81	42
12	138	85	7.66	51

10	190	124	1.84	37
11	142	83	6.74	48
19	162	107	1.61	37
16	198	148	2.27	110
9	145	85	8.02	27
11	167	109	1.49	24
17	232	149	10	51
11	195	129	9.77	20
7	123	85	10	19
8	211	133	1.3	22
13	140	92	3.77	14
14	143	96	4.19	18
9	124	79	2.99	19
12	156	109	0.27	28
11	142	87	10	19
13	176	118	1.43	48
12	167	99	1.58	57
10	135	84	0.88	23
10	154	105	10	44
13	153	107	1.72	38
10	214	151	1.64	26
14	146	92	0.61	22
7	147	96	0.53	28
7	139	95	0.51	10
10	152	103	1.68	22
27	183	115	0.53	15
9	175	110	0.26	9
10	142	87	0.4	15
7	141	90	4.72	53
15	218	147	5.3	47
10	212	141	0.92	20
11	160	109	1.01	31
10	179	122	1.29	20
10	200	129	2.35	36
15	149	93	1.69	57
11	182	117	0.98	11
11	140	86	1.94	21
9	120	80	0.49	14
7	159	101	0.95	12
9	291	190	10	26
7	136	91	0.74	22
9	111	69	0.62	27
10	148	87	0.56	40
12	142	92	1.37	29
7	223	140	10	15
11	215	150	1.27	19

7	129	88	0.45	13
10	187	110	6.87	26
8	179	121	3.07	25
10	150	95	2.58	22
13	223	143	1.24	17
11	152	94	0.76	38
9	148	89	10	20
10	148	87	0.56	40
13	155	91	1.85	19
6	131	86	2.08	14
12	140	94	0.38	31
8	205	140	5.34	34
14	132	87	1.03	18
10	155	108	0.35	15
11	162	92	10	35
11	167	102	1.86	27
16	170	117	1.47	250
10	145	91	3	22
8	172	106	6.86	12
7	128	99	1.3	32
23	325	192	1.34	25
13	162	113	1.85	60
7	121	82	1.89	25
15	204	126	2.63	31
14	108	62	1.46	18
8	175	124	10	50
10	162	99	10	36
9	175	117	1.98	36
10	163	115	3.13	32
10	128	74	2.06	24
9	161	108	0.47	32
11	205	139	10	28
9	148	90	7.21	40
9	156	95	2.95	54
8	162	104	1.38	35
9	177	108	1.98	33
14	132	86	1.97	15
10	203	117	1.55	18
9	178	114	2.83	23
12	197	121	1.12	59
8	223	149	1.24	26
11	144	94	1.11	17
5	183	102	1.53	22
9	147	84	3.08	18
10	123	75	10	31
9	157	109	1.43	23

10	149	95	0.92	28
13	169	109	1.28	20
12	143	81	0.82	16
12	163	98	2.44	20
13	137	86	0.33	18
9	176	107	10	32
11	167	112	1.84	55
17	210	137	4.23	29
13	147	85	2.67	20
10	174	114	1.67	17
7	152	98	3.69	27
11	157	103	0.9	30
16	168	115	0.49	26
11	174	121	1.51	77
22	285	178	1.23	19
13	157	109	1.37	22
8	186	128	1.03	15
13	141	90	0.96	44
13	163	102	1.54	28
12	169	118	0.36	18
12	219	143	10	26
