# Supplementary material for: Factors related to type 2 diabetic retinopathy and their clinical application value
Source: Front Endocrinol (Lausanne). 2024 Nov 20;15:1484197. doi: 10.3389/fendo.2024.1484197 (PMC11614660; doi:10.3389/fendo.2024.1484197)
Supplement: Supplementary file 2 [file Table1.docx]

Supplementary Material

# Supplementary Data

Table S1. Differences in the distribution of various factors between the two groups of patients

|  | **Total(n=380)** | **DR(n=154)** | **NDR(n=226)** | **P** |
| --- | --- | --- | --- | --- |
| Age | 55.00(47.50,64.00) | 57.00(52.00,64.00) | 54.00(43.00,65.00) | 0.078 |
| BMI | 25.25(23.26,28.09) | 24.58(23.14,26.81) | 25.67(23.46,28.40) | 0.030 |
| SBP | 136.00(12500,15100) | 142.50(128.00,157.00) | 133.50(125.00,144.00) | 0.001 |
| DBP | 89.00(81.00,96.00) | 90.00(81.00,99.00) | 88.00(81.00,95.00) | 0.340 |
| COD | 96.00(36.00,180.00) | 120.00(72.00,216.00) | 81.00(24.00,144.00) | <0.001 |
| HOMA-IR | 2.48(1.50,4.38) | 2.37(1.40,4.17) | 2.77(1.56,4.43) | 0.212 |
| TyG | 2.18(1.68,2.72) | 2.18(1.73,2.66) | 2.18(1.67,2.79) | 0.518 |
| WBC | 6.23(5.17,7.18) | 6.23(5.41,6.93) | 6.23(5.14,7.28) | 0.847 |
| RBC | 4.84(4.45,5.24) | 4.74(4.36,5.09) | 4.97(4.52,5.35) | 0.001 |
| HB | 145.00(133.00,157.00) | 142.00(131.00,152.00) | 150.00(135.00,159.00) | <0.001 |
| PLT | 224.00(191.00,265.75) | 224.00(190.00,270.00) | 224.50(192.00,265.00) | 0.897 |
| FIB | 2.89(2.52,3.41) | 2.99(2.58,3.52) | 2.80(2.46,3.27) | 0.028 |
| DD | 0.19(0.12,0.33) | 0.21(0.13,0.37) | 0.19(0.12,0.32) | 0.159 |
| ALT | 20.50(15.00,30.00) | 18.00(14.00,25.00) | 22.00(16.00,34.00) | <0.001 |
| AST | 19.00(15.00,23.00) | 18.00(15.00,22.00) | 19.00(16.00,27.00) | 0.026 |
| AST/ALT | 0.90(0.70,1.10) | 1.00(0.20,1.20) | 0.90(0.70,1.10) | 0.001 |
| TP | 69.15(65.90,72.70) | 68.70(65.60,72.30) | 69.75(66.30,73.10) | 0.096 |
| ALB | 43.20(40.63,45.10) | 42.45(39.90,44.50) | 43.45(41.20,45.60) | 0.001 |
| GLB | 26.28(0.19) | 26.41(3.71) | 26.19(3.78) | 0.592 |
| A/G | 1.60(1.50,1.80) | 1.60(1.40,1.80) | 1.70(1.50,1.80) | 0.018 |
| TBIL | 13.30(10.40,16.58) | 12.85(9.70,15.70) | 14.15(11.20,17.20) | 0.004 |
| CHE | 9567.50(8225.50,10928.50) | 9343.00(8121.00,10822.00) | 9744.50(8258.00,11019.00) | 0.213 |
| ALP | 81.50(66.00,100.00) | 83.00(66.00,102.00) | 80.50(66.00,99.00) | 0.506 |
| GGT | 26.00(18.00,41.00) | 24.00(17.00,34.00) | 28.00(19.00,45.00) | 0.024 |
| PA | 0.29(0.01) | 0.29(0.06) | 0.29(0.06) | 0.758 |
| UREA | 5.50(4.60,6.67) | 5.89(4.90,7.21) | 5.33(4.45,6.50) | 0.001 |
| CREA | 60.85(52.38,72.00) | 62.50(54.40,76.50) | 59.65(51.20,70.20) | 0.080 |
| eGFR | 103.47(93.62,112.32 | 100.66(90.45,110.49) | 105.74(94.98,115.28) | 0.002 |
| UA | 303.00(246.68,370.10) | 299.00(253.10,352.10) | 306.10(241.30,376.50) | 0.365 |
| GLU | 11.04(8.58,15.02) | 11.08(8.08,15.70) | 10.99(8.84,14.35) | 0.865 |
| Ca | 2.32(0.01) | 2.31(0.10) | 2.32(0.09) | 0.147 |
| P | 1.14(1.02,1.26) | 1.16(1.04,1.29) | 1.13(1.01,1.24) | 0.103 |
| Na | 137.00(134.90,139.00) | 137.20(134.90,139.20) | 136.70(134.90,138.90) | 0.739 |
| K | 4.11(3.89,4.36) | 4.13(3.93,4.34) | 4.10(3.88,4.37) | 0.477 |
| Cl | 102.50(100.30,104.40) | 103.00(100.70,104.90) | 102.30(100.50,104.30) | 0.077 |
| CO2 | 26.20(24.23,27.70) | 26.20(24.00,27.40) | 26.20(24.40,27.90) | 0.275 |
| TCH | 4.60(3.92,5.40) | 4.64(3.73,5.49) | 4.55(4.00,5.26) | 0.916 |
| TG | 1.54(1.13,2.29) | 1.49(1.17,2.09) | 1.63(1.11,2.53) | 0.395 |
| HDLC | 0.93(0.78,1.08) | 0.95(0.80,1.11) | 0.92(0.77,1.06) | 0.146 |
| LDHC | 2.58(2.08,3.16) | 2.59(1.94,3.24) | 2.58(2.17,3.08) | 0.760 |
| APOAI | 1.36(1.21,1.50) | 1.39(1.25,1.52) | 1.33(1.19,1.47) | 0.021 |
| APOB | 0.86(0.70,1.02) | 0.86(0.67,1.03) | 0.87(0.72,1.01) | 0.386 |
| APOa | 0.10(0.04,0.24) | 0.12(0.06,0.26) | 0.09(0.04,0.22) | 0.079 |
| ADA | 12.90(10.03,15.98) | 13.05(10.40,16.50) | 12.65(9.90,15.40) | 0.210 |
| HbA1c | 9.40(8.30,10.70) | 9.40(8.10,10.80) | 9.30(8.30,10.70) | 0.961 |
| FPG | 6.84(5.93,7.91) | 6.78(5.96,8.40) | 6.84(5.93,7.74) | 0.627 |
| 2h-PG | 10.29(8.42,12.84) | 10.26(8.37,12.50) | 10.45(8.51,13.29) | 0.319 |
| FCP | 1.69(1.07,2.41) | 1.56(1.00,2.37) | 1.87(1.16,2.44) | 0.035 |
| FINS | 8.33(5.21,14.10) | 7.97(5.11,12.52) | 8.63(5.30,15.07) | 0.143 |
| 2h-CP | 3.74(2.07,5.91) | 2.78(1.70,4.66) | 4.47(2.54,6.87) | <0.001 |
| 2h-ins | 28.52(17.20,59.22) | 24.13(14.50,52.52) | 32.33(18.16,62.40) | 0.008 |
| CK | 71.00(51.25,99.00) | 72.00(52.00,100.00) | 70.50(51.00,97.00) | 0.795 |
| CK-MB | 12.00(9.00,14.00) | 12.00(9.00,15.00) | 11.00(9.00,13.00) | 0.199 |
| LDH | 163.00(145.25,183.00) | 168.00(150.00,190.00) | 162.00(144.00,180.00) | 0.009 |
| HBTH | 102.00(91.25,115.00) | 107.50(97.00,118.00) | 99.00(89.00,112.00) | <0.001 |
| hs-CRP | 1.74(0.96,3.25) | 1.70(0.95,2.92) | 1.77(0.98,3.35) | 0.499 |
| Mb | 22.00(17.00,32.00) | 24.00(18.00,32.00) | 21.50(16.00,31.00) | 0.122 |
| Gender | 241(63%) | 94(61%) | 147(65%) | 0.426 |
| Smoke | 106(28%) | 42(27%) | 64(28%) | 0.823 |
| Drink | 117(31%) | 43(28%) | 74(33%) | 0.318 |
| FH | 167(44%) | 75(49%) | 92(41%) | 0.123 |
| HBH | 174(46%) | 84(55%) | 90(40%) | 0.005 |
| Intervention | 331(87%) | 146(95%) | 185(82%) | <0.001 |
| PN | 359(94%) | 149(97%) | 210(93%) | 0.108 |
| NV | 261(69%) | 122(79%) | 139(62%) | <0.001 |
| BVP | 267(70%) | 127(82%) | 140(62%) | <0.001 |
| LEVD | 360(95%) | 150(97%) | 210(93%) | 0.055 |

DBP: diastolic blood pressure; HOMA-IR: Insulin resistance index; TyG: Triglyceride Glucose Index; WBC: White blood cell count; PLT: Platelet count; DD: D-Dimer; TP: Total protein; GLB: Globulin; CHE: Cholinesterase; ALP: Alkaline phosphatase; GGT: Glutamyl transpeptidase; PA: Prealbumin; CREA: Creatinine; UA: Uric acid; GLU: Glucose; Ca: Calcium; P: Phosphorus; Na: Sodium; K: Potassium; CO2: Total carbon dioxide; TCH: Total cholesterol; TG: Triglycerides; HDL-C: High-density lipoprotein cholesterol; LDH-C: Low-density lipoprotein cholesterol; APOB: Apolipoprotein B; APOa: Lipoprotein a; ADA: Adenosine deaminase; HbA1c: Glycosylated hemoglobin; FPG: Fasting blood glucose;2h-PG:2-hour postprandial blood glucose; FCP: Fasting C-peptide;2h-CP:2-hour postprandial C-peptide; CK: Creatine kinase; CK-MB: Creatine kinase isoenzyme; hs-CRP: High-sensitivity C-reactive protein; MB: Myoglobin; FH: Family history; PN: Peripheral neuropathy; LEVD: Lower extremity vascular disease;

*For continuous variables, those following a normal distribution are presented as mean (standard deviation), and their significance levels are calculated using the t-test. Variables that do not follow a normal distribution are expressed as the median (P25, P75) and their significance levels are calculated using a non-parametric test. Binary categorical variables are reported as the number of cases (percentage), with their significance levels determined using the chi-square test.
